# Directly Observing and Characterizing Adolescents' Self-Generated Social Media Posts: Protocol for Creation and Implementation of a Cyberethnography Informed Codebook

**DOI:** 10.2196/84461

**Published:** 2026-03-31

**Authors:** Kylie Boyd, Lydia Bliss, Tingting Fan, Kayla Kern, Caitlin M Carlson, Megan A Moreno, Christopher N Cascio, Ellen Selkie

**Affiliations:** 1 Department of Pediatrics, School of Medicine and Public Health, University of Wisconsin-Madison, 800 University Bay Drive, Suite 310, Madison, WI, 53705, United States, 1 734-660-1362; 2 School of Journalism and Mass Communications, University of Wisconsin-Madison, Madison, WI, United States

**Keywords:** methodology, social media, adolescents, qualitative coding, codebook creation, cyberethnography, longitudinal observational study

## Abstract

**Background:**

Adolescent social media research has primarily focused on frequency of platform use and self-report measures. There has been limited focus on the self-generated content posted by adolescents and how this might relate to their well-being.

**Objective:**

This study describes a researcher-observed codebook for characterizing adolescents’ self-generated content in a longitudinal sample.

**Methods:**

Participants in the study provided informed assent (and parental informed consent) for researchers to follow them and passively observe their self-generated content on Instagram (Meta Platforms), TikTok (ByteDance Ltd), Facebook (Meta Platforms), and X (formerly known as Twitter; X Corp). Guided by Bronfenbrenner’s social ecological biopsychosocial model, the research team created a codebook incorporating prior cyberethnographic observation of self-generated social media content. After codebook refinement, coders (research staff and student research assistants) were trained through multiple rounds of test coding, and the codebook was applied to participant data with periodic quality control measures to ensure interrater reliability.

**Results:**

This study was funded in early 2023 and began data collection in March 2023 and will conclude in 2027. So far, the interrater reliability agreement scores (AC1) between coders have shown strong interrater reliability. For Year 1, scores were Facebook 0.89, Instagram 0.89, TikTok 0.88, X 0.87, and combined 0.88; for Year 2, Facebook 0.95, Instagram 0.96, TikTok 0.96, X 0.96, and combined 0.96. This project provides replicable guidance to categorize social media data from adolescent participants using human coders who can contextualize content through longitudinal observation. The method that our team chose and followed paved the way for many strengths to be recognized as well as lessons learned by our team that allowed for adaptation and growth to occur while this study has been ongoing.

**Conclusions:**

Cyberethnography, the chosen method for this research protocol, has allowed this research project to collect self-generated content for adolescent social media in a comprehensive manner. Thus, allowing our team to be able to cross-analyze this data with the well-being data that are being collected under the grander project for patterns. Sharing our protocol through this paper will also allow other researchers to draw from our methodology for future projects to aid in social media and adolescent understanding.

## Introduction

### Background

Social media has become a cornerstone of adolescent daily life, with 96% of US teens aged 13‐17 years reporting daily internet use and 46% engaging with platforms “almost constantly” [1]. These platforms (eg, TikTok [ByteDance Ltd], Instagram [Meta Platforms], Facebook [Meta Platforms], and X [X Corp]) offer adolescents unprecedented opportunities to interact with peers [2], explore their identities [3], and access global culture [4]. Although this study does cite from recent years (2023), we do recognize that social media data and information are constantly being updated, which has brought about a need for a study such as this to examine the type of content that adolescents post online.

Uncovering what specific content adolescents choose to post online is crucial, as it reflects lived experiences, developmental priorities, and cultural contexts [5,6]. These methods capture displayed content, which includes content analysis and ethnography. Within content analysis, there is objective coding but with limits to what meets our criteria [7]. Ethnographic studies of social media to date primarily focus on a narrow topic [8]. To address this gap, this study focuses broadly on cyberethnography of adolescents’ social media postings. Cyberethnography, by definition, is a type of research methodology that involves viewing media online and observing interactions in a digital space [9]. In this study, we conceptualize social media posting as self-generated content (SGC), defined as material that is created and shared by adolescents themselves on social media platforms. Though there is similar terminology, such as user-generated content (UGC), SGC differs in the sense that it is specific to what the individual participant posts versus just any form of content that is posted (retweets, reposts, etc). Our study also builds on previous social media coding tools such as the Application Programming Interface (API) or Python (Python Software Foundation) to use manual human coders instead of data scraping for data collection. Our research group proposes a cyberethnographic codebook designed to systematically categorize adolescent self-generated social media content across platforms, grounded in the lived experiences of teens’ posts [10].

Adolescence is a pivotal developmental period marked by milestones such as identity exploration [11,12], intimate friendship-building [13,14], and cultural socialization [15], all of which are commonly mediated through social media. Our codebook creates greater insight into these developmental periods through creation of a self-generated content categorization (coding) scheme informed by Bronfenbrenner (1979)’s ecological model [16]. Ranging from individual experiences to broader cultural systems, we unpack layers of contexts that may emerge from teens’ self-generated posts in the following section.

### Adolescent Social Media Posting and Development

Engagement with cultural trends, politics, global issues, and social norms situates teens within broader digital and societal contexts. Adolescents prepare for adulthood by understanding social norms and gaining insight into the world around them [17,18]. By posting sociocultural content, adolescents explore civic engagement and political views and test their values and beliefs within the context of society. Engaging with these topics online and participating in larger communities allows adolescents to express and reinforce their values, helping them feel understood and validated within their social and societal contexts [19]. This sense of validation fosters a greater sense of belonging and safety [20], which in turn enhances their self-esteem and emotional well-being [21].

Moving on to interactions with family, peers, and romantic partners reflects immediate social ecosystems. One of the core developmental tasks during adolescence is the formation and maintenance of meaningful interpersonal and social relationships. Social media plays a significant role in this process, serving as a platform where adolescents can share aspects of their personal lives and sustain social connections [22,23]. These interactions often begin within the family unit, where adolescents engage in intimate relationships and shared activities. It is common for adolescents to post content about family events or milestones, as these interactions form the foundation of their social networks, with 44% of US teens reporting that they post content about their family on social media [24]. As adolescents spend more time outside the home, particularly in school, they learn to navigate and build friendships with classmates and peers in environments separate from their families [25].

Then we will review institutional activities that support adolescent development by providing structure, fostering social connections, and creating opportunities for personal growth [26-30]. Participation in these activities also contributes to identity development by allowing youth to explore and express aspects of themselves within institutional frameworks; joining a basketball team, for example, offers the opportunity to learn the sport while also conferring the identity of a basketball player. Furthermore, these activities offer social opportunities for adolescents to gain in-group affiliation (eg, being a member of the junior varsity basketball team). Social media may extend this process by providing a space to publicly display and reinforce these identities [2,31].

The next topic being adolescent behaviors, specifically displayed on social media, including self-presentation, identity exploration, and sharing of personal achievements, reflects adolescents’ developmental imperative to construct a coherent self-concept and assert autonomy [11]. Guided by the psychosocial task of identity formation—a hallmark of adolescence [32]—teens use platforms to experiment with roles, express authenticity, and document milestones. For instance, self-presentation through curated selfies and photos, or candid mental health disclosures, allows adolescents to negotiate how they are perceived by others, while identity-related posts enable them to explore and affirm marginalized aspects of their identity [33,34]. Furthermore, these activities scaffold identity integration, bolster self-esteem, and enhance emotional regulation, processes that underpin socioemotional well-being [22].

Finally, in the digital age, social media provides a key avenue for maintaining peer relationships. Adolescents use social media not only to interact with friends but also to update their social status, share group activities, and express romantic interests [23]. For example, posting pictures or updates about time spent with friends or significant others allows adolescents to communicate and reinforce social bonds. Posting relational content online is crucial for adolescents’ socioemotional well-being [23]. Adolescence is a time for learning social norms and building identity, and sharing experiences with peers, family, or romantic partners on social media reinforces a sense of belonging. By publicly validating their social identities, adolescents strengthen their connections to important social groups [35]. These interactions also provide feedback and validation, which are essential for boosting self-esteem and feelings of belonging [36]. Moreover, posting relational content helps adolescents navigate social norms and satisfy their social needs. These fulfilled needs promote overall well-being by fostering secure attachments, emotional resilience, and a positive sense of self.

Building on the developmental foundations with the existing research on adolescents’ social media use, we introduce an adolescent-centered, cyberethnographically informed codebook that objectively captures adolescents’ SGC across 4 major platforms. This study was created to observe adolescent social media and their related well-being, with our ultimate goal being to create reliable, replicable categories to be used in future analyses about how posting specific content on social media influences adolescent well-being. This work will, in turn, allow parents or guardians and potentially policymakers to make more informed decisions about youth in relation to social media and then ultimately how these decisions will affect the well-being of the adolescents.

## Methods

### Creating the Codebook

**Figure 1.**
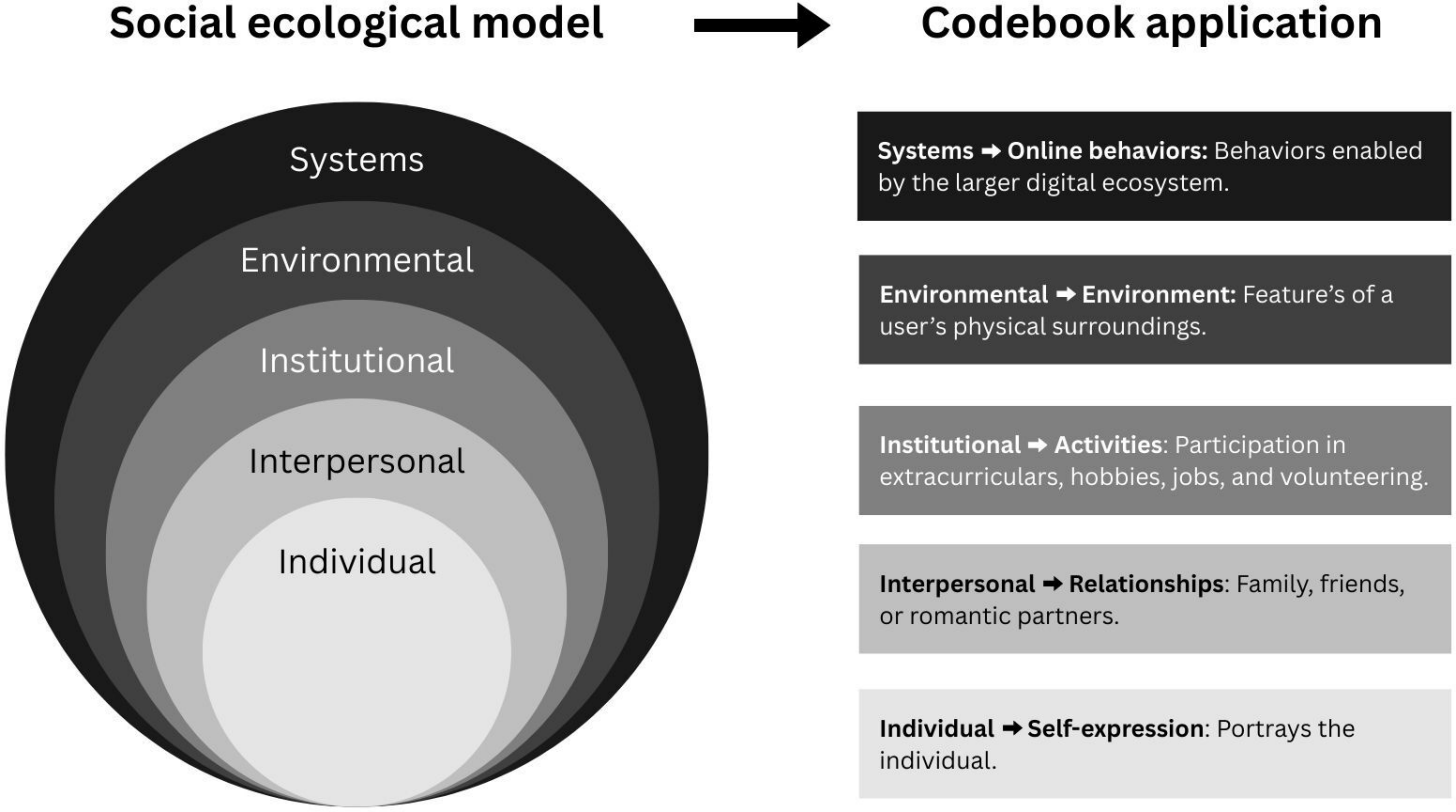
Social ecological model.

To categorize the themes in adolescents’ online posts, the research team created a detailed codebook aligned with Bronfenbrenner’s ecological systems model (Figure 1) [16]. Our codebook was designed to be applicable for posts made across 4 platforms, including Instagram, Facebook, X, and TikTok.

The initial version of this codebook was informed by the lab’s prior research on adolescent posting behaviors. Between 2019 and 2022, the lab conducted a study that directly observed 145 youth over 2 years through social media monitoring and dense cyberethnographic field notes [37]. These field notes were used to identify common patterns and themes across participants, informing the development of the codebook for the present study. The team identified 5 overarching themes in adolescents’ social media posts that reflect developmental processes unfolding across each ecological level (Figure 1), including self-expression, relationships, activities, environment, and online behavior. Through these 5 categories, the team drew from template analysis and framework analysis to create the concepts for the codes using the data that were previously collected by this study team [38]. Table 1 presents the complete codebook of themes, including subcodes and examples. Multimedia Appendix 1 includes sample posts and the applicable codes for each post.

**Table 1.** Codebook table.

Category and code	Definitions	Examples
Self-expression		
Selfie	Photograph or video of the participant, taken by the participant or someone else in the photo, while holding their phone.	Participant takes a front-camera selfie. Participant takes a mirror selfie with the back camera. Friend takes a selfie with the participant. Participant lip-syncs in a front-camera TikTok.
Portrait	Posed photo of the participant by themselves, taken by someone else, not engaged in an activity. Does not need to be formal or professional.	Participant’s formal senior photo. Participant poses in a solo birthday photo. Participant stands in front of a scenic view.
Self-promotion	Promoting personal work or ventures (eg, business, organization, event, social media) with an implied call to action (eg, to buy something, donate, visit a website, be recruited).	“Come to our school’s fundraiser concert Friday night.” “Donate to our Key Club fundraiser!” Participant’s sports highlight reel.
Positive mental health	Positively discussing mental well-being (eg, happiness, emotional growth, gaining skills).	Photo of participant’s artwork with caption: “So happy with my progress.” “Protecting my peace.”
Mental health challenges	Discussing mental health difficulties (eg, sadness, suicidal thoughts, self-harm, worsening depression).	“My parents hate me. They don’t understand.” “I feel so alone.” “Life sucks”
Politics	Engaging in political activism or discourse, social justice, and political critiques.	“Make sure you go vote!” Meme making fun of the president Infographic about a current political issue
Religion	Following the beliefs and rituals of a certain faith (eg, Attending church, participating in religious ceremonies, expressing devotion to God).	Photo of participant attending a religious service. “Be strong and courageous, Joshua 1:9”
LGBTQ+^a^	Poster directly mentions something about their gender identity or sexuality or experiences with LGBTQ+ topics.	“Non-binary and proud” Photos of participant at a Pride Parade with caption: “Happy Pride to me!”
Relationships		
Family	Parents, siblings, and extended family.	Photo of participant with siblings on vacation.
Peers	Peers, friends, classmates (ie, anybody that is not the participant or a family member).	Selfie of participant and friends getting food together.
Romantic relationships	Content referencing any stage or aspect of a romantic relationship, including the beginning (eg, starting to date), challenges or conflict (eg, breakups or relationship difficulties), positive experiences, expressions of being single, or general references to romantic involvement.	Photo of participant holding hands with someone, caption: “6 months with you <3.” Selfie of participant alone with caption: “Being single >>>.” TikTok of participant using trending audio about relationship red flags.
Activities		
Organized group	Participant engaged in an activity that is structured and pre-planned by a school or organization (eg, sports, theater, youth group), done in a group setting (eg, team, club), and is ostensibly supervised or directed by an adult mentor (eg, coach, instructor, teacher).	Photo of participant playing soccer with their club team. Group photo of participant with theater cast. Selfie of participant with school marching band. “Our debate team won first place at the state competition!”
Nonorganized group	Participant engaged in an individual activity performed within a group setting without formal organization, not including general socialization (hanging out with friends, getting dinner, etc).	Photo of participant fishing with a friend. Photo of participant’s artwork with caption: “I’m so proud of my progress!” Video of participant and friends lifting weights.
Self-directed	Participant engaged in leisure activity or interest pursued independently. Includes expressing and engaging with personal interests (eg, online fandoms, discourse about sports, music, movies, etc).	Photo of participant fishing alone. Photo of participant crocheting on a picnic blanket. Screenshot of Taylor Swift album with caption: “I’m the biggest Swiftie.”
Event	Attending an event (eg, school dance, concerts, sporting events). For events that have performances or competitions, this code should only be considered if the participant is a spectator (not an active participant).	Photo of participant with date at school homecoming dance. Photo of concert with caption: “Front row at the Taylor Swift concert in Chicago!” Photo of participant and family at Brewers baseball game.
Job and/or volunteering	Participants talk about or show themselves being employed or volunteering (eg, animal shelter and restaurant). Does NOT include household chores (laundry, family farm work etc).	Photo of participant in fast food uniform with caption: “Closing shift.” Photo of participant walking dogs at animal shelter.
Holiday	Observing or celebrating a particular holiday.	“Happy Diwali to all my Desi girls.” Photos from a Christmas parade.
Birthday	Birthday celebrations or happy birthday wishes.	Photos from participants’s 16th birthday party. Photo of birthday cake with candles lit.
Vacation and/or travel	Traveling, going on vacation, taking a trip, or visiting one’s cabin. Clear indication that the participant left their town of residence and visited a separate location (eg, captions may use the words “vacation,” “trip,” “break,” “travel,” etc).	Photo of participant on a beach with caption: “Spring Break 2025.” Video montage of cabin trip with outdoor scenery and campfire.
Environment		
School	Content explicitly created at school or contains topics related to academics or schooling.	Photo of participant and friends in school hallway. Photo of open laptop with caption: “Late night grind.”
Nature	Outdoor landscapes and plants. No human faces in the photo. Nature is the prominent or sole feature in the post.	Photo of mountain range taken during hike. Photo of sunset over a lake.
Animals	Posts that feature real animals, including pets, wild animals, zoo animals, or animals encountered outdoors.	Photo of participant’s dog lying on bed. Selfie with a goat at a petting zoo.
Food and/or beverage	Food or beverage is visible in the photo (eg, plates of food, cups, water bottles, etc).	Selfie of participant holding a Starbucks cup. Photo of Chick-fil-A bag and drink in car seat.
Online behavior		
Seeking online engagement	Seeking other users to engage with a post or a profile (eg, comments, likes, DMs) in a way that is directly or indirectly related to the participant.	“Comment 3 things you know about me.” “Repost if you think animal cruelty is wrong.” “What songs should I add to my playlist?”
Media	Content from mass media (TV, movies, video games, podcasts, music, professional sports, etc) and internet content (eg, memes, clips, etc). Content must be directly taken from source (not videos of a screen) but can be manipulated (eg, fancams, edits). Mere references to pop culture are not coded.	Screen-recorded TikTok reposting BTS fancam. Meme using SpongeBob still with humorous caption. Spotify screenshot of currently playing song.
Trend	Participant is engaging in a viral trend (eg, dances, lip-syncing, pranks, using CapCut templates, etc).	The participant performs a trending dance to popular audio. Video of participant doing the “choose your character” trend.

a LGBTQ+” lesbian, gay, bisexual, transgender, and queer and or questioning.

#### Self-Expression

This theme includes content focused on portraying the individual adolescent user, such as photos of themselves, posts promoting personal work or ventures, discussions of mental well-being, and expressions of personal identity. This theme aligns with the individual level of Bronfenbrenner’s nested ecological model, as it reflects the adolescent user’s personal characteristics, experiences, and self-concept.

#### Relationships

This theme includes posts featuring or discussing family, friends, or romantic partners, such as selfies with a friend, group photos, or family portraits. This theme reflects the “interpersonal” level of the nested ecological model, representing immediate social networks and reciprocal relationships that directly shape an individual’s development.

#### Activities

This theme includes content about extracurriculars, jobs, volunteering, and hobbies. This theme aligns largely with the “institutional” level of the ecological model, as these activities occur in structured settings, such as schools, workplaces, and community organizations. These institutional settings provide opportunities, resources, and social contexts that shape individual development.

#### Environment

This theme captures aspects of a person’s physical surroundings that may reflect or influence their physical, mental, or socioemotional well-being. This includes posts about individual items, such as pets or food, as well as broader settings, like schools or outdoor spaces, that may be linked to well-being. This theme corresponds to the “environmental” level of the ecological model, encompassing immediate, community, and cultural contexts that shape an individual’s experiences and development.

#### Online Behaviors

This theme captures actions performed specifically on the internet, including sharing mass media content and engaging with online trends. This category also includes online behaviors intended to increase engagement with a post or profile. In our adaptation of the ecological systems model, we conceptualize posting behavior as occurring within an overarching digital ecosystem; therefore, behaviors enabled by digital infrastructure and specific to online contexts align with the largest “systems” level of this model.

### Codebook Refinement and Back-Coding

These 5 major themes formed the preliminary codebook, which was first implemented in April 2023. Over the first year of coding, the codebook was applied to all collected data and continuously refined to address emerging patterns and discrepancies, ensuring that codes both accurately reflected the data and had strong interrater reliability. This refinement process was done through frequent team meetings to discuss and compare coding between posts reviewed by multiple coders, all of which active coders were required to attend, and if they could not make it, they would be required to watch the recording during their next shift. Adding a new code to the codebook consisted of having a new theme brought up during individual coding that didn’t fit in with any existing ones. Then the team would keep a list of all emergent themes, and if it was brought up more than one time by more than one person, the team would then need to reach a consensus to agree to add in that new code to the codebook. All updates were carefully documented in a shared document between the team to maintain a timeline of revisions for future reference. The codebook was finalized once no new emergent codes were identified by any coder during the coding process, indicating theoretical saturation. After multiple iterations, the codebook was finalized in June 2024. Since the codebook has been finalized, no additional codes have been created; however, if a subject does come up that appears to not fit into a category, the team will meet to discuss what the best codes are that do fit that. All subsequently collected data were coded using this final version.

**Figure 2.**
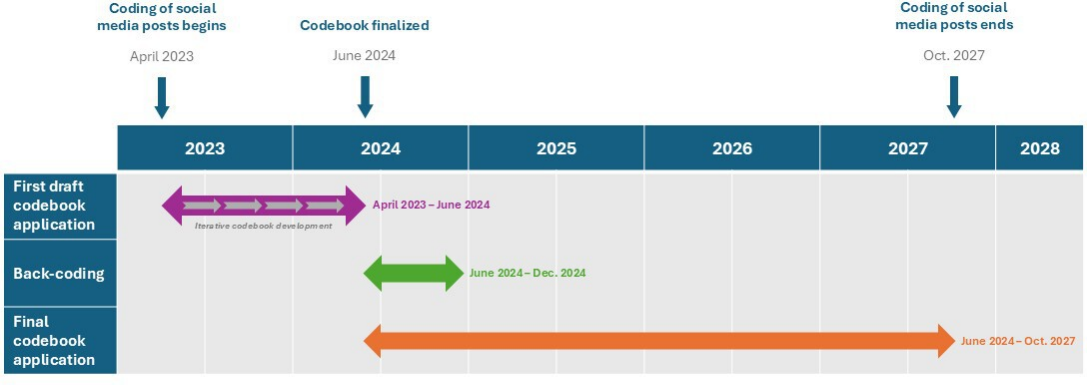
Codebook timeline.

To ensure consistency across the dataset, all data coded before the codebook was finalized (April 2023-June 2024) was systematically back-coded according to the final codebook. Using the documentation of all codebook changes, the study team created a detailed guide for coders that included a comprehensive list of changes and instructions on how to address these changes while back-coding. The back-coding process, which ran from July to December 2024, took place alongside ongoing data collection. See Figure 2 for a timeline of codebook development and application.

### Data Collection Tools

Research Electronic Data Capture (REDCap; Vanderbilt University) is our primary data collection tool, chosen for being accessible, flexible, and secure. REDCap is a free, web-based, open-source platform that is Health Insurance Portability and Accountability Act (HIPAA)–compliant and hosted on our institution’s servers. REDCap allows research teams to create multiple customizable forms for data collection and coding tracking and provides storage for the repository of coding data. Although we do recognize that there are other tools that could be used for this project, REDCap has a special team at the University of Wisconsin–Madison that is ready and able to help with issues or questions on programming. This has been incredibly helpful for project creation and execution. This team had previously used Qualtrics (Qualtrics International Inc) for a different project and found that the data collection was fairly easy; however, when it came to data exportation and analysis, this platform was not as user-friendly as REDCap.

To view participants’ social media accounts, we created multiple study-specific profiles on each platform (Facebook, Instagram, TikTok, and X). Study accounts use fictitious names and photos designed to resemble typical user profiles and are periodically updated with posts. This approach reduces the likelihood of being flagged as bots or inadvertently revealing participants’ involvement in the study. Multiple profiles were also created in advance to serve as backups in case of accessibility issues. With parental consent and minor assent, we request to follow participants’ accounts on each platform using the study profiles during enrollment and conduct six-month follow-ups to verify that the information remains current. All research team members use the same accounts for data collection.

### Unit of Analysis and Contextual Coding Procedures

To ensure consistency in coding, our team first defined the unit of analysis—the portion of a social media post that would be coded. In this study, the unit of analysis is the entire self-generated post, including its media (eg, photos and videos), audio, and captions. Because our focus is on content created by participants, comments are not thematically coded. However, on platforms where engagement is visible, coders record the number of comments the participant writes, reacts to, or responds to, providing additional insight into how participants interact with their own posts. Each platform varied in the information that is to be collected. From Instagram having regular posts to reels to highlights to stories, we created a variety of forms to encapsulate this information in a meaningful way for this study. Instructions for the coding procedures can be found in Multimedia Appendix 2.

Our team uses a holistic approach, using participants’ shared social media accounts and other publicly available data to properly code each post. When needed, coders are instructed to gather contextual information from any of the participant’s social media accounts followed by the study team. Coders may also reference other public information, such as usernames of tagged individuals, to support coding determinations. For example, if a post features the participant alongside another similarly aged individual, it might be unclear whether this person is a friend or a family member. By reviewing the participant’s social media history, a coder may observe that this individual only appears in family-related posts. Additionally, a publicly visible username may reveal a shared last name, suggesting a familial relationship and allowing for more precise coding.

Coders are encouraged to explore social media platforms and the broader internet to contextualize posts to identify online trends, interpret online vernacular, or understand niche or unknown references. For instance, if a participant mentioned going to an “LARP event,” a quick Google search could reveal that “LARP” stands for “live-action role play.” Coders are also instructed to use search tools and platform-specific features—for example, TikTok’s shared public audio function—to identify trends or clarify content. However, coders are strictly prohibited from using personally identifiable information in any online searches and are instructed not to collect or record data from accounts that have not explicitly consented to participate in the study.

### Study Design

This project and team are a part of a larger longitudinal observational study that is conducted over the course of 24 months, using a mixed methods study design consisting of both qualitative and quantitative data collection [39]. There are 3 principal investigators (PIs) who man individual teams varying in size from 5 to 20 research staff at any time, which are made up of undergraduate students, graduate students, postdoctoral students, and professionals.

### Well-Being Data

The larger longitudinal observation study is collecting well-being data at different time points throughout the study. Some of the well-being metrics being collected include Media and Technology Usage and Attitudes Scale (MTUA), General Anxiety Disorder 7-item (GAD-7), Emotional Responses to Social Media Experiences Measure (ERSMEM), 8-item Patient Health Questionnaire (PHQ-8), and more [40-43]. These metrics are collected at 3 time points over the 24 months and will be used to connect to the participants’ social media data at different time points throughout the study.

### Participants

The participants in this study are adolescents aged 13‐15 years from the state of Wisconsin who have at least one active social media profile on Facebook, X, Instagram, and/or TikTok. These participants were sourced from local events in Wisconsin such as the state fair, Wisconsin Science Festival, and more, where team members from the study talked to potential participants and their parent or guardian about the study and enrollment. Our team used the census data from 2020 for the state of Wisconsin for our targeted enrollment demographics. Each participant, when joining the study, shares their social media platforms and is followed by the study-specific profiles mentioned above. This team then goes into each participant’s profile every month to code and collect the data.

### Coding Team

Our team began coding study data in April 2023, with the initial coding conducted by research staff with backgrounds in adolescent medicine, public health, and psychology. Beginning in September 2023, undergraduate student research assistants were hired to assist with data collection. The size of the coding team has fluctuated over the course of the study based on workload and coder availability. As of the time of writing, 28 coders have contributed to data collection, each participating for at least 4 months. The standing study team for this project is built of PI (ES), 5 research staff (KB, LB, TF, KK, and CMC), and alternating undergraduate student research assistants.

### Coder Training

First, our study team developed a comprehensive coding guide to support coders in both learning the coding process and referencing updated procedures throughout the project. The guide was designed to be highly visual and easy to navigate and included screenshots of social media and data collection platforms, step-by-step coding instructions, and troubleshooting strategies. Any updates to the coding process are immediately reflected in the guide, which is used as the definitive reference to ensure consistency across coders and throughout the data collection process.

Coders begin training by meeting one-on-one with the lead study team member who reviews the coding process and explains each code in detail. Coders then practice applying this codebook to a sample social media post with the assistance of their supervisor to assess their understanding. Using the coding guide and codebook, coders then complete a training exercise by coding 24 preselected social media posts (6 from each platform). Upon completion, a REDCap report identifies agreements and discrepancies between their coding and the answer key, created by their supervisor. Each coder then meets with their supervisor to review results post-by-post, discussing agreements, discrepancies, and refining their understanding of the codebook.

Coders who achieve at least 80% agreement with the master coder begin official data collection under close supervision to address any lingering questions. While most coders reach this threshold after the first round, those who do not repeat the process with a new set of posts. As of writing, no coders have required more than two rounds, though a third set of posts was prepared as a contingency.

### Coding

**Figure 3.**
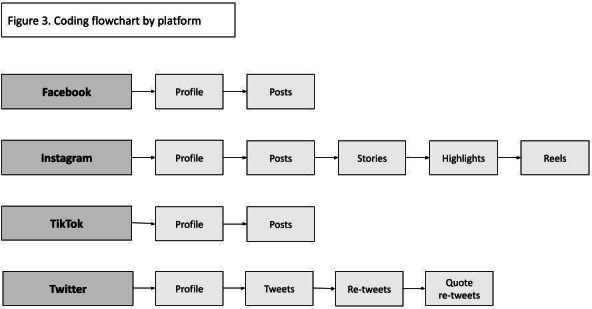
Coding flowchart by platform.

Coding is conducted monthly for each participant, with each 30-day interval following enrollment representing an observation period. Participants are followed for 24 observation periods as part of a larger longitudinal, multimethod study [39]. Content is coded from participants’ Facebook, Instagram, TikTok, and X accounts (Figure 3). Something to note is that each post can be assigned more than one thematic code, as many posts are complex and include a variety of subjects.

### Assigning Coding

We assign coding using REDCap’s Data Resolution Workflow, a feature typically intended for formally flagging data issues (eg, missing, incorrect, or invalid values). This workflow enables users to open a query attached to a specific data field, assign it to another user, and track its resolution. Although not designed for this purpose, our team adapted the feature to assign coding tasks to individual team members.

Coding is organized by “participant-months,” where each unit represents one month of coding for a single participant. At enrollment, each participant’s full coding schedule was programmed into REDCap for the duration of their study participation. When a participant-month became due, it appeared on REDCap’s calendar.

To make assignments, the data manager reviews the calendar daily to identify participant-months scheduled for coding. For each assignment, the manager opened a query in a form on the participant’s profile, specifying the participant-month to be completed. Coders accessed their assignments through the data resolution dashboard by filtering queries by their username.

### Quality Control

Given the volume of data, number of coders, and duration of the study, it was essential to establish rigorous procedures to ensure data integrity. To support accuracy and consistency, our team implemented annual interrater reliability checks, double-coding procedures, and weekly alignment meetings.

#### Annual Interrater Reliability Checks

Each year, we randomly selected 6 participant posts from each social media platform (Facebook, Instagram, TikTok, and X), yielding a total of 24 posts. All active coders independently coded each post, and the results were compiled and assessed using the Gwet First-Order agreement coefficient (AC1), which is well-suited for evaluating interrater reliability with binary outcomes and multiple raters [44]. A passing threshold was set at >80%. If this threshold was not met, the team completed a second round of interrater coding and recalculated the AC1 score. These checks were typically conducted each spring and repeated annually for the duration of the study.

#### Weekly Team Meetings

Our team holds weekly meetings to keep coders aligned on the codebook and informed about any protocol or administrative updates. Each week, one coder shares a social media post with the team, typically one that is challenging to code or raises questions about applying specific codes. The coder presents the post along with their coding decisions, interpretation, and any questions about applying the codebook to unique features of the content. Team members then share their own coding determinations, and the group discusses discrepancies until reaching consensus. This practice helps the coding team refine their understanding of how codes are applied and highlights areas where additional clarification may be needed in the coding guide.

### Concerning Content Protocol

Because 2 of our PIs are mandated reporters, our study team has both a legal and ethical obligation to comply with mandated reporting requirements [45]. To support this, we developed a standard operating procedure for how to identify and report content on participants’ social media profiles that might meet reporting criteria. Our standard operating procedure ensures that any reportable content is identified and escalated to the investigators in a timely and responsible manner.

For the purposes of this study, concerning content is defined as posts that referenced suicidality or self-harm, sexual or physical abuse, or threats of harm or violence toward others. Such content can appear in the participant’s own words or in reposted material that suggests the participant may be at personal risk of harm. A made-up example of this would be a participant posting “I’m going to school with a gun tomorrow,” which would require prompt action from our team. Notably, concerning content does not include all illegal or risky behaviors, which do not always meet the threshold for mandated reporting [46].

### Ethical Considerations

Institutional Review Board approval was obtained from Application Review for Research Oversight at Wisconsin (ARROW) under Institutional Review Board number 2022‐1280. Informed consent is given through a survey through REDCap that each participant’s parent or guardian must sign before being enrolled in the study. All participant and related data are stored in the secure University of Wisconsin-Madison REDCap and are deidentified when exported for analysis. Participants can receive a total of up to US $300 for completing all study-related activities.

## Results

### Overview

This study was funded in 2022 and began data collection in early 2023, with the codebook still in development, with the study concluding in 2027; however, there is a possibility of continuation. Figure 2 outlines the timeline from beginning to the expected end of the study. There are 369 participants at the time of this writing, with 336 actively enrolled and 33 withdrawn.

### Coding Progress

**Figure 4.**
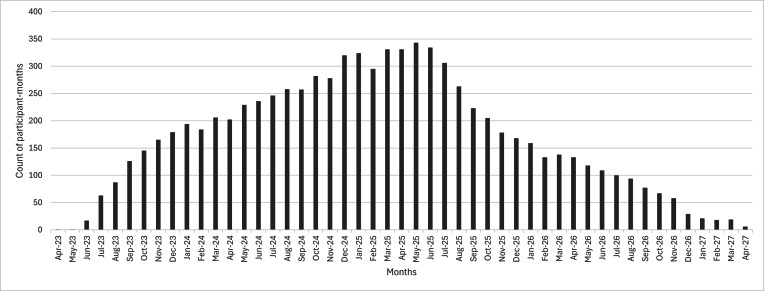
Frequency of participant-months per month.

Figure 4 illustrates the trend in participant-months requiring coding. The workload peaks around the midpoint of the study (mid-2025) and then declines as participants reach the end of the 2-year enrollment period. Month-to-month variation reflects the staggered and fluctuating nature of participant enrollment.

### Quality Control Measures

Annual interrater reliability assessments produce 5 AC1 scores, one for each platform (Facebook, Instagram, TikTok, and X) and one overall score. Year 1 results, shown in Table 2, indicate that Instagram had the highest agreement (0.89) and X the lowest (0.87), with all scores exceeding the 0.80 acceptability threshold. In Year 2 to date, X shows the highest agreement (0.96), while Facebook has the lowest (0.95).

**Table 2.** Interrater reliability agreement scores.

Years	Interrater reliability agreement scores				
	Facebook	Instagram	TikTok	Twitter	Overall
Year 1	0.89	0.89	0.88	0.87	0.88
Year 2	0.95	0.96	0.96	0.96	0.96

## Discussion

### Strengths and Unique Contributions

#### Using Human Coders

Using research staff and hiring student research assistants for social media data collection has been a cornerstone of our methodology. Human coders add essential cultural and contextual understanding to content coding, particularly when interpreting the highly complex and dynamic nature of adolescent social media content. Human coders can be better equipped than automated tools (such as artificial intelligence [AI] systems or data scraping algorithms) in understanding context, recognizing tone, subtext, peer norms, and internet colloquialisms. These nuances are central to online communication and often carry significant meaning; even the most advanced automated tools can often remain unreliable in detecting them accurately. However, there is a time and a place where AI may have a value that is considered to be more useful than a human coder, such as for large-scale data collection that would not be feasible for a human coder to complete.

Human coders, our lab’s student research assistants, carry out much of the coding and play a central role in data collection. Aged 18-22 years, they are close in age to our adolescent study participants, aged 13-17 years. Both groups belong to the same generation of “digital natives,” young people who have grown up during the age of digital technology and are intimately familiar with social media. This generational proximity enhances the coders’ ability to recognize platform-specific norms, contextual nuances, and forms of expression that might otherwise be overlooked, supporting more accurate and culturally informed data collection and interpretation.

Human coders are also highly adaptive. Given the nearly limitless variety of social media posts adolescents create, the tools used to collect and interpret this data must be equally flexible. Human coders can respond to unexpected or ambiguous content in ways that preprogrammed systems cannot, especially in a research context where interpretive accuracy is critical. This approach also enables real-time, context-sensitive interpretation. Unlike retrospective analysis, which may fail to account for fleeting internet trends or evolving slang, human coders can engage with content as it emerges, an essential feature when working in rapidly changing digital environments.

Unlike automated data scraping (ie, using machine learning and other big data approaches), which is often limited by technical barriers and platform-specific API restrictions, human coders can access and analyze content across platforms without relying on unstable or restricted infrastructure. This is particularly advantageous in longitudinal studies where interruptions to data access could compromise the dataset. Human coders are also able to synthesize multimodal content, including video, audio, text, and comments, which often carry interdependent and context-specific meaning. While automated tools may struggle to parse and integrate these multiple layers of information, human coders can evaluate them holistically, adding coherence and nuance to coding decisions.

Human coding also ensures greater transparency and traceability. Unlike the opaque decision-making processes of AI-driven models, human coding decisions can be documented, discussed, revisited, and replicated. Weekly team meetings allow coders to refine their approach, address inconsistencies, and ensure the integrity of the dataset through shared understanding and collaborative troubleshooting. Ethically, human coders provide a more secure and accountable approach to data handling. Our method does not rely on third-party tools, reducing the risk of privacy breaches. Additionally, coders can manually flag content of concern in a thoughtful and timely manner, an important safeguard in studies that involve mandated reporters.

#### Cyberethnography

From a methodological standpoint, the foundations of our codebook in cyberethnography bring unique advantages to social media research. Foundationally, ethnography in general is meant to provide a picture of daily life, and for adolescents, daily life occurs online. Given the concordance of social media displays and adolescent offline behavior [47,48], the online space represents an important natural environment for observation of adolescent social behavior. Traditional measures of social media experiences, such as survey measures, ecological momentary assessment, and interviews, rely on self-report. The aforementioned methods have the strength of the inclusion of youth voices and their perspectives on social media, particularly in the presence of rigorous data collection instruments. The method described in this manuscript, cyberethnography, allows the research team to objectively observe adolescents’ social media activity to further analyze what each participant is trying to portray through their SGC. Whereas, if the team were to rely upon just interviews, the information could fall prey to bias from individuals reporting on their own social media activity rather than benefiting from objective observation through cyberethnography.

#### Large Number of Posts and Longitudinal Approach

With a considerable sample size of social media posts such as ours, we are able to add additional dimensions to traditional cyberethnographic analysis. While sample size is an area of much debate in qualitative research [49], arguably with a group of hundreds of participants and thousands of posts, theoretical saturation is easily reached. We are then able to scale up our codebook to create quantitative variables that can be used as longitudinal predictors of outcomes of interest, for example, socioemotional well-being indicators. With participants enrolled for 2 years with multiple data points, our research is responsive to calls for longitudinal research to determine bidirectional relationships between social media experiences and socioemotional well-being [50].

#### Informed Data Collection

Another unique advantage of this methodology is its emphasis on ethical data collection that is completed internally within the research team. We very carefully collect informed consent and assent for all participants. This in itself is unique for the collection of social media data at the level of detail we use and has been met with both hesitation and trust. We note that social media companies collect much more data than this project, with very little transparency. With long, complex terms of service documentation that users must accept to use social media platforms, the “consent” that users provide for this data collection is questionably “informed.” We believe that an informed consent model for social media data collection ultimately fosters trust in researchers through transparency about exactly what is collected and why [51]. That being said, the use of human researchers to observe social media data may cause trepidation in adolescents and their parents. Future research could explore further with adolescents and their parents such concerns and possible mitigation strategies.

In addition, human review raises ethical concerns as to sensitive content; if a coder observes content depicting illegal behavior, self-harm, or abuse, what is the duty of care to address this? In our study, we are guided by the principles of mandated reporting, the same reporting structure that guides the physician members of our investigative team in clinical settings. This approach is particularly useful in a cohort study in which we have regular interactions with participants and prioritize safety and confidentiality [52].

#### REDCap

REDCap was chosen as the data repository for this project because it met HIPAA compliance and supported our diverse data collection needs. REDCap allowed for all team members to be able to be added to the specific projects and work simultaneously on data collection. The use of REDCap also enabled the team to import the participants from a spreadsheet. The functionality of the calendar and scheduling then became useful for creating the 24 months of observation periods. The use of the reports and exports also allowed the team to perform quality control checks and data analysis for project tracking and manuscript writing. All of these processes and actions could then be followed through the use of the dashboards via REDCap, which allowed for the data and tracking to be observed. REDCap is also fully supported by our academic institution, which has increased efficiency in troubleshooting any issues that have arisen. With an on-campus data manager, our research team could rely upon timely responses from the REDCap support team at our institution.

### Lessons Learned

Applying this method included important considerations, including the team, participant social media use, and policies impacting social media use.

#### Research Team Training and Fluctuations

The research team changed over the course of the codebook’s development, training, and application. While team turnover may lead to more generalizable codes, the changes also created rationale and interpretation gaps. The team maintained interpretable codes by writing clear definitions, using a single document to house definitions and decisions, hosting regular staff meetings to re-emphasize code applications, and adjusting definitions or adding codes as the team collected more data. The high interrater reliability scores highlight the strength of the strategy. Future applications of this method should ensure written definitions of codes in one place, flexibility in code definitions, and regular communication with the coding team.

#### Researcher Reflexivity

Any study with a qualitative component carries concerns for “blind spots” in interpretation based on the lived experiences and positionality of the individuals conducting the research and designing the codebook. Our team has attempted to mitigate potential bias by active inclusion of individuals of diverse age, race and/or ethnicity, sexuality, gender identity, and discipline. Furthermore, frequent meetings during both refinement and implementation of the codebook allow for the contribution of new voices as the study goes on.

#### Account Access Challenges

One of the challenges that was fairly constant over the course of the study was with access to participant social media accounts. Participants adjusted accounts throughout the study, such as changing usernames, unfollowing our research accounts, adding new accounts, deleting current accounts, and having accounts suspended for various reasons. Troubleshooting changes to account access was a continual issue with the risk of nonaccess resulting in missing data for that time point. To address this, a point person was assigned to handle all issues with username access as they arose, which allowed research staff to continue to code available profiles and maintain coding progress. All accounts with access issues were assigned and coded once the issue was resolved.

Another issue the research team faced was the terms and conditions policies related to each social media platform. Having a shared research team profile for each social media platform is an atypical use case but is necessary for maximal access to participant profiles in the absence of collaboration with platforms using their APIs. Having multiple researchers accessing the same social media account and scrolling back through posts for each participant every month carried risk for research group accounts to be flagged as spam or bot accounts. After being flagged, our research team is not able to follow or add any new participants to that account. Once the research account is flagged as spam, there is little possibility for restoration, a common experience for users of these platforms. Over the course of the study, 3 of our research accounts were flagged as spam, and we addressed this by creating new profiles to follow new research participants. Flexibility and quick troubleshooting were needed to make sure all participant accounts were accessible throughout the study. To maintain security and accessibility of multiple coders accessing accounts at the same time, using the University VPN and deleting saved cache files or saved passwords on computers, as well as using different browser platforms to access different profile accounts. By adopting these policies, the team was able to maintain the four profiles without other flagging issues occurring.

#### Federal Platform Regulations

Accessing certain social media platforms among evolving federal regulations—particularly regarding TikTok—was another unforeseen issue the research team had to troubleshoot. As a public institution, accessing special permissions for research was sought for our study to have access to TikTok on university-owned computers. Further, the passing of HR 7521, the “Protecting Americans from Foreign Adversary Controlled Applications Act” (2024), would delay or terminate our data collection on this platform. The bill prohibits the distribution, maintenance, and updating of certain foreign-owned applications. As a foreign-owned company, TikTok is at risk of being removed from United States app stores and web hosting services. Enforcement of the bill would significantly harm our understanding of self-generated content in adolescents, especially since an increasing percentage of enrolled participants are users with multiple accounts on TikTok. Their active use demonstrates the popularity and importance of this platform to our adolescent participants. Ultimately, the ban only went into effect for less than one day before being temporarily delayed by the Trump administration, interrupting data collection for only a short period [53]. As of writing, the ban deadline has been extended 3 times via executive order [54]. The future of TikTok remains uncertain, and enforcement of the ban threatens to disrupt future data collection efforts.

#### Platform Variability and Change

The team needed to adjust for the distinct functionalities within social media platforms. Adolescents share self-generated content differently between social media platforms. For example, Facebook and X tend to be associated with text-based content, whereas Instagram and TikTok are more visual (ie, photos and short-form videos). Further, users can share content on Instagram in more spaces (eg, Stories, Highlights, the “grid”) compared to other social media platforms. Previous research explores how different functionalities may hint at different user motivations [55,56]. Therefore, this codebook applied to a wide range of SGCs. To navigate platform differences, the team used tailored REDCap forms by platform to ensure all possible self-generated content was captured.

We also needed to adapt to changes within platforms. For example, in February 2023, TikTok added “Reposts,” a functionality that allows content created by one user to be reshared by a second user. Unlike Facebook and Instagram, TikTok reposts only show the likes and comments from the original post and do not include timestamps of when the content was reposted. Additionally, participants who reposted content on TikTok were likely to repost a heavy volume within the observation period, challenging the team’s ability to effectively code these posts separately. Therefore, the team needed to make 2 separate determinations: first, to what extent are TikTok reposts self-generated content? Second, how should TikTok reposts be incorporated?

To navigate this change, we relied on multiple key strengths, including routine, open communication with team members, the flexibility of the method, and the versatility of REDCap. We first discussed the feasibility and importance of coding reposts with the coding team. Through this discussion, we decided that all reposts are important to capture, but that theming each post individually is not feasible due to the volume of reposts. Therefore, we adapted our method by counting the number of reposts within an observation period and summarizing the content generally. We also added a section in REDCap to mark the last repost shared in the previous observation period to more accurately count the number of reposts in the next observation period.

Other less notable examples of platform changes include Facebook adding Stories, Instagram increasing their cap on the number of photos in a post, and platforms hiding the number of likes and views on participant posts. In all cases, the coding guide was changed, and the coding team was notified. Social media platforms are distinct, and their evolution is inevitable. Researchers who apply this method should prepare to navigate platform differences throughout the course of their project and lean on the expertise and communication of their team to navigate challenges.

#### Other Limitations

More notable restrictions that researchers may face include API restrictions, which we have encountered in regard to a different project, forcing the team to find alternative ways to collect the needed data. This would also vary across different countries in the world as well, since each country has differing laws on data access and collection.

### Importance

The implications of our methodology are many. First, the provision of granular, researcher-observed data regarding self-generated social media content not only densely phenotypes adolescent online behavior but also allows for identification of more precise online experiences that may have positive or negative impacts on a multitude of physical and mental outcomes. The impact of social media on the health and well-being of adolescents is of concern to adolescents, parents, policymakers, and the public [53,54]. Objective identification of risky and healthy online behaviors can aid in future development of interventions, from parental, adolescent, and clinician-based education to detection and amplification at the design level. The latter, however, would require close partnership and transparency between researchers and social media companies, which may need to be enforced at a policy level.

Across other research studies, there have been a variety of different approaches taken to code adolescent social media. One study focused on coding race- and ethnicity-related content (text and images) in Facebook public posts through a hybrid inductive-deductive approach [57]. Which is similar to our study in that we used a hybrid approach but focused mostly on psychosocial and/or well-being–related content across 4 platforms, and we also included private posts as well. Another study focused on health-risk–related content, which also followed a theoretical theory (deductive approach) first followed by an inductive approach, so it was very similar to our approach that we used [58]. As for content coding of profiles versus just posts, one other paper coded users’ profiles and then further developed a codebook for sexualized behaviors on Facebook, which draws to our study as well because we not only collected posting data but also coded profiles for each account per platform [59]. Finally, some of our team’s previous social media research work includes creating a codebook that was designed for YouTube video content, which ultimately helped us to have an idea of how to navigate creating a codebook for social media data collection [60].

This project offers both methodological and theoretical advances to adolescent social media research. First, it adds to the field’s previous evidence using self-reported screen time or broad “active–passive” classifications by objectively analyzing the actual posts adolescents create. Each participant’s content (video, text, photo, etc) is captured across multiple platforms (TikTok, Instagram, Facebook, and X) over a 2-year span, providing a granular, longitudinal view of their digital lives that is unattainable with single-platform or cross-sectional designs. Second, the study introduces an adolescent-centered cyberethnographic coding framework. The codebook was applied by rigorously trained coders, ensuring high interrater reliability while eliminating recall bias. This participatory approach yields a faithful record of what adolescents choose to share online. Third, the coding scheme is grounded in Bronfenbrenner’s ecological model, capturing posting themes at 5 nested levels, including individual, interpersonal, institutional, environmental, and systems. By aligning observable social media behavior with a well-established developmental theory, the study links online self-generated content to the broader contextual layers that shape adolescent well-being. Together, these innovations furnish a holistic portrait of adolescents’ online expression and lay the groundwork for future research examining how specific posting patterns relate to socioemotional outcomes.

### Planned Analysis

Our study team is already in the process of running initial analysis on the descriptives of these data that we are collecting. Beyond a descriptive analysis, we have been working on cross-analyzing the engagement of the participants’ peers on their social media posts in relation to the participants’ overall well-being from different time points throughout the study. These are just a few examples of how this data will be used; however, overall, we are planning to run many different types of analyses on the well-being data that are being collected and the SGC that is also being coded by this team. We will be able to show how one impacts the other and vice versa, which is information that will hopefully help parents or guardians and policymakers.

### Conclusion

This formative research approach that was taken with our lab has allowed for us to have insight and collect data that will give a new perspective on social media and adolescents. This, in turn, will help our research team to further show the connection between social media and socioemotional well-being. Specifically, this team plans to write a descriptive paper on the data that is being collected. We then plan to work with the other teams on the bigger project to connect participants’ well-being with the social media data that we have collected. This team works with undergraduate and graduate students, and postdoc and research professionals across the University of Wisconsin–Madison, who will also use the data that we have collected to answer differing research questions. This approach will aid future studies in project creation and execution on studies similar in nature. The foundation that has been laid out by this team should be a guidance to further the research on the effects of social media on adolescents and within other age groups as well. This research strategy could also be extended to conducting research in different ethnically diverse populations across the globe. This research is critical to understanding the well-being of individuals who participate in social media use, especially with the ever-changing nature of social media. This team will continue to publish research outcomes from the data collected from this methodological approach and will carry on studying the well-being of adolescents and their interactions with social media.

## Supplementary material

10.2196/84461 Multimedia Appendix 1 Sample social media posts with applicable codes.

10.2196/84461 Multimedia Appendix 2 Coding guide.

## Abbreviations

AC1: Gwet First-Order agreement coefficient
AI: artificial intelligence
API: Application Programming Interface
ARROW: Application Review for Research Oversight at Wisconsin
ERSMEM: Emotional Responses to Social Media Experiences Measure
GAD-7: General Anxiety Disorder 7-item
HIPAA: Health Insurance Portability and Accountability Act
MTUA: Media and Technology Usage and Attitudes scale
PHQ-8: 8-item Patient Health Questionnaire
PI: principal investigator
REDCap: Research Electronic Data Capture
SGC: self-generated content
