# Supplementary figures and images for: Directly Observing and Characterizing Adolescents' Self-Generated Social Media Posts: Protocol for Creation and Implementation of a Cyberethnography Informed Codebook
Source: JMIR Res Protoc. 2026 Mar 31;15:e84461. doi: 10.2196/84461 (PMC13037698; doi:10.2196/84461)

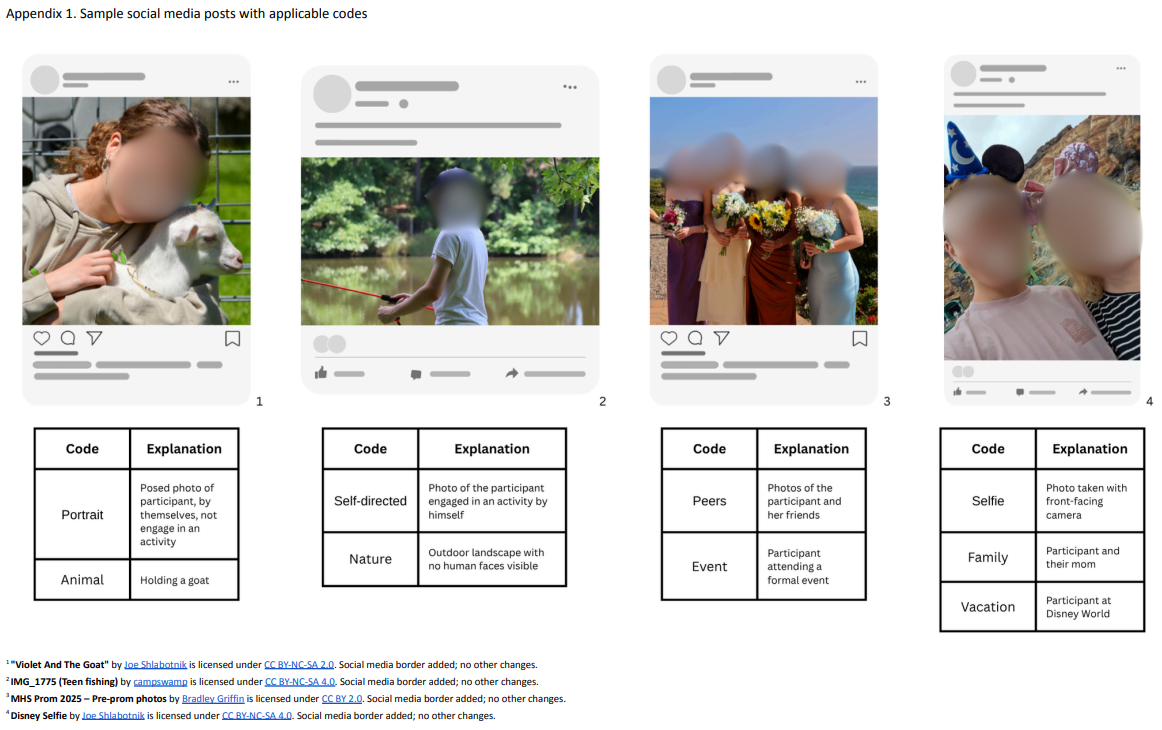

Supplement: Multimedia Appendix 1 [file resprot-v15-e84461-s001.png]
