# Supplementary material for: Directly Observing and Characterizing Adolescents' Self-Generated Social Media Posts: Protocol for Creation and Implementation of a Cyberethnography Informed Codebook
Source: JMIR Res Protoc. 2026 Mar 31;15:e84461. doi: 10.2196/84461 (PMC13037698; doi:10.2196/84461)
Supplement: Multimedia Appendix 2 [file resprot-v15-e84461-s002.pdf]

Step-by-Step Coding Guide

Coding Process: At a Glance

1. Check assignments
2. Identify the participants’ social media accounts
3. Open the participants’ social media accounts using the team profiles
4. Complete coding forms for each profile and post for all accounts owned by the participant
5. Mark assignment as “Complete”

Connect to the VPN

Always use GlobalProtect VPN when coding to protect participants and to make sure our social media accounts don't get flagged for bot behavior.

1. Search for “GlobalProtect” on the bottom left search bar or find the globe icon on the bottom right
2. Click “Connect”
3. Sign in using your NetID and password
4. Confirm your sign-in using Duo authentication

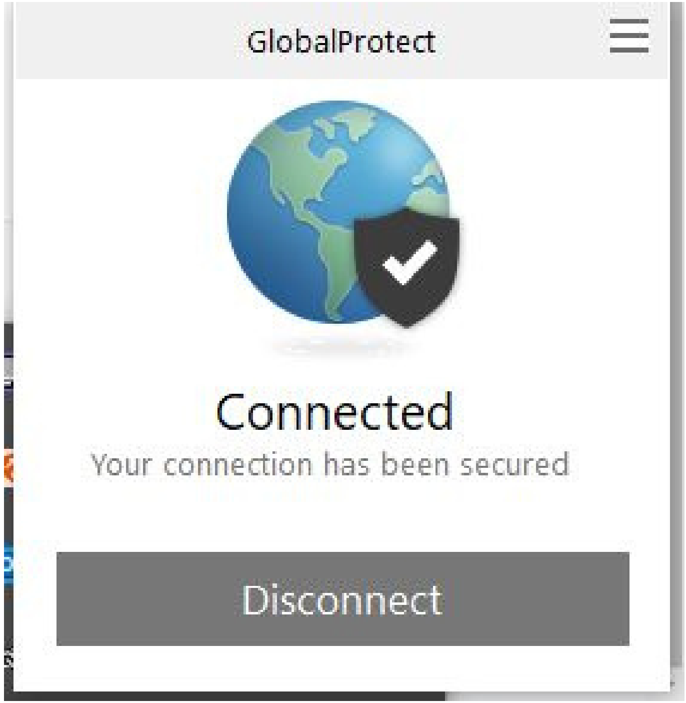

Step 1: Check assignments

1. Open the REDCap project.
2. Go to project bookmarks on the side menu bar.
3. Select your name to see the list of participants you've been assigned.
4. Select the **Record ID** to go to the ppt's record to start coding.

Project Bookmarks

- [redacted]'s Assigned
- [redacted]'s Assigned
- [redacted]'s Assigned
- [redacted]'s Assigned
- [redacted]'s Assigned
- [redacted]'s Assigned
- [redacted]'s Assigned

Month assigned *Assignment details*

Click button to view data query	Record (Sorted by DAG)	Data Quality rule and/or Field	User Assigned	Days Open	First Update
1 comment	270 Info	Field: **m7_c1** (Month 7 - Coder 1)	[redacted]	10.1	[redacted] (02/19/2024 12:44pm): "ASSIGNMENT: Monday (2/19)"
1 comment	308 Info	Field: **m6_c1** (Month 6 - Coder 1)	[redacted]	20.9	[redacted] (02/08/2024 5:18pm): "ASSIGNMENT: Friday (2/9)"
1 comment	485 Info	Field: **m3_c1** (Month 3 - Coder 1)	[redacted]	16	[redacted] (02/13/2024 2:55pm): "ASSIGNMENT: Thursday (21/25)"

Rec ID *Month assigned* *Assignment details*

Record (Sorted by DAG)	Data Quality rule and/or Field	User Assigned	Days Open	First Update
270 Info	Field: **m7_c1** (Month 7 - Coder 1)	[illegible]	10.1	[illegible] (02/19/2024 12:44pm): "ASSIGNMENT: Monday (2/19)"
308 Info	Field: **m6_c1** (Month 6 - Coder 1)	[illegible]	20.9	[illegible] (02/08/2024 5:18pm): "ASSIGNMENT: Friday (2/9)"
485 Info	Field: **m3_c1** (Month 3 - Coder 1)	[illegible]	16	[illegible] (02/13/2024 2:55pm): "ASSIGNMENT: Thursday (21/25)"

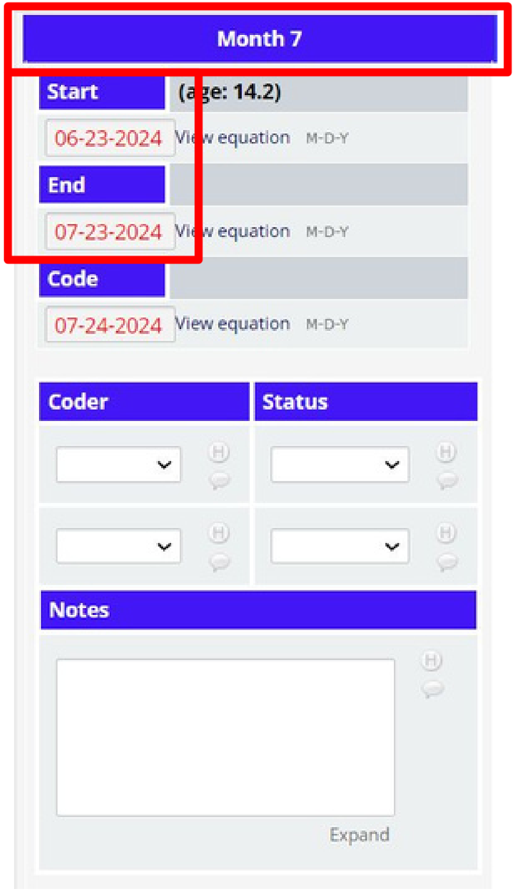

1. Identify the month assigned for coding.
2. Select the Record ID to go to the ppt's record.
3. Go to "Info" form and find the month assigned.
4. Identify the start and end date of the observation period for the month assigned.

   Note: Participants areenrolledon a rolling basis. Therefore, the start and end dates of each month vary by participant.

Coding Assignments

- Participant profiles and posts are coded **one month at a time.**
- The month in which coding is completed is referred to as an "observation period" or "OP."
- Code posts they were made **on and between the start and end dates** of the month assigned.
- Coding is assigned to you by [staff]

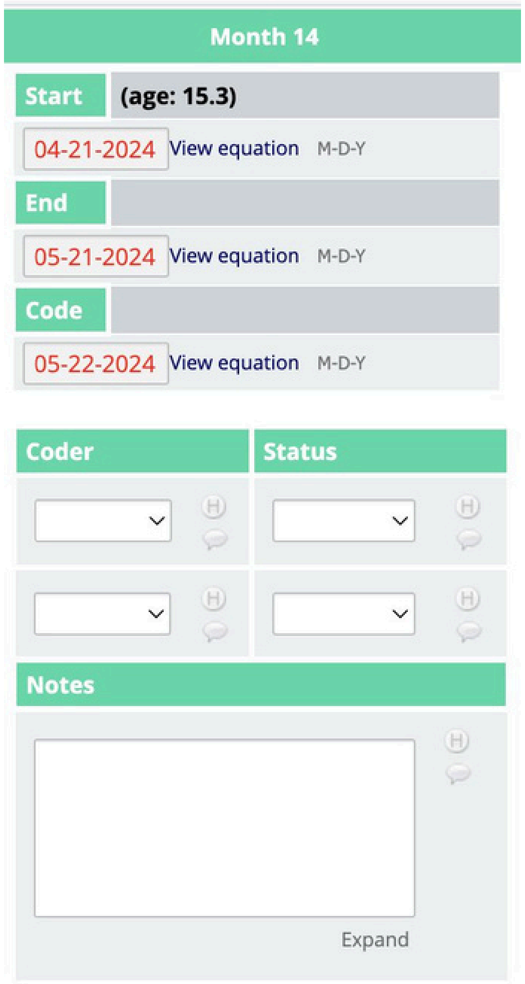

Assignment Timeline & Delayed Coding

- Coding must be completed **WITHIN A WEEK** (7 days) of the "Code" date, including the code date (see example below).
- Coding that is **NOT completed** WITHIN this time period will be marked as **"DELAY"**
- **"DELAY"** coding will:
  - Be moved to the"Delayed" coding list. Coders willmove to thislistwhen theyneed more assignments.
  - If coding isn't completed within the month, the coding will be assigned the following month when the profile reappears on the calendar (Example: Ppt 123 Month 1 coding is due to be completed on Jan. 1st. → Coding is NOT completed within seven days (by Jan. 7) → Marked "Delay" → Month 1 AND Month 2 will be coded on Feb. 1st). Therefore, coders might be assigned multiple months at once.

How do I know if coding is delayed?

Do NOT rely on the "Status" section.

Coding that is not completed on the code date may be marked as "Delayed" for tracking purposes. However, this does not necessarily mean it is actually delayed and should not automatically be coded as such.

To check if coding is delayed:

1. Checkwhetherthecodedate falls within the past week.
2. Add **six days** tothecodedate to create a one-week window (including the code date).
3. If the **current date** fallswithin this window, code the posts and profile **as normal**.
4. Ifthe **current date** is **after** this window, code the posts and profile as "**Delayed**"(see next slide).

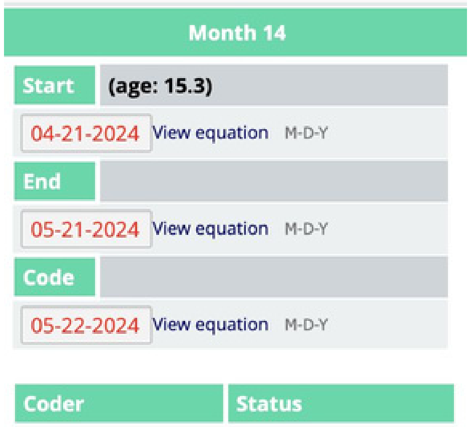

Example:
Code date is 6-22-2024
If the current date falls between 6-23-2024 and 6-28-2024 → Not delayed, code as normal
If the current date is 6-29-2024 or after → Code as Delayed

Coding Delayed Months

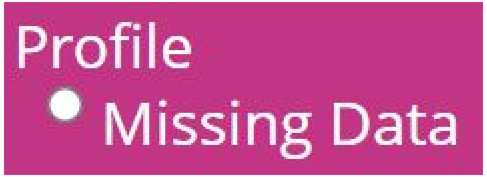

Profiles

- There is no way to know the characteristics of a profile that is being coded **greater than a week after the observation period**. Therefore, it is considered missing data.
- Open a “Profile” form and click “Missing Data”.

Posts

- Posts from past months can be identified by the date they were posted.
- Code posts as normal.

Step 2: Identify the participants' social media accounts

1. Open the REDCap project.
2. On the side menu, go to "Add/Edit records" and use the box to search the ppt's Record ID number. Click it to go the ppt's record.
3. Click the "Social Media Info" form.
4. Form will show all social media accounts shared with the research team (Instagram, Facebook, TikTok and Twitter).

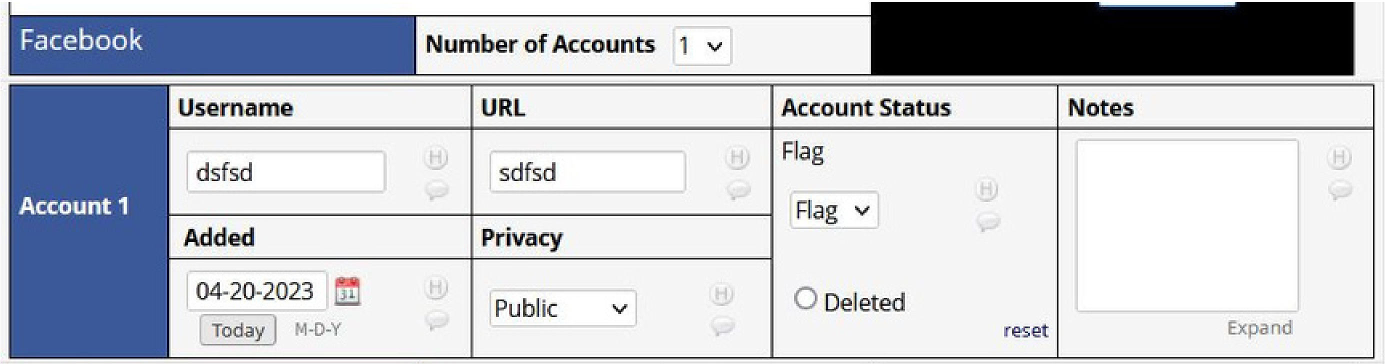

Checking Account Status

During each update call (every 6 months), participants are asked about their social media accounts to determine:

- Whether they have created any new accounts
- Whether any accounts have been deleted
- The current usage status of each account

Notes section: Used when we still follow an account, but the participant no longer uses it. This applies if the participant previously engaged with the account but has since stopped logging in, posting, or interacting, or if they deleted the app but still retain the account.

Status section: Used for accounts that are Flagged, Suspended, or Deleted—meaning the participant no longer has access to or uses the account for these specific reasons.

Follow the next slides for instructions on how to code each type of account. If both a status and notes are present, the status takes precedence over the notes.

Coding Inactive Accounts (Notes section)

Check the “Notes” section.

If an account is noted to be inactive (e.g., participant no longer uses it) but is still visible, code as usual.

This may be marked if the participant previously used this account but no longer logs in, posts, or engages with it, or if they deleted the app but still retain the account.

If both a status and notes are present, the status takes precedence over the notes. Always follow the status instructions.

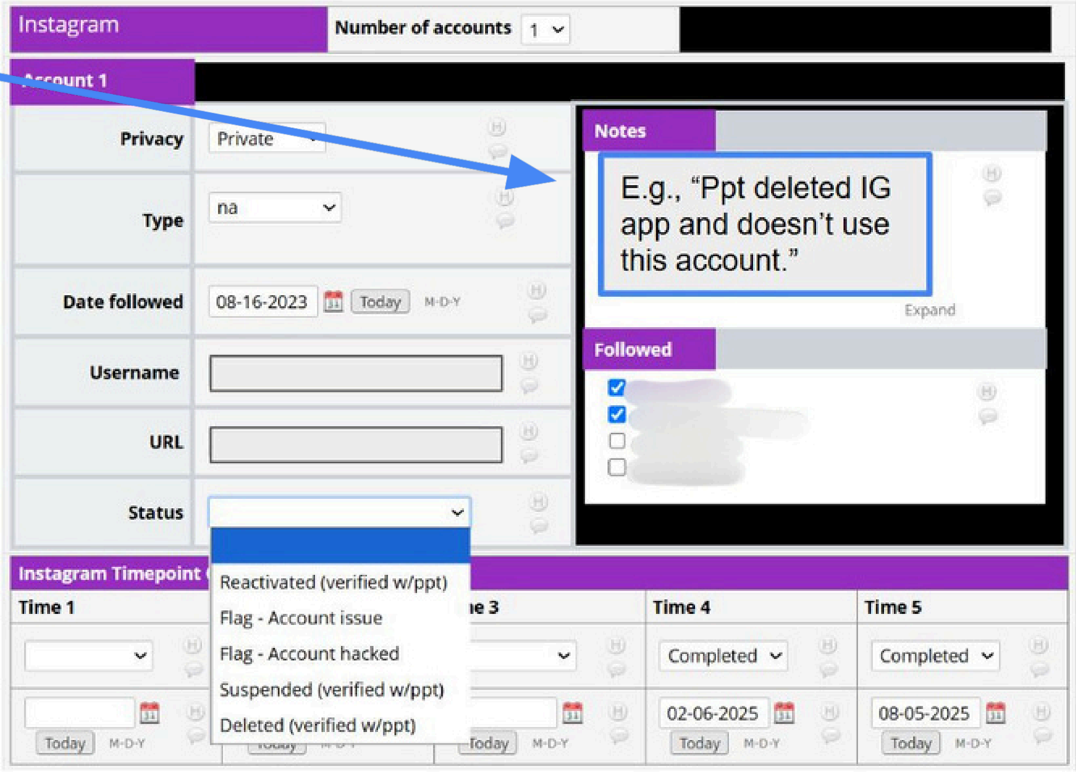

Coding Deleted/Banned/Flagged Accounts (Status section)

Under “Status,” check whether the account is marked as Flag, Suspended, or Deleted.

If so, do NOT open a profile or post form for that account. This also does NOT count as 'Missing Data' since the account is inaccessible.

If both a status and notes are present, the status takes precedence over the notes. Follow the instructions on the next slide.

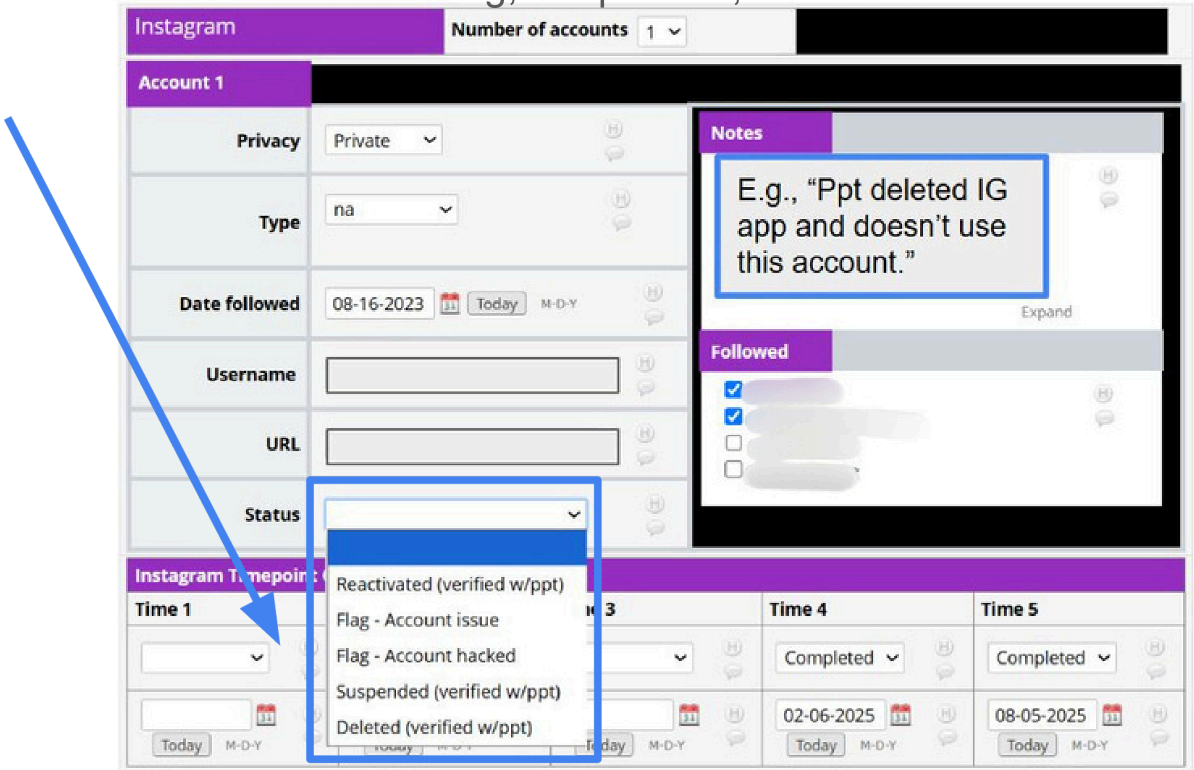

Step 3: Open the participants' social media accounts using the team profiles

- 3studyprofiles
- Our team uses [team social media account username]
- Accounts on Instagram, Facebook, TikTok, and Twitter
- Logins are in our Google Drive

**[team account 1]**	**[team account 2]**	**[backup account]**

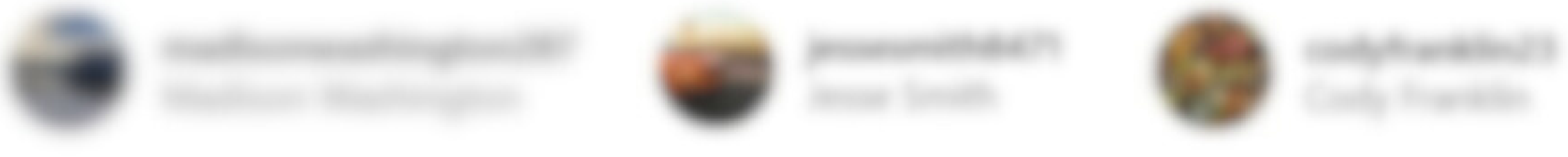

Note: Some accounts may be followed on one account and not the others.
In that case, you can use the other accounts.

Account Settings

	Privacy Settings	Friending note
**Facebook**	• Friends list hidden from public & friends.<br>• Private account (posts only visible to me)<br>• Discoverable in search (in case we need participants to find our account if we are struggling to find theirs)	Researchers send friend request and make sure they accept.
**Instagram**	• Account private<br>• Should remain at 0 followers.	Researchers follow ppt but do not allow ppt's to follow back (to keep follow list private), delete any follow back requests.
**Twitter**	• Protected account<br>• Should remain at 0 followers.	Researchers add participant to private follow list. Don't accept any follow back requests.
**Tik Tok**	• Public account<br>• Should remain at 0 followers.	Researchers follow ppt but do not allow ppt's to follow back (to keep follow list private), delete any follow back requests.

If you cannot access the participant’s profiles:

Follow the directions on the “Finding Missing Profiles” section.

If you cannot find the participant’s profile after 20 minutes of searching, ask a staff member for help. If you still cannot find the profile, leave a query for [staff] and mark the profile as “Missing Data”.

Step 4: Complete coding forms for each profile and post

1. Return to the REDCap project.
2. On the side menu, go to “Add/Edit records” and use the box to search the ppt’s Record ID number. Click it to go the ppt’s record.

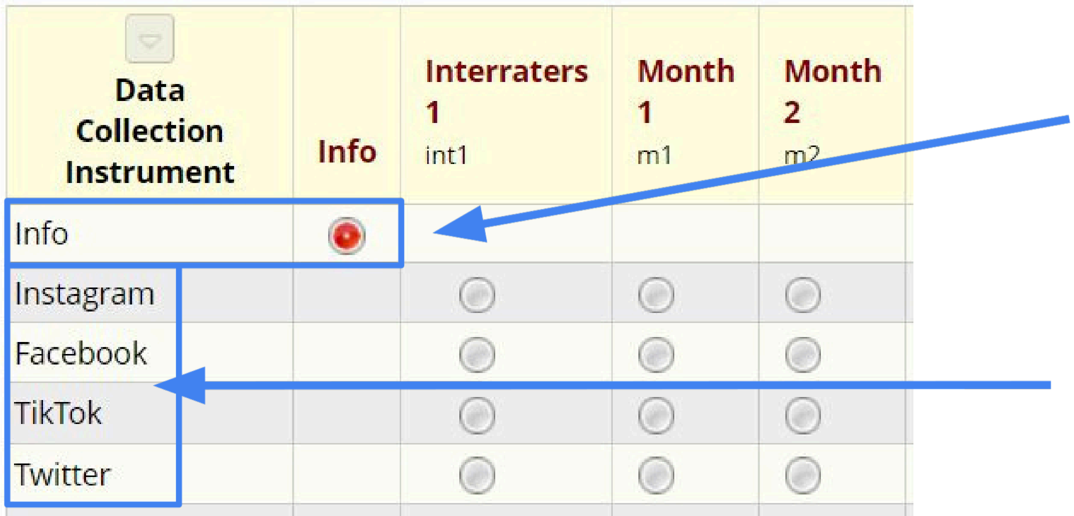

“Info” form: Basic participant information, coding schedule, and coding assignments.

Social media data collection instruments: Codingformsforeach social media platform

“Info” Page

Birthday: Check to confirm the birthday matches the profile
Observation period: Start and end date for the month-long period in which the posts are coded
Code date: Date which coding is due (day after end date)
Coder: Coder responsible for coding that month
Status: “done” “issue” or “delay”
Notes: For issues or notes for

later

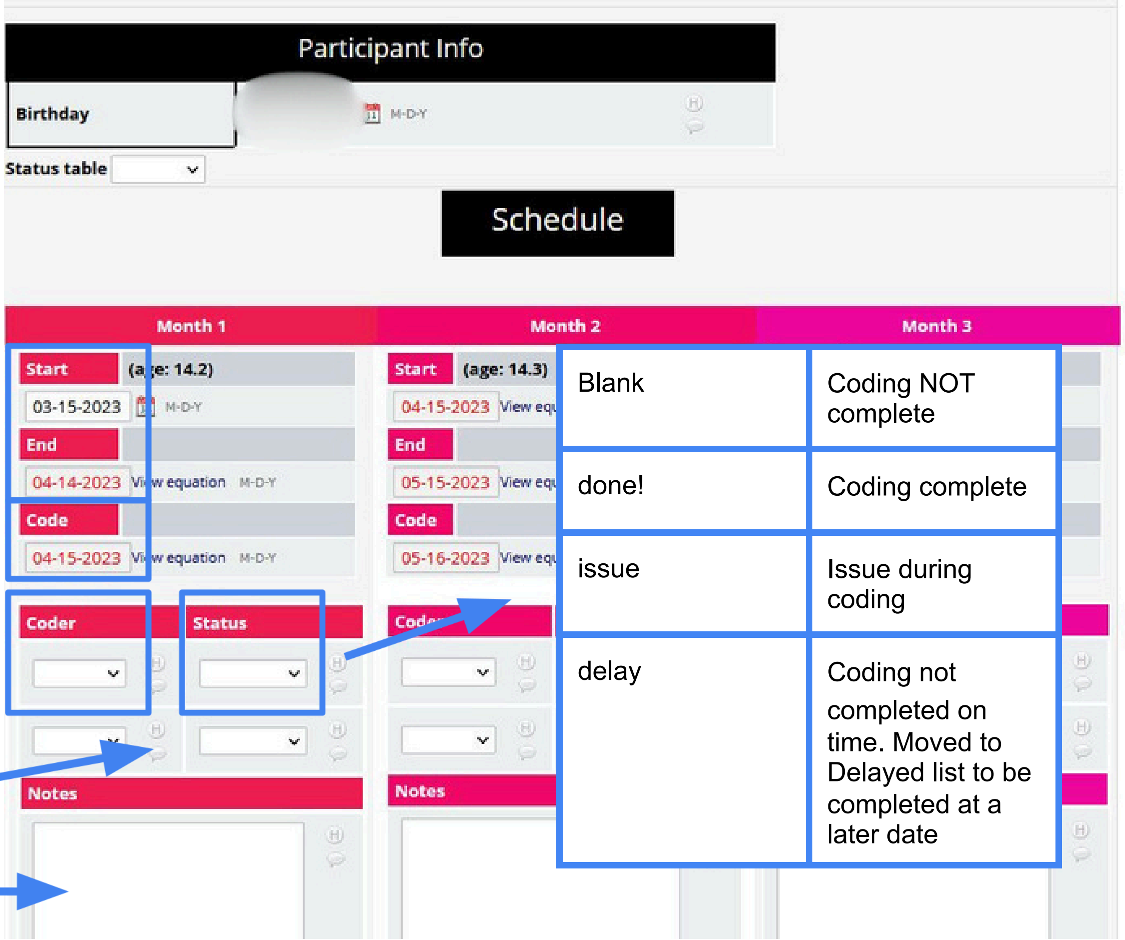

Blank	Coding NOT complete
done!	Coding complete
issue	Issue during coding
delay	Coding not completed on time. Moved to Delayed list to be completed at a later date

Opening a Form

1. In REDCap, coding forms are called “instances”
2. To open a new form (i.e., instance), click the radio button or “+” under the correct platform (row) and month (column).

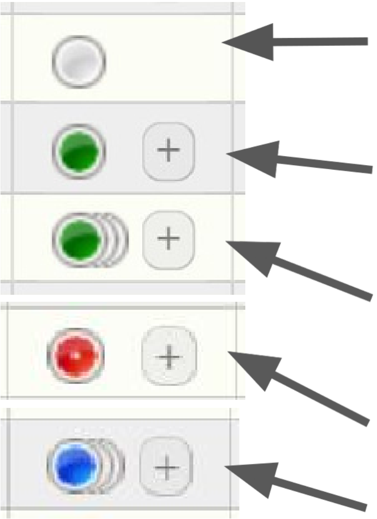

Grey: No forms complete. Click to open form.

Green: One form complete. Click “+” to open new form.

Multiple buttons: Multiple forms complete.

Red: Incomplete form.

Blue: Complete and incomplete forms.

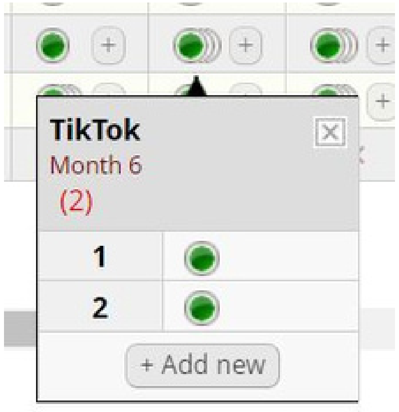

When multiple forms are complete, you can click on the overlapping buttons to see a preview of the completed forms.

Forms Types (by platform)

Instagram	Facebook	TikTok	Twitter/X
• Profile<br>• Stories (one form)<br>• Highlights (one form)<br>• Posts	• Profile<br>• Post (by ppt)	• Profile<br>• Post	• Profile<br>• Tweet/ Retweet/Quote Retweet

Each platform has unique data entry forms (i.e., instances) designed for that specific platform.

Coding Order

- Code **one account at a time**.
- Code accounts in the **order listed in the *Social Media Info* form** (Account 1, 2, 3…).
- Use **one form for the profile**.
- Use **one form per post**.
- Always start with the **profile**, then code each post in **chronological order** (oldest post to most recent post in observation period).

Coding on Instagram

Highlights and Stories are unique to Instagram.

- Stories and Highlights should be coded **before** Grid posts
- All stories from the OP are coded together on **one form**
- All highlights from the OP are coded together on **one form**

Example: A participant has 2 Instagram accounts. Account #1 has highlights and three grid posts. Account #2 has four grid posts.

#		Label	
1		Instance #1	Account #1 (Profile)
2		Instance #2	Account #1 (Highlights)
3		Instance #3	Account #1 (Post #1)
4		Instance #4	Account #1 (Post #2)
5		Instance #5	Account #1 (Post #3)
6		Instance #6	Account #2 (Profile)
8		Instance #8	Account #2 (Post #1)
9		Instance #9	Account #2 (Post #2)
10		Instance #10	Account #2 (Post #3)
11		Instance #11	Account #1 (Post #4)

After Opening a Form

Eachformwill have a section at the top:

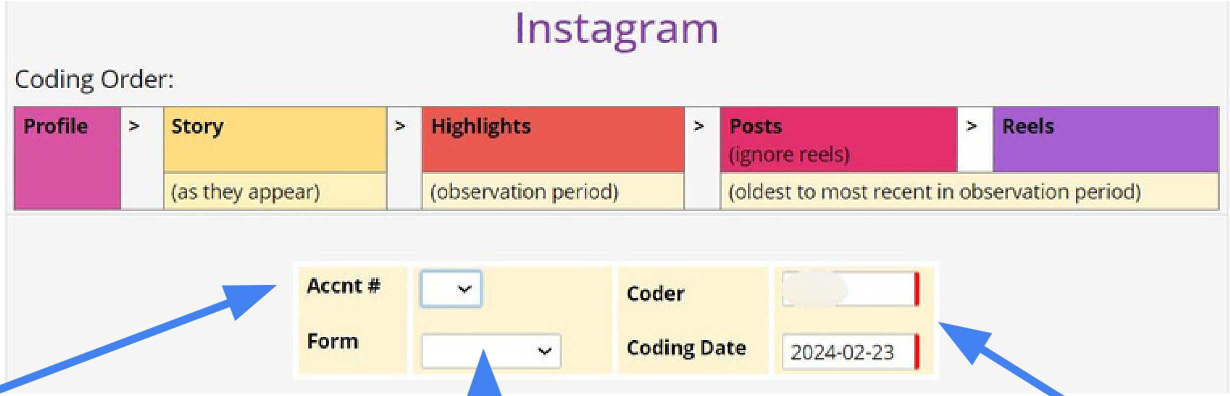

Account #: Ppt's may have multiple accounts on one platform. Indicate which account is being coded here. Account numbers go in the order of how they appear on the Social Media Info form in the REDCap project.

Form: All the possible form types you need to code (e.g., profile, post, story, etc). For each platform, this top section will indicate in which order you will fill them out.

Coder and **Coding Date**: Automatically filled with your username and the current date

Themes

- Themes are defined and described in detail in the Self-Generated Codebook For every
- theme selected, a text box will appear. Add a brief rationale (~1 sentence) for selecting the code in the textbox. Themes are the same for ALL post forms
-

****For the purposes of this training, the theme section will not be included in the coding overview since it remains consistent for each form*

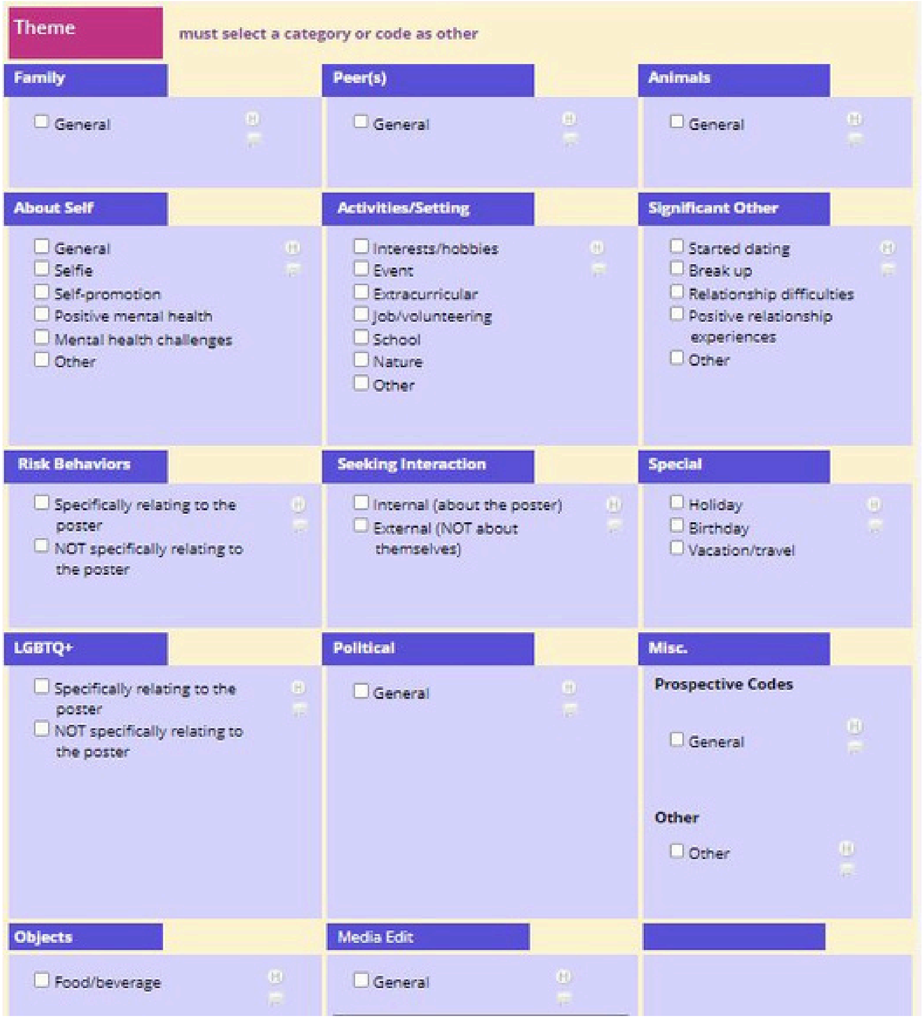

Theme — must select a category or code as other

Family
- General

Peer(s)
- General

Animals
- General

About Self
- General
- Selfie
- Self-promotion
- Positive mental health
- Mental health challenges
- Other

Activities/Setting
- Interests/hobbies
- Event
- Extracurricular
- Job/volunteering
- School
- Nature
- Other

Significant Other
- Started dating
- Break up
- Relationship difficulties
- Positive relationship experiences
- Other

Risk Behaviors
- Specifically relating to the poster
- NOT specifically relating to the poster

Seeking Interaction
- Internal (about the poster)
- External (NOT about themselves)

Special
- Holiday
- Birthday
- Vacation/travel

LGBTQ+
- Specifically relating to the poster
- NOT specifically relating to the poster

Political
- General

Misc.

Prospective Codes
- General

Other
- Other

Objects
- Food/beverage

Media Edit
- General

Step 5: Mark assignment as “Complete”

1. Mark each coding form as “Complete” as you code
2. Mark assignment as “done” in the Info form of the participant
3. Close the query to remove it from your assignment list

See the following slides for more detailed instructions.

Marking Coding Forms as “Complete”

- When the form is opened, it will automatically be marked as “Incomplete”.
- Once you have completed coding on the form, use the drop-down menu to mark as “Complete” . The form will turn green on the dashboard.
- If there are issues, keep the form marked as “Incomplete”. The form will remain red on the dashboard.

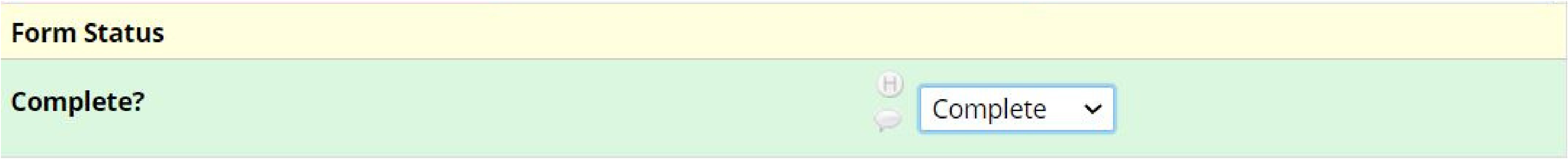

Marking Assignment as “Done” in the Info Form

1. In the “Info” form, find the month of coding you were assigned
2. Under “Coder” select your name using the drop-down menu (if is not already selected).
3. Next to your name, change the status to “done”. Coders should not choose “issue” or “delay”, only supervisors will use these statuses.
4. Click “Save & Exit Form”

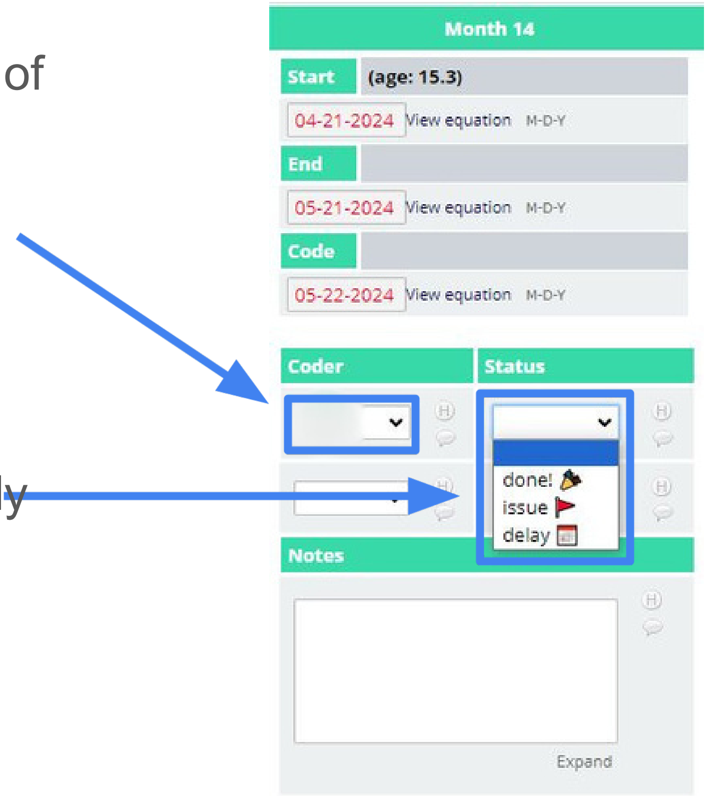

Closing Assignment Queries

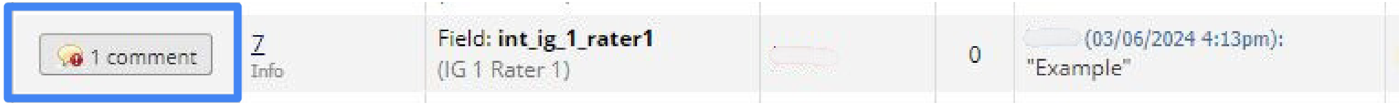

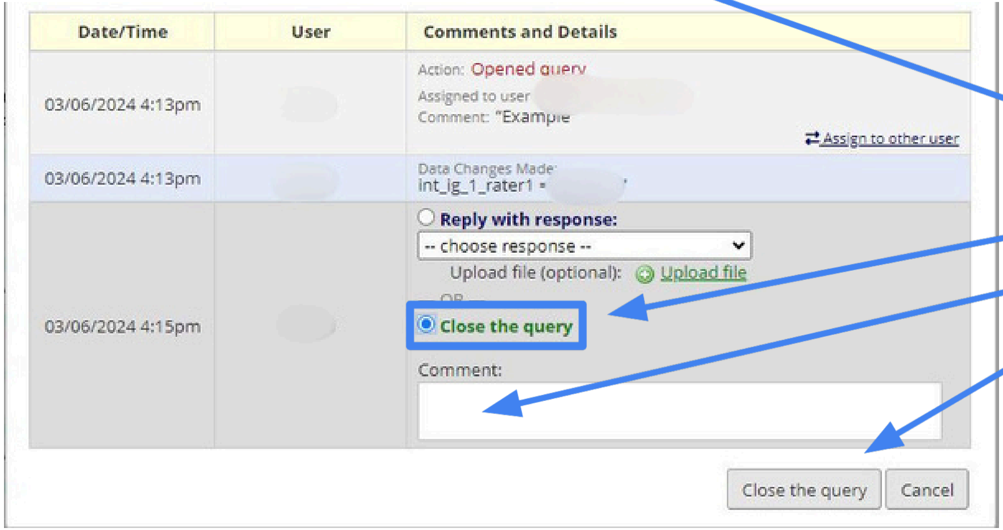

1. Click "Comment" button next to
2. Record ID
3. Select "Close the query"
4. In comments, write "Done"
5. Click "Close the query"

Assignment will disappear from "[Your Name]'s Assigned" list

NOTE:

All profile and post forms use most of the same fields, with the exception of platform-specific fields.

Fields will be explained the first time they appear, primarily in the Instagram section. For the remaining platforms, the only fields covered will be those that are appear for the first time and are specific to that particular platform.

See Instagram section for detailed explanation of all other fields.

Instagram Coding

Coding Order

Instagram

Coding Order:

Profile	>	Story	>	Highlights	>	Posts (ignore reels)	>	Reels
		(as they appear)		(observation period)		(oldest to most recent in observation period)		

****Reels sometimes appear on the ppt's grid. When coding posts, skip the Reels and return to code them after coding the posts.*

Instagram - Profile

Profile

Describe profile photo. Click "same as last month" if the profile photo is the same from the previous observation period. ALWAYS include a description in the text box (even if you copy and paste it from the month before).

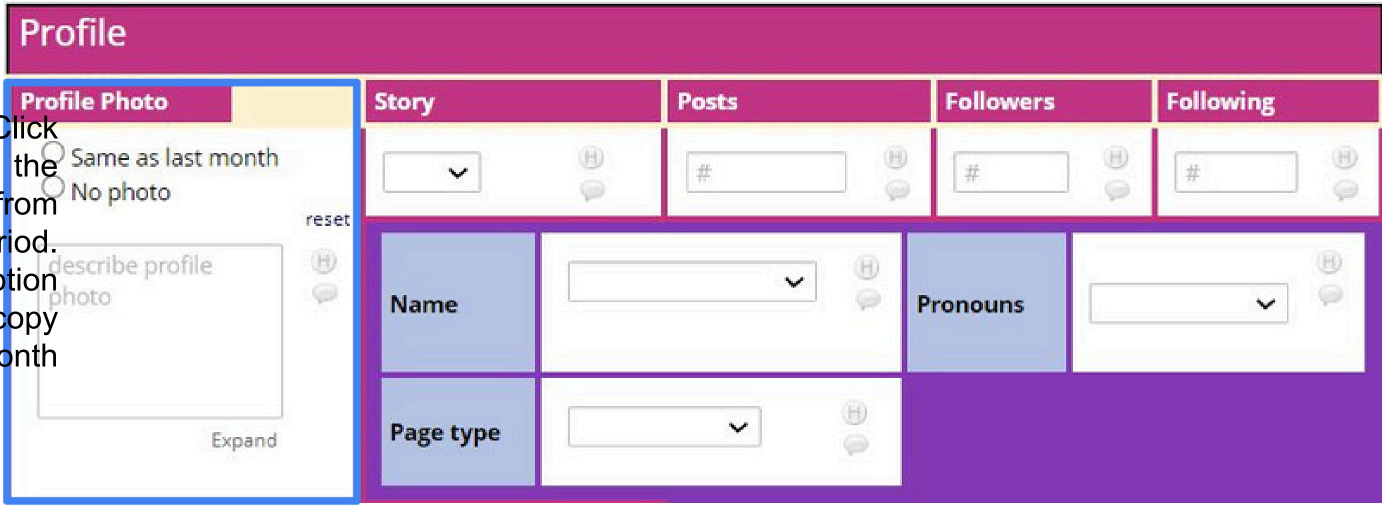

If there is no profile photo or cover photo, click "No photo" and do NOT type anything in the text box.

Example:

"Ppt smiling posing in front of a white brick wall."

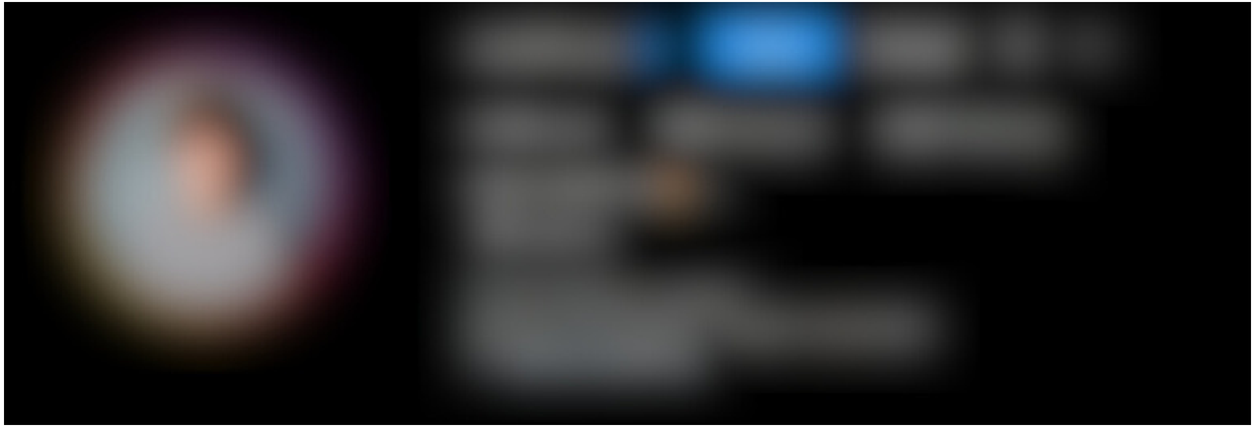

Profile

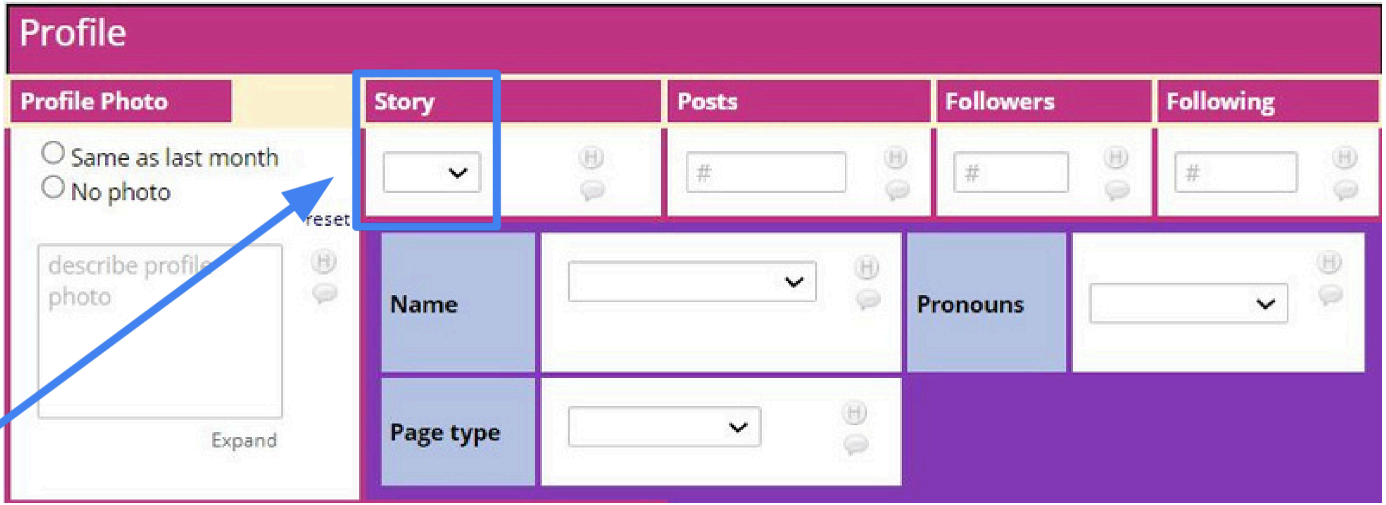

Story: yes/no
Will have a multi-colored circle around the profile picture

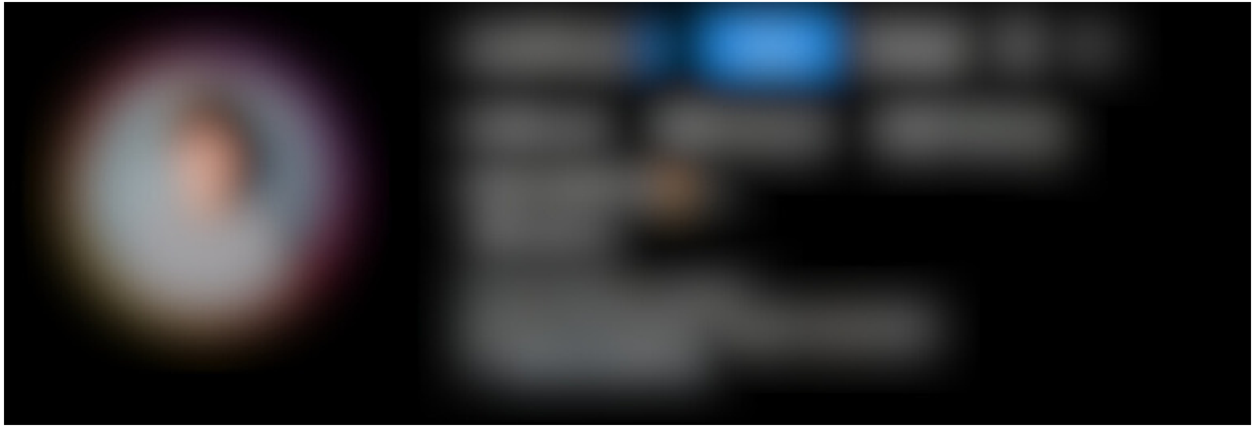

Profile

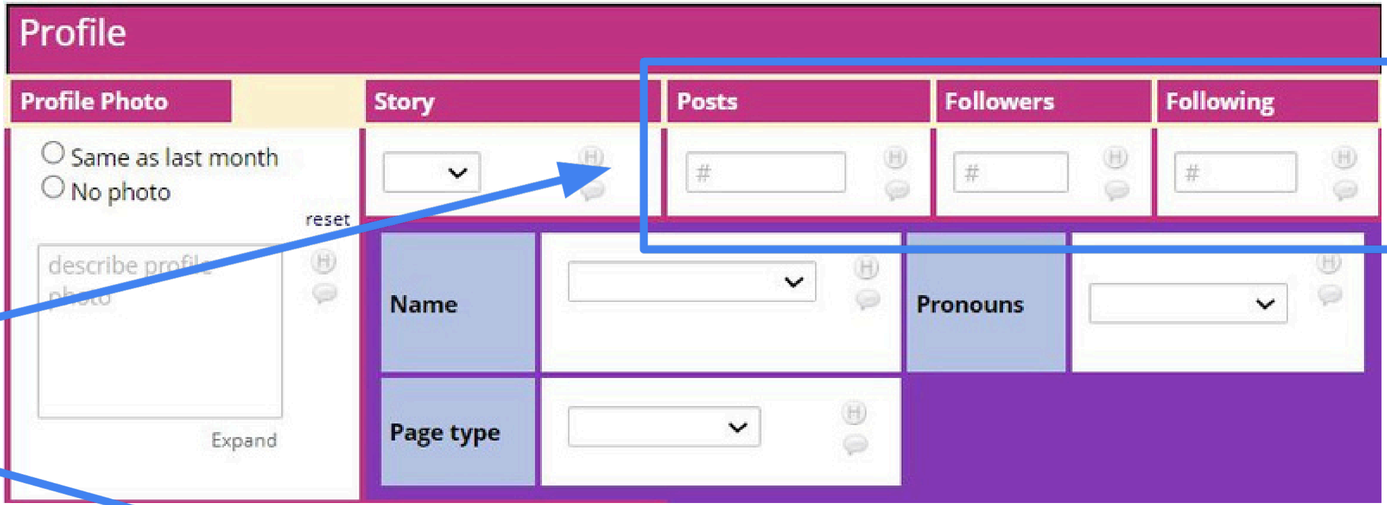

Posts, Followers, Following
Write full numbers without abbreviations or commas
5,329 → 5329
365k → 365000

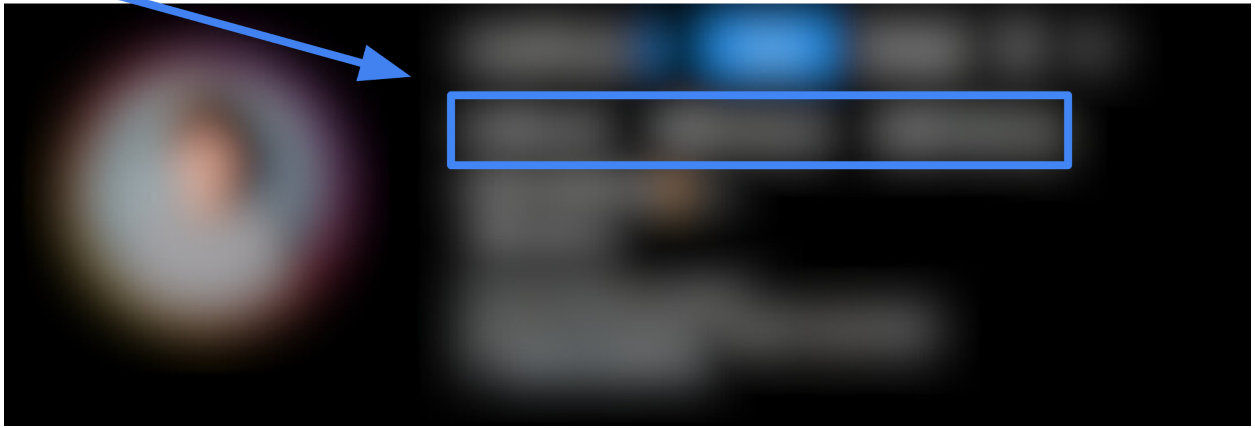

Profile

Name: Everything written in the “Name” field (NOT the username)
Located at start of bio.
Disregard numbers, emojis, etc. Names can be nicknames (e.g., Steve instead of Stephen)

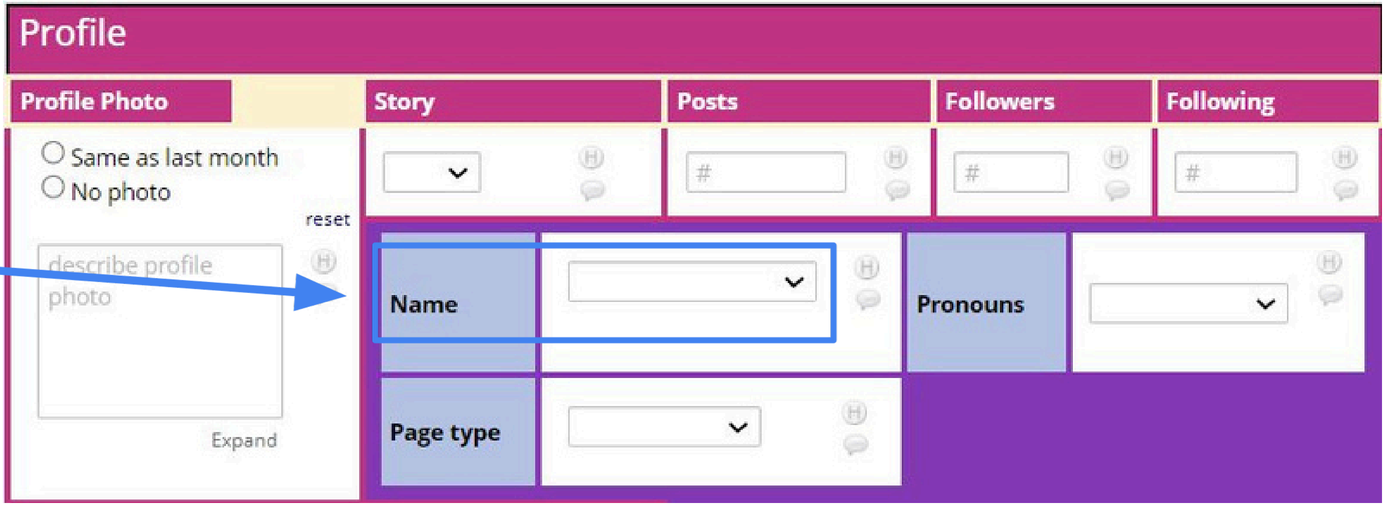

- **Full name** *(first and last name in REDCap)*
- **First name** *(includes nicknames derived from first or last name in REDCap)*
- **Name related** *(includes some or all of name in a phrase, e.g., “Stevieboy”, other names not in REDCap)*
- **Not name related** *(e.g., celebrity/character name, object)*

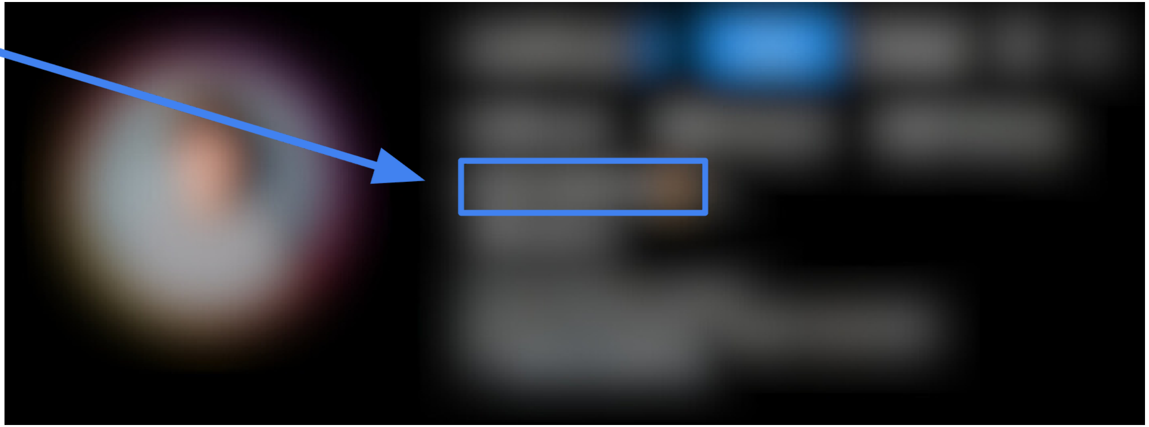

Profile

Pronouns: If listed. If not, leave blank.
Page type: If there is a page type listed, choose “Professional” and enter the title used. If not listed, select “Personal” e.g., “Musician/band”

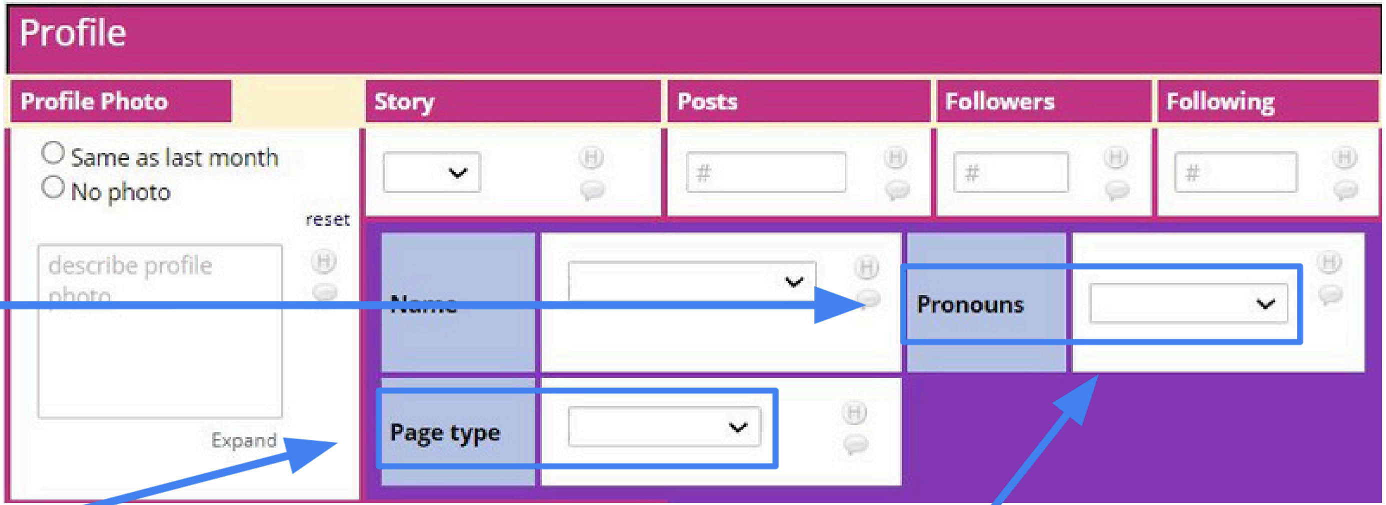

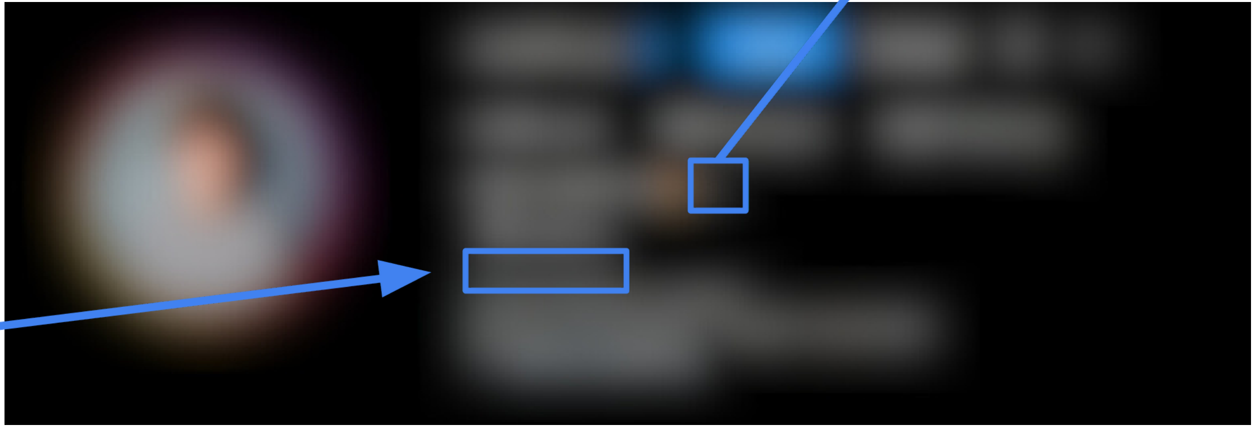

Profile

Bio:

- Copy & paste the bio
- Remove identifiers (e.g., highschool, where they live, handles, etc.)
- Click “same as last month” if the profile photo is the same from the previous observation period.
- No bio/bio is blank: Click “No bio” and do NOT write anything in text box.

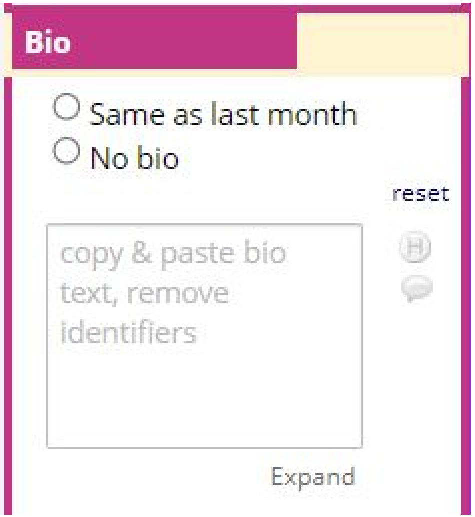

Example:
“ontour near you in october !
Grew up on the internet - wouldn’t recommend
[link tree].”

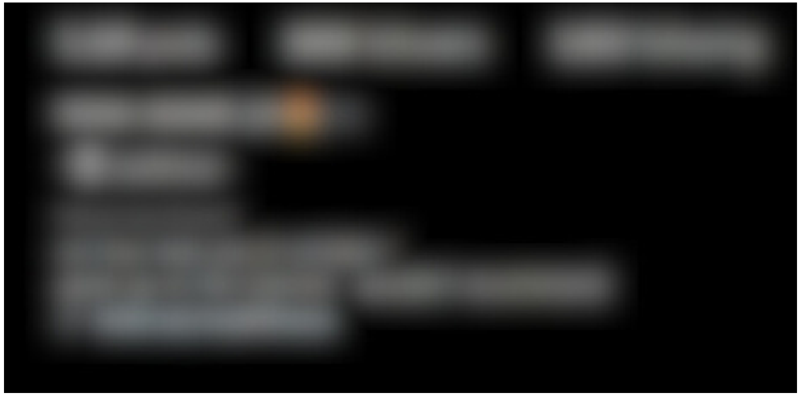

Bio Codes

- **Birthday/age:** Select “Actual” or “Not actual”. Check the top of the “Info” form to see if it birthdate is accurate or not. Leave blank if not listed.
- **Relationship:** Select “Single” or “Taken”. Leave blank if not listed.
  - **Link/partner handle:** Partner’s handle or “@”
  - **Anniversary date**
  - **Other**
- **School**
  - **School name:** Full name or acronym
  - **Graduation date:** e.g., ‘26

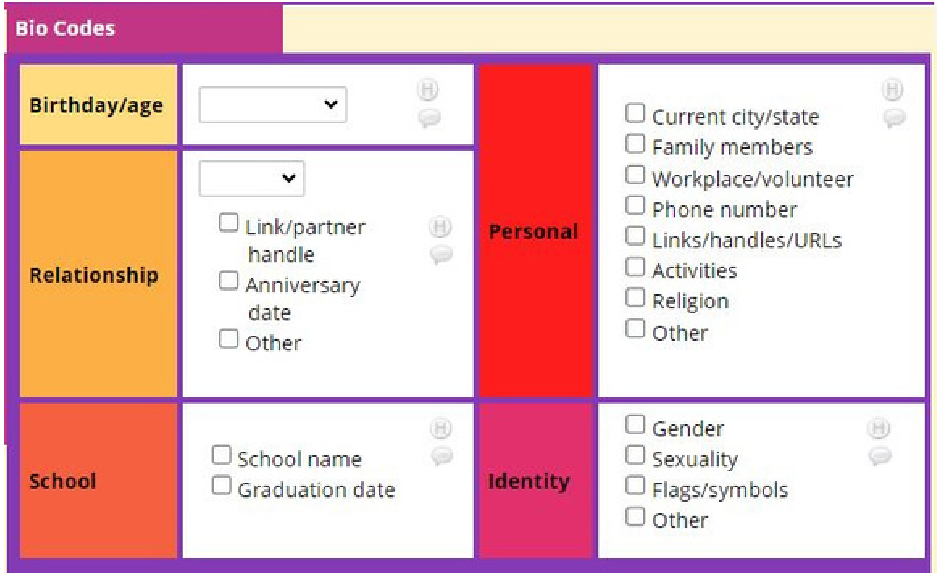

Bio Codes

- **Personal:** personal info about the ppt in their bio.
  - **Current city** (e.g., "📍608")
  - **Family members**
  - **Workplace/volunteer**
  - **Phone number**
  - **Links/handles/URLs** (e.g., LinkTree, YouTube, Snapchat, etc.)
  - **Activities** (e.g., extracurriculars, hobbies, interests, etc.)
  - **Religion** (e.g., Bible verses, religious emojis, etc.)
  - **Other**
- **Identity:**
  - **Gender** (NOT pronouns)
  - **Sexuality**
  - **Flags/symbols**
  - **Other**

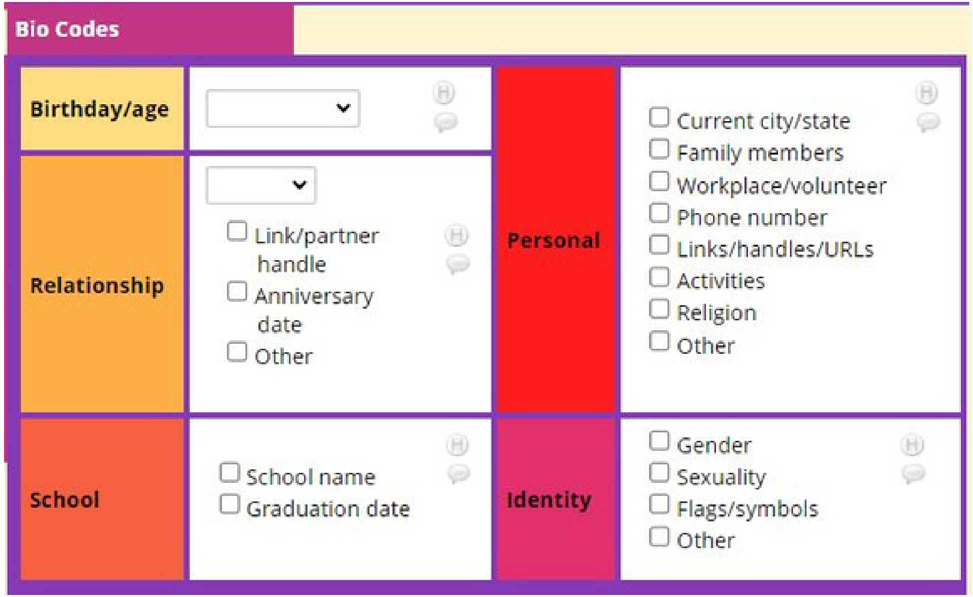

Always DE-IDENTIFY information on the form by replacing it with a description of the information. (E.g., WI → "Ppt's state" or @mysnapchat → [ppt's Snapchat])

Tagged tab: total # of posts in tagged tab

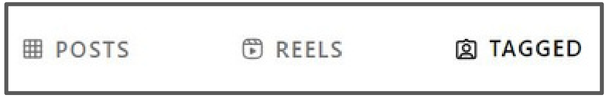

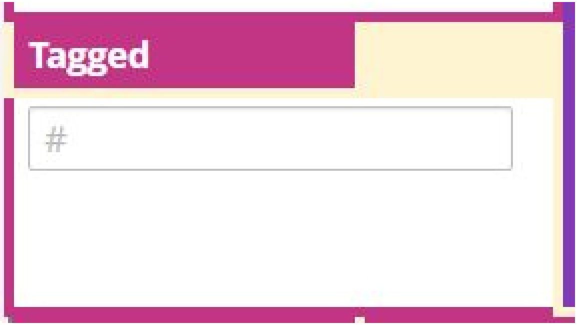

Profile Notes: Briefly summarize your overall impression of the account and any key observations and interpretations. Consider noting things like:

- **Topics they post about**
- **Types of posts**
- **Posting frequency**
- **Overall vibe or aesthetic**
- **How they use the account**

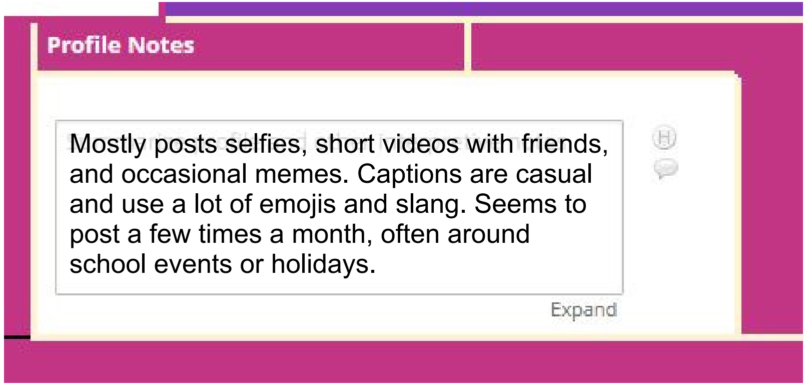

Instagram Reposts *(starting 10/24)*

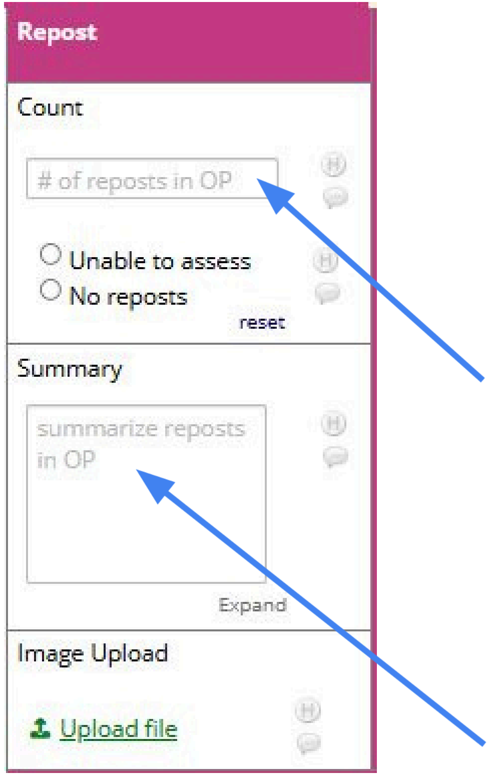

First Month of Coding

Leave "Count" and "Summary" blank. Only complete the "Image upload". Instagram reposts do NOT show the date they are reposted. Therefore, we need to use a reference post to determine the first (and last) repost made in the OP. Without a reference post, we cannot get an accurate count or summary of Instagram reposts. However, coders DO need to complete the "Image upload" to make this possible in the following months.

All Following Months

Count: Open the profile coding from the previous month. Open the file uploaded in the "Image upload" field. Locate the most recent repost from the screenshot on the ppt's current repost page and use that as the marker for the BEGINNING of the OP. Every post that appears AFTER that reference post is considered to be made in the OP. Count and enter the number of reposts in the OP.

- **Unable to assess:** Select if you cannot locate a post from the past OP in the screenshot to use as a marker, and therefore cannot count the number of reposts during this OP. This May occur if posts were deleted, un-reposted, hidden, or if the ppt is a frequent reposter.
- **No reposts:** Select if the Reposts tab is not visible/there are no reposts on the account.

Summary: Describe the common themes in the reposted made during the OP

How to Upload a Screenshot

1. Take ascreenshot oftheppt'sInstagramrepostpage (see next slide for example)
   a. Use the computer searchto openthe "Snippingtool"
   b. Click "New"
   c. Drag the window to capture as many of the recent Instagram reposts as possible
   d. Do NOT include ppt's profile information (i.e., the header with the username, profile picture, and bio) in the screenshot
2. Save the screenshot
   a. Savethe screenshot to the desktop
   b. Rename the screenshot "rec ID_month number_igreposts" (e.g., 123_m3_igreposts)
3. Upload the screenshot to REDCap
   a. Inthe "Image upload" field, click "Upload file"
   b. Click "Choose file"
   c. Navigate to the "Desktop" folder and select the screenshot
   d. Click "Upload file"
4. DELETE the screenshot from the computer desktop IMMEDIATELY after uploading to REDCap

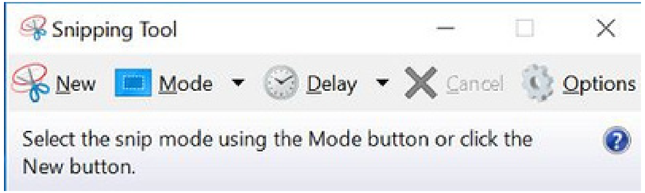

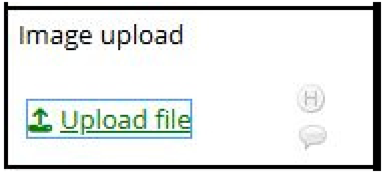

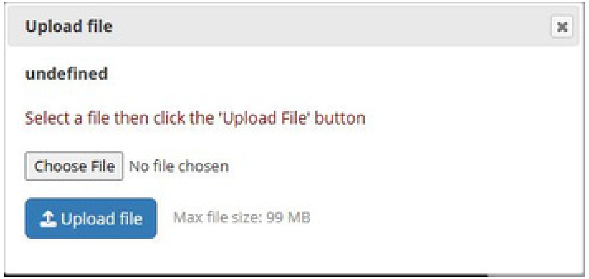

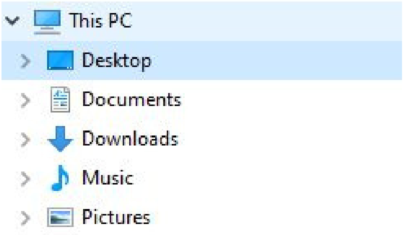

Do NOT include ppt's profile information (i.e., the header with the username, profile picture, and bio) in the screenshot

DO include as many reposts as possible. To maximize the number of posts shown on the screen, press the three dots on the top right of the Google Chrome browser at zoom out to 80%.

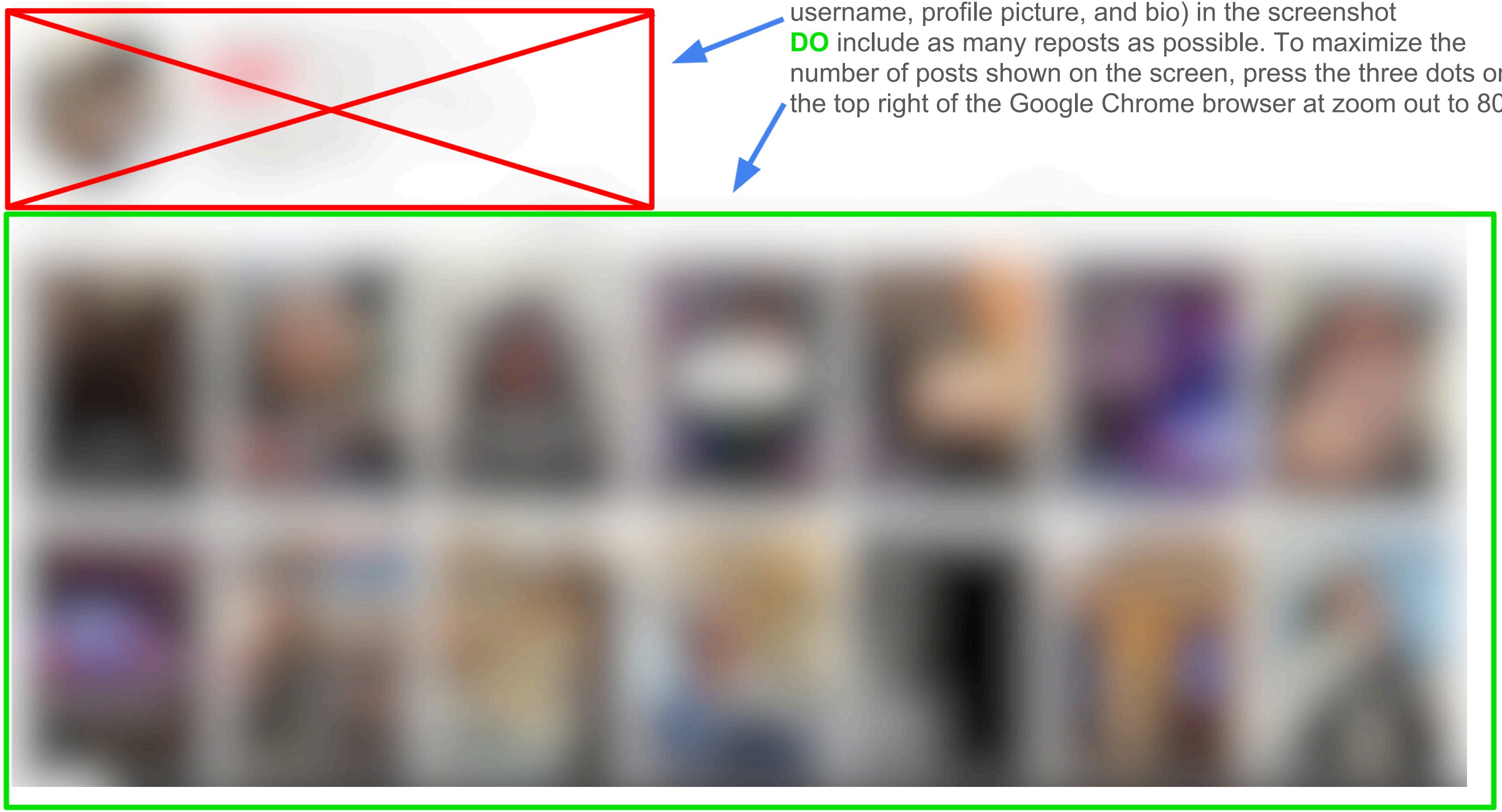

How to View a Screenshot

To view a screenshot of reposts, click the file uploaded. If you get this error, do the following:

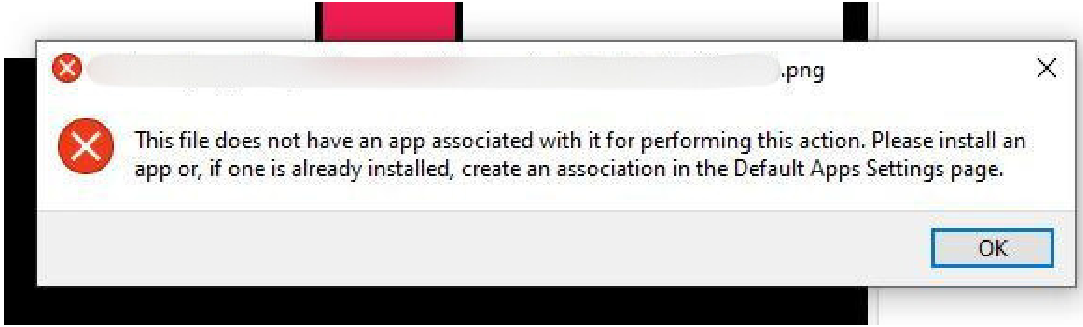

Open File Explorer: Apps > Default Apps > Photos (mountain and moon icon) > Scroll down to .png > Select Photos as the default app

Following List

Participants' following lists are recorded every 6 months. If you are assigned coding for *Month 1, 6, 12, 18, or 24*, you must record the following list. For all other months, the following list will not be recorded.

To record the following list: Open the following list. Copy and paste as many usernames as possible into the appropriate box. On most platforms, you can highlight the first username and drag downwards to select the rest of the usernames. This can take some time—at most, spend 10 minutes on this task. For participants that follow many accounts, every account followed may not be captured. On Facebook, use the "Following" list WITHIN the Friends list.

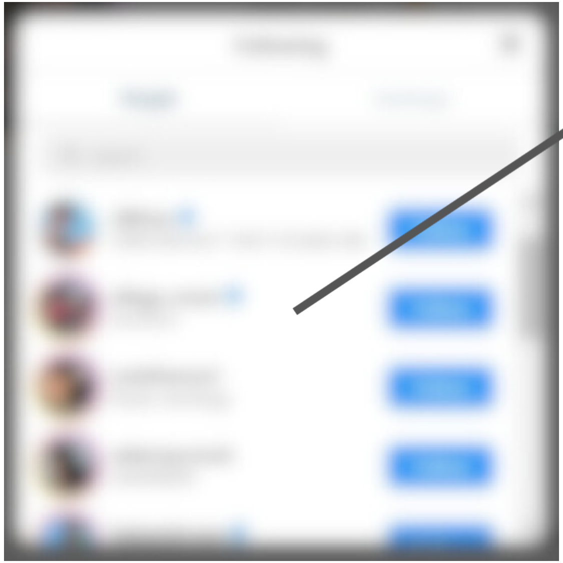

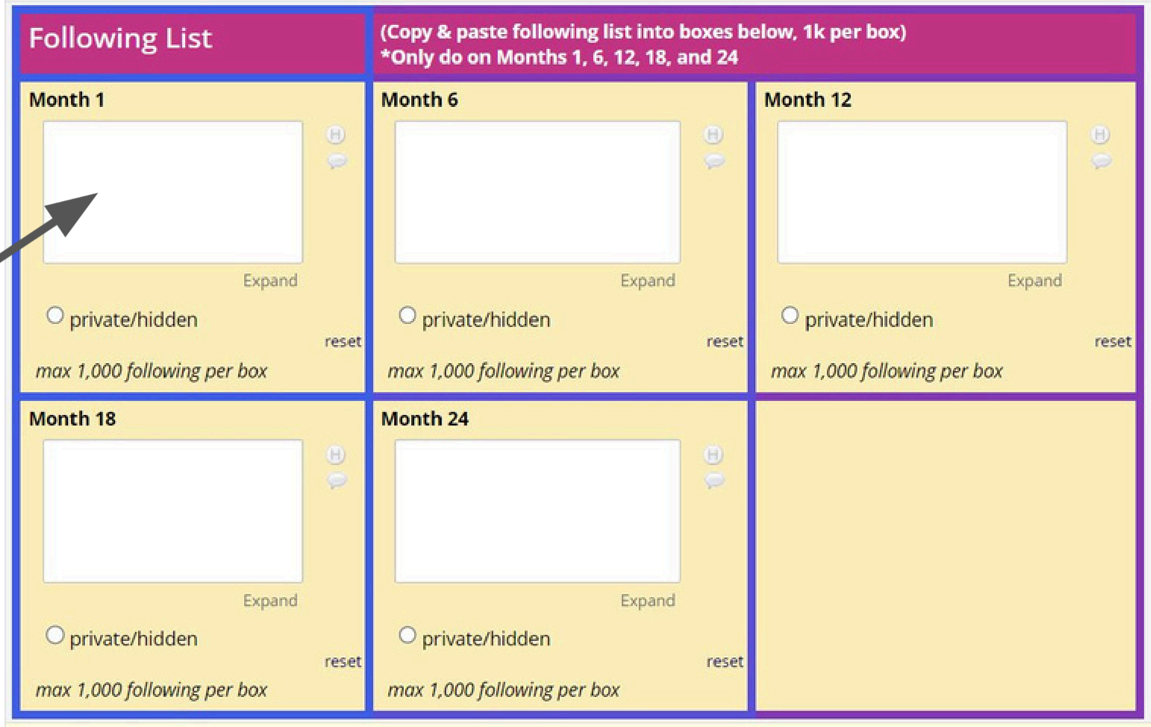

Instagram - Stories

Stories

1. Complete a Story form only if the participant has an active story at the time of coding.
2. Do not code stories for delayed months (i.e., months coded more than one week after the observation period), even if the story is still visible on the profile.
3. Code all posts within a participant's story together as a single post.

# of Stories: Number of posts on ppt's story

Description: Summary of the stories e.g., Posted a link to an article on black lives matter, 2 videos of ppt and peers/siblings walking a dog

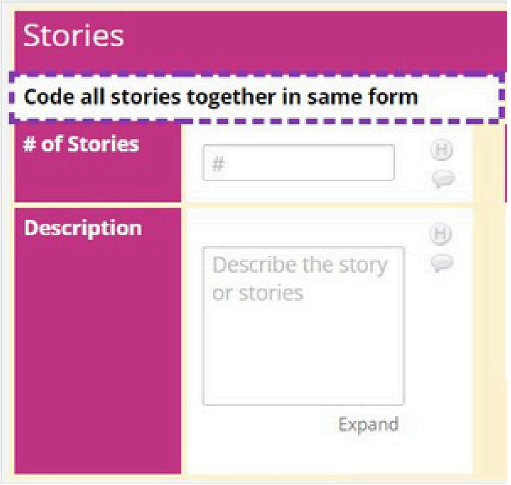

Story indicated by multi-colored ring around profile picture

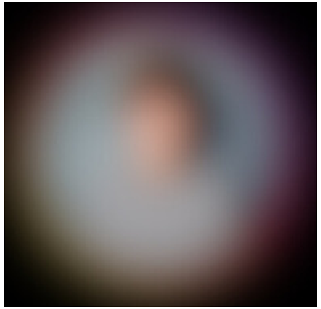

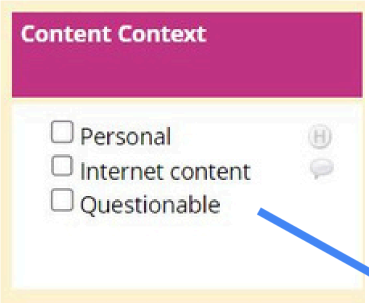

**Personal**	Directly relates to participant	Example:<br>• Selfie<br>• Family photos<br>• Text/video/images from the poster
**Internet content**	Related to the Internet	Example:<br>• Reposted memes<br>• TikToks from other pages<br>• Links to YouTube videos not made by poster
**Questionable**	Contextneitherpersonal or internet	
***Both (can select multiple)**		Example: Meme posted from another page, participant tagged sibling saying “This is us”

Format if applies

☐ Image ☐ Video ☐ Album/slides ☐ Screenshot ☐ Filter/effect ☐ Collaborative
☐ AI ☐ Other platform

**Image**	Still image, photo, graphic, etc.	
**Video**	Video, GIF, etc.	
**Album/slides**	Sharing multiple photos/videos that can be clicked through in one single post (albums, slides, carousels, etc.)	
**Screenshot**	Screenshot showing contents on phone display	Examples:<br>• Screenshot of a post on another platform<br>• Screenshot of DMs
**Filter/effect**	Discernable filter or effect added to the image	Example:<br>• Comedic filters<br>• Beauty filters<br>• 3D effects
**AI**	Suspected AI-generated image or video.	*Note*: AI-generated content should be treated as artistic representation when coding themes.
**Other platform**	Content shared from another platform (TikTok, Twitter, Facebook, Snapchat, Other)	Example:<br>• Reposted TikTok<br>• Screenshot of Tweet
**Collaborative**	Posts co-authored with other accounts using the Collab feature.	Note: See Coding Guide for further instructions and guidance.

Collaborative Posts

- A collaborative post on Instagram is a shared post created by two or more accounts.
- One account creates the post and invites another account to collaborate. The post appears on both profiles and is shared with both sets of followers.
- All collaborators can see the same likes, comments, and engagement.

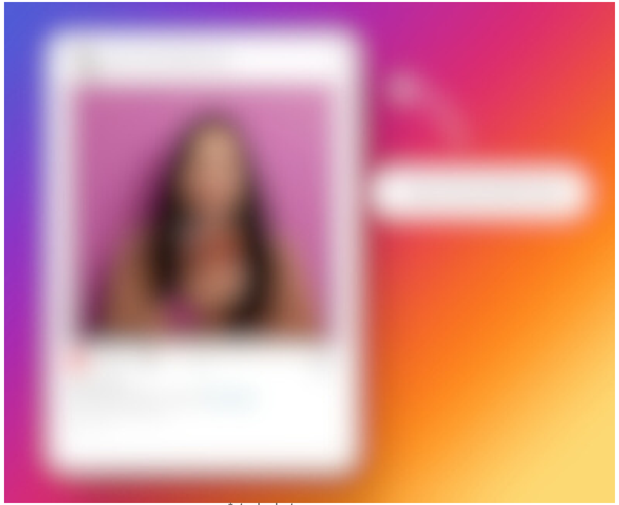

*stock photo

How to code:

- Under “Format” → Select “Collaborative”
- Collaborative posts sometimes focus on the collaborator and not the participant. Make sure to code posts as they are related to the participant.

Additions if applies

☐ Sound ☐ Location

**Note: Additions must be added using an embedded, in-app feature, and must be integrated into the platform being coded.*

**Sound**	Ppt	Organic audio originating from the participant (e.g., ppt talking)
	Music	Songs and instrumentals
	Other media	Audio from other sources in media and the internet (e.g., viral TikTok audios, TV show dialogue, Kardashian quotes, etc.)
	Sound not available	Title of the sound is not discernable (“Sound isn’t available” on TikTok)
**Location**	Location added using a geotag	Example: Lake Tomahawk, Wisconsin<br>*No text box due to the risk of potentially identifying information*

Instagram - Highlights

Highlights

1. Complete a Highlight form if the participant has highlight sets visible on their profile at the time of coding, even if none were posted during the observation period.
2. Thematically code only the content of highlights that were posted during the observation period.
3. Code all posts within a highlight set together as a single post.

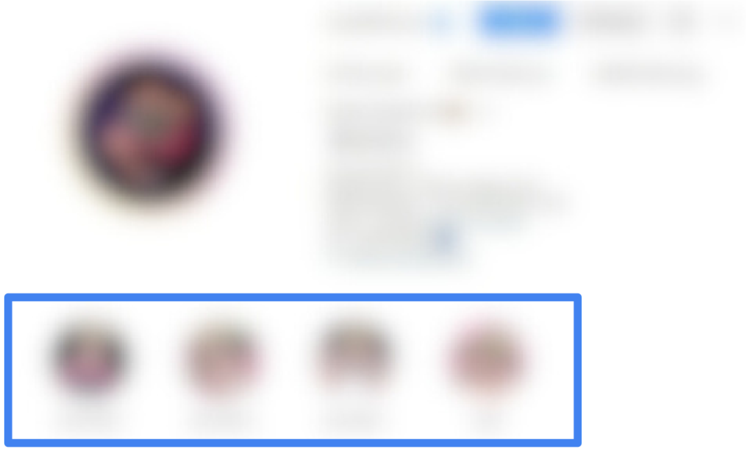

To view the specific date a post was added to a highlight, hover the cursor over the amount of time listed next to the highlight. A white box will appear with the date.

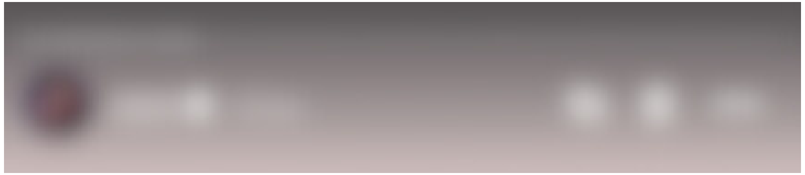

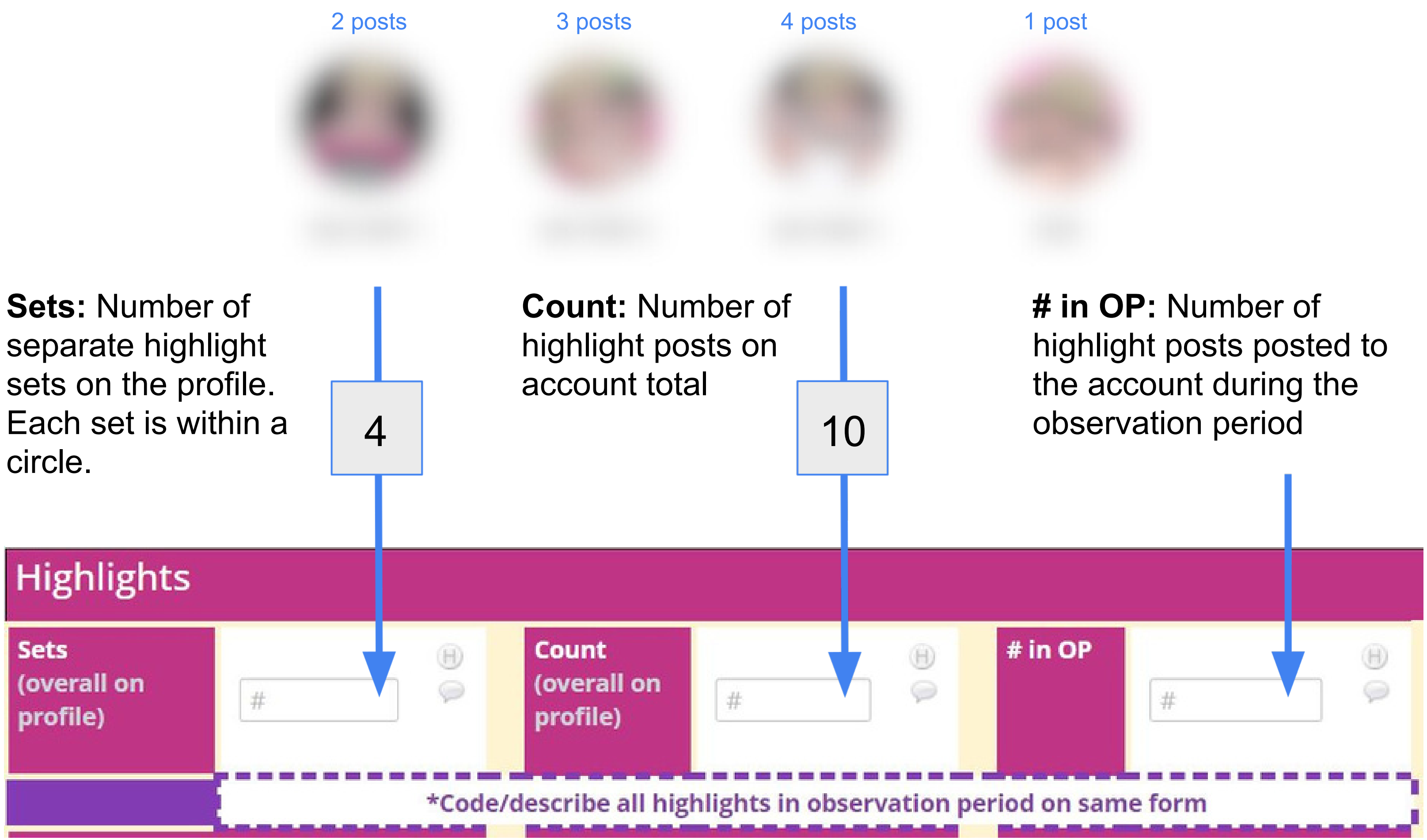
2 posts
3 posts
4 posts
1 post
Sets: Number of separate highlight sets on the profile. Each set is within a circle.
4
Count: Number of highlight posts on account total
10
in OP: Number of highlight posts posted to the account during the observation period
Highlights
Sets (overall on profile)
#
Count (overall on profile)
#
in OP
#
*Code/describe all highlights in observation period on same form

Instagram - Posts

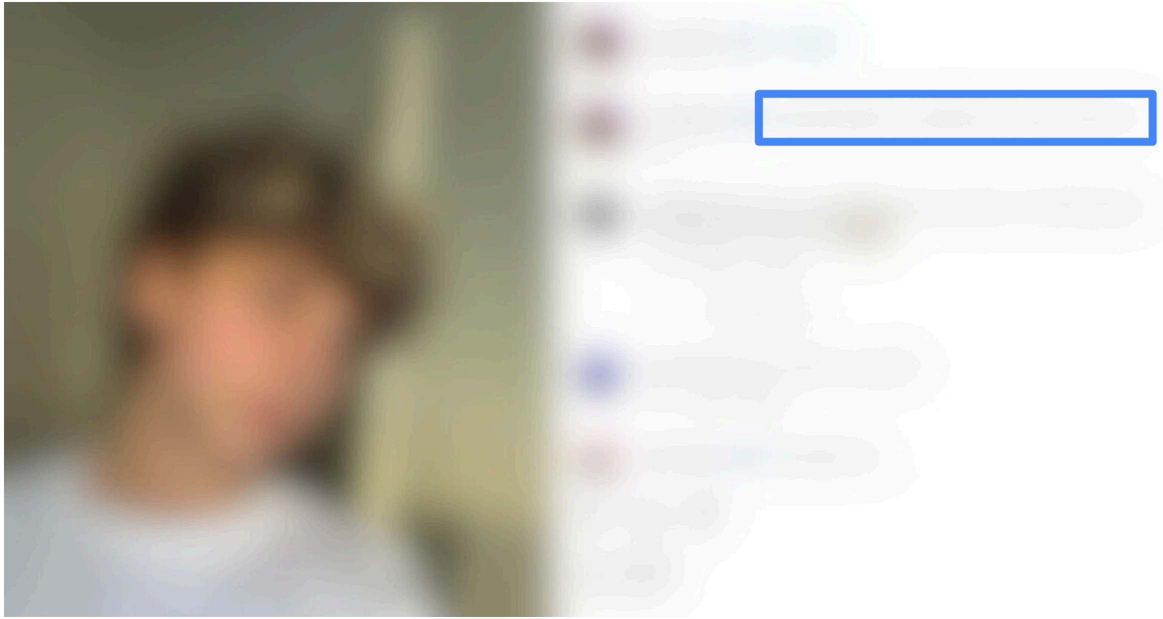

Caption

copy & paste caption text, remove identifiers

E.g., “I GAVE MYSELF A HAIRCUT DO YOU LIKE IT?!”

Expand

Description

describe post media

E.g., “Selfie of ppt looking off to the side, not smiling. Taken in a bathroom.”

Expand

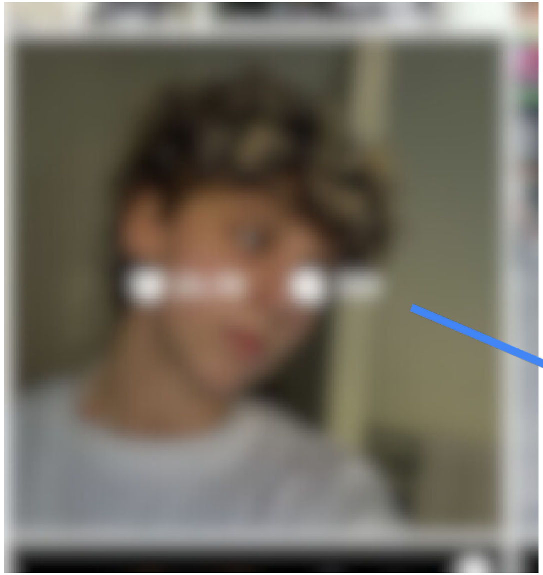

# tags: Number of accounts tagged *directly on the post* using an embedded feature (including chips and the Collab posting feature)
Views: Sometimes shown instead of likes (not common)
Likes and **comments:** Can be viewed by hovering over the post in the gridview OR click the three dots in the right corner of a post and click “Go to post”. Comments are sometimes missing from the preview.

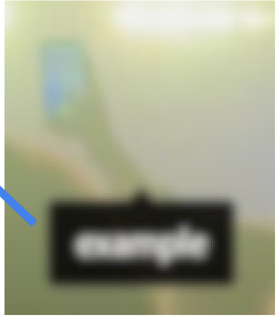

*stock photo

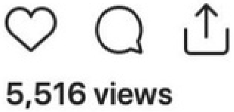

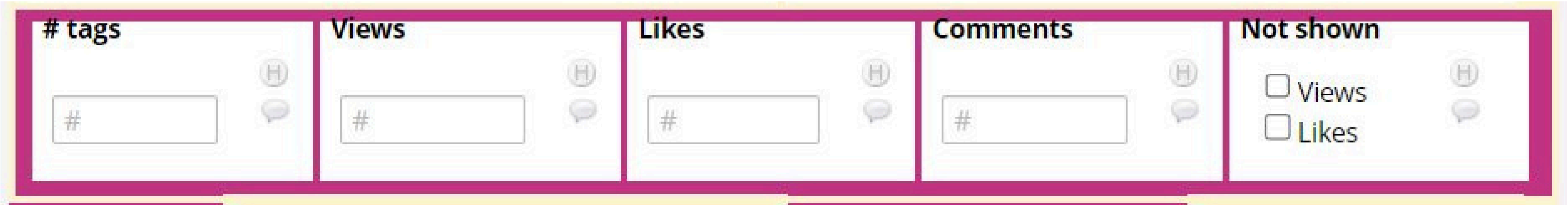

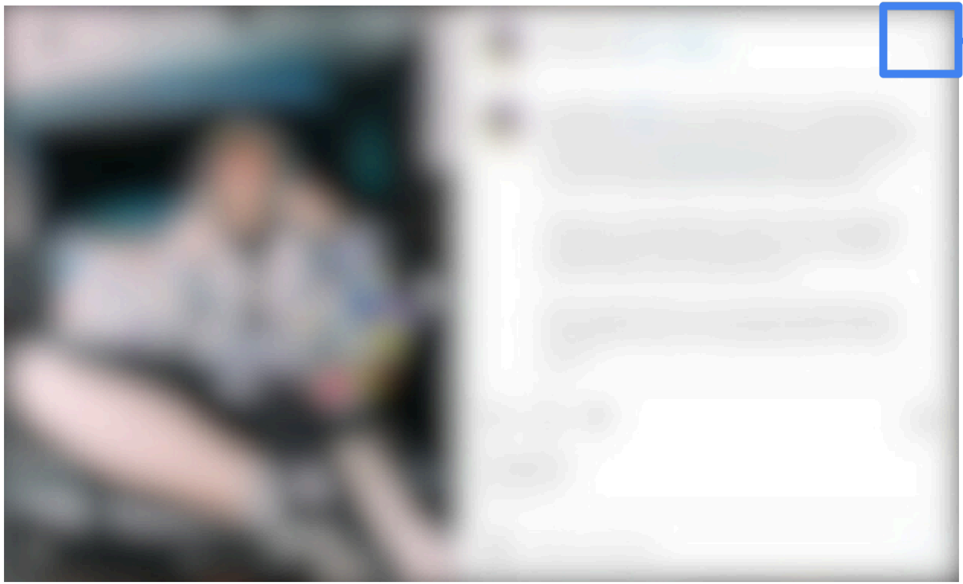

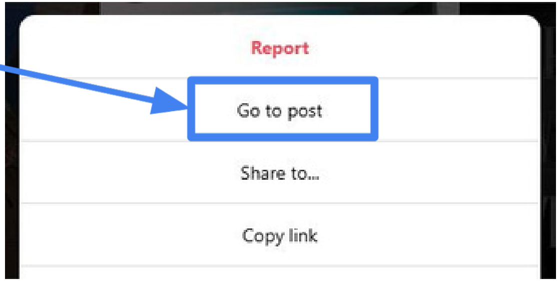

When you click on a post from a participant’s profile, you'll see a preview of the post. However, this preview may exclude comments and other details. To ensure accurate coding, **clickthethreedotsin the upperright corner and select “Go to post.”**

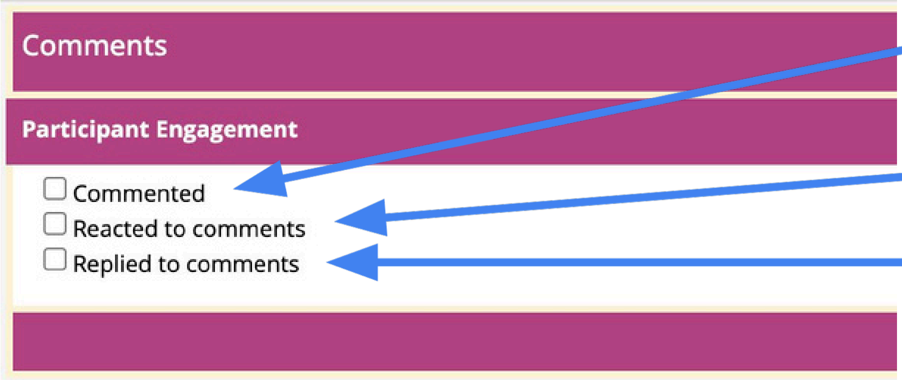

Commented: Participant comments on their own post (NOT as a reply an existing comment)
Reacted to comments: Ppt likes a comment
Replied to comments: Ppt replies in the comments. Replies are preceded by the @handle of the person being replied to

Facebook Coding

Facebook - Profile

Coding Order

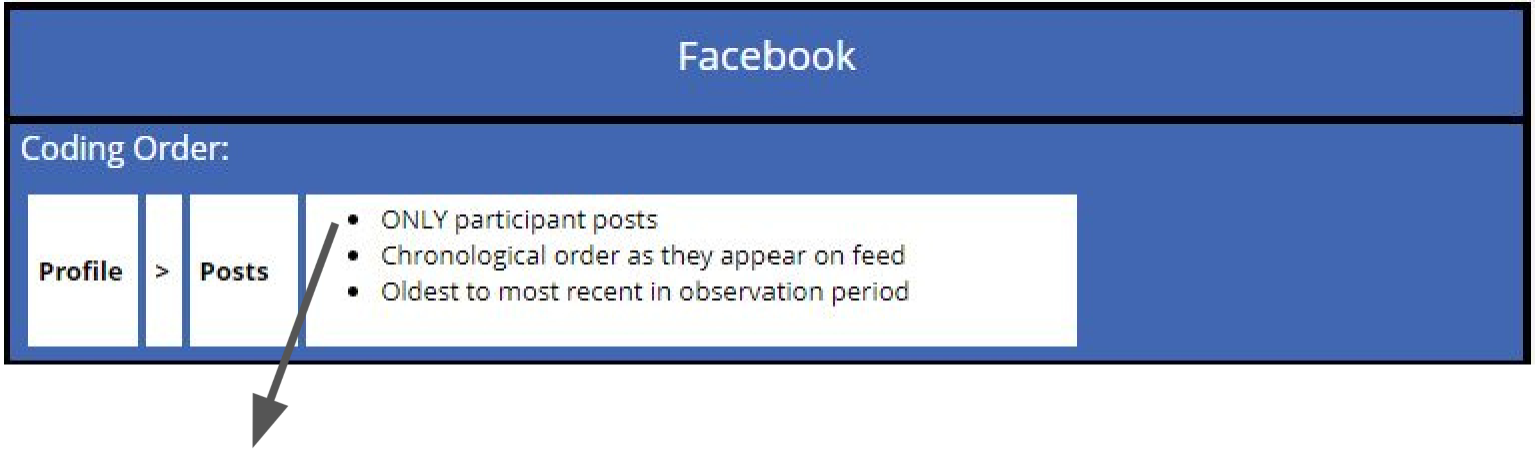

A participant's Facebook page will consist of a collection of all of a Facebook user's posts, as well as the posts from other people that they are connected with on Facebook.
Remember to ONLY code the posts made by the participants themselves.

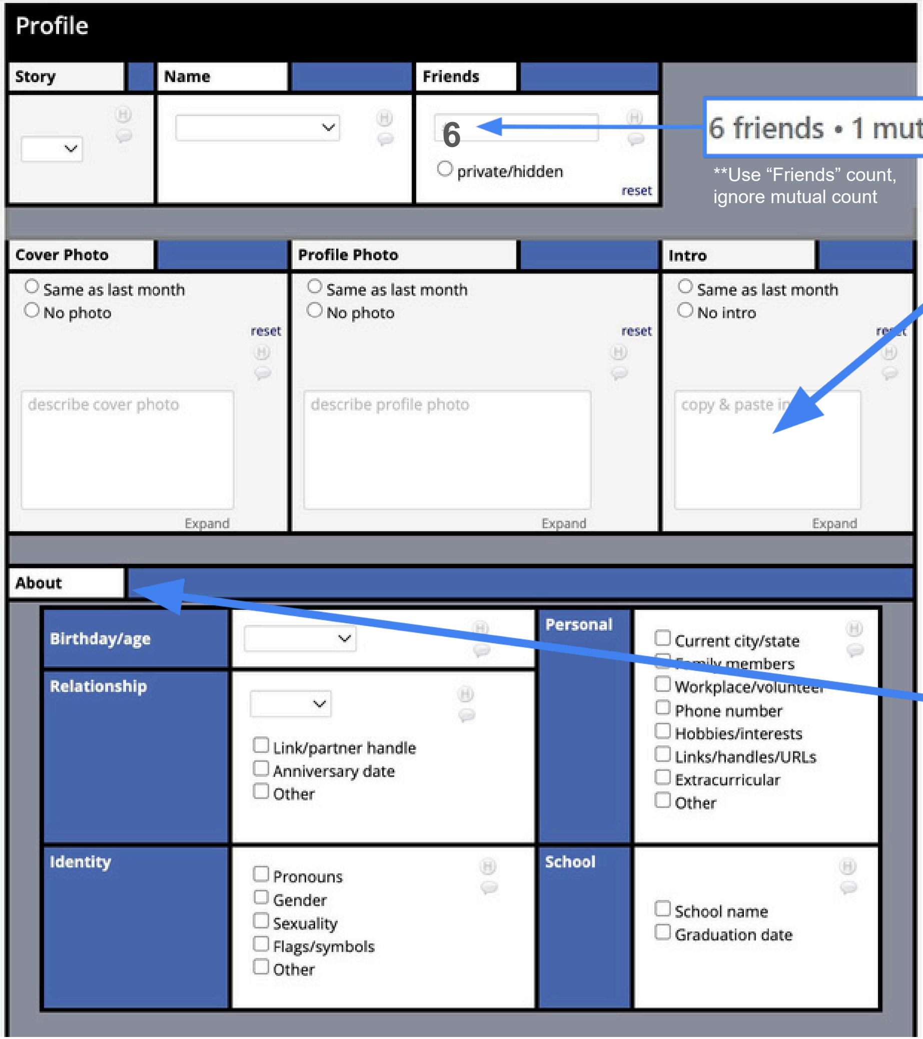

6 friends • 1 mutual

**Use “Friends” count, ignore mutual count

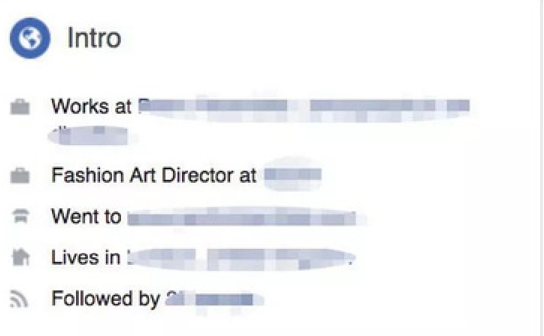

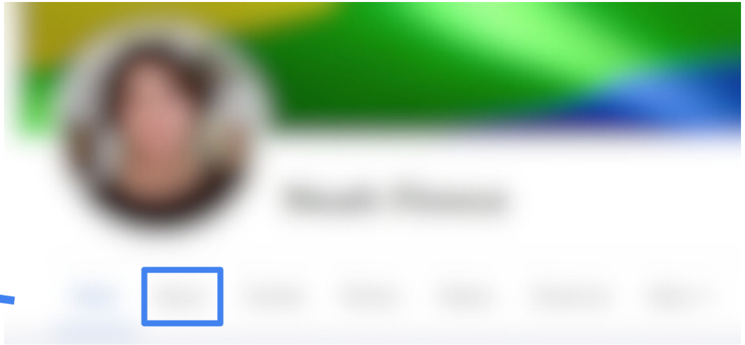

****Click into the “About” section which may contain other details about the participant***

Tagged Posts: # of posts made on the participant's page/wall that are made by OTHERS, not the participant (e.g., friends, family, etc.)

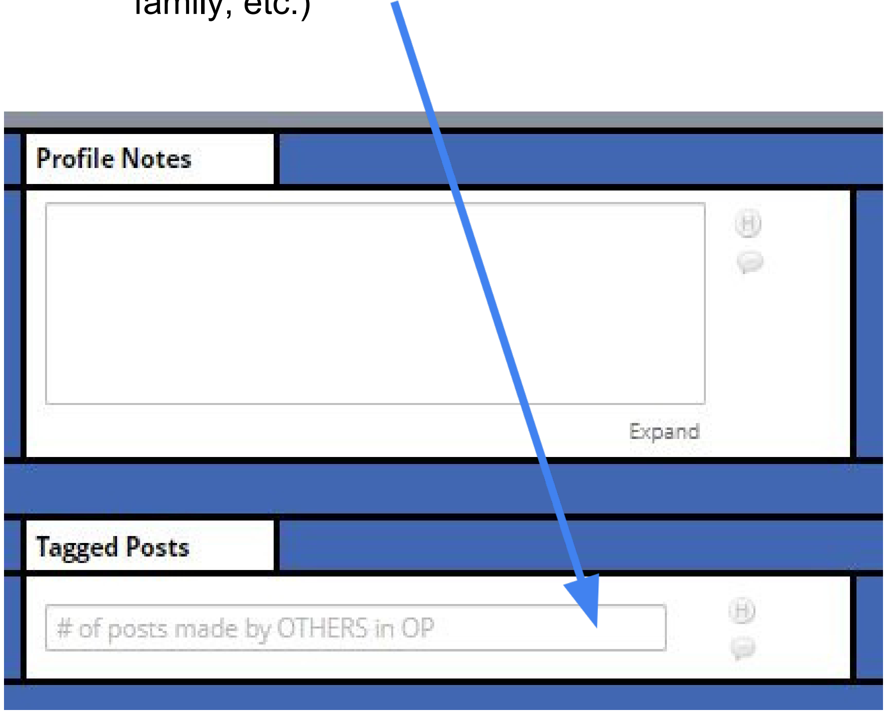

Facebook - Post (by ppt)

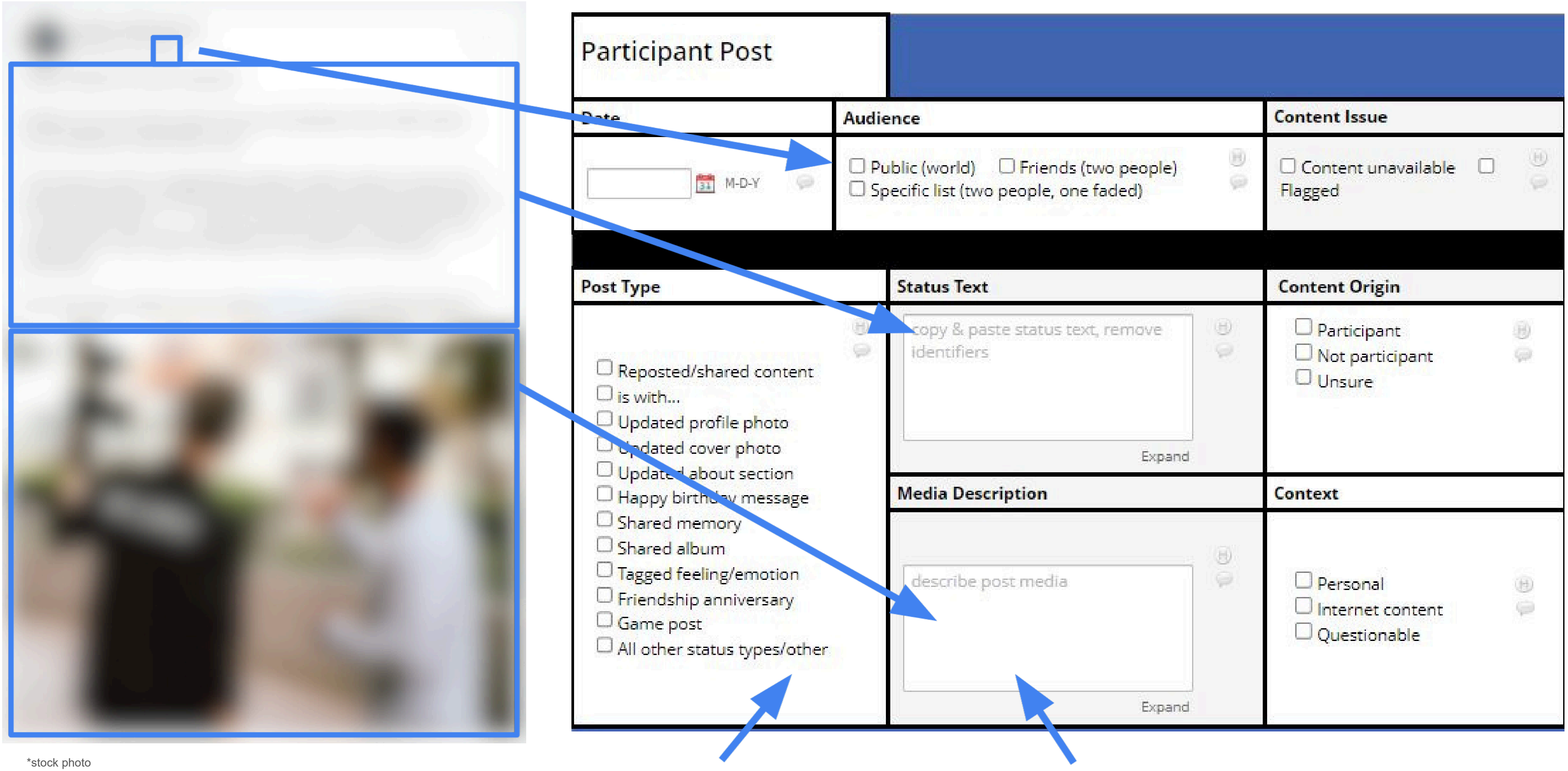

*stock photo

Post type: Can be found on the banner of the post, on the same line as the account name. (e.g., [**Participant**] is with…). Regular posts are coded under "All other status types/other".

Post media: Any photos, videos, or GIFS attached to a post

Types of Facebook reactions:

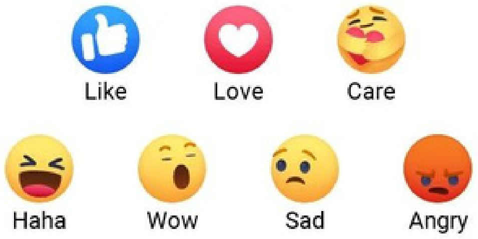

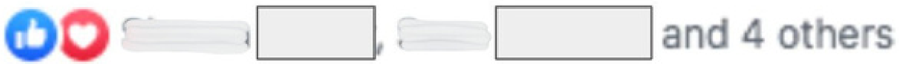

Click on engagement summary to get specific counts. If a type of reaction is not displayed, that means that nobody used that reaction for that post. Therefore, these fields can be marked “0”.

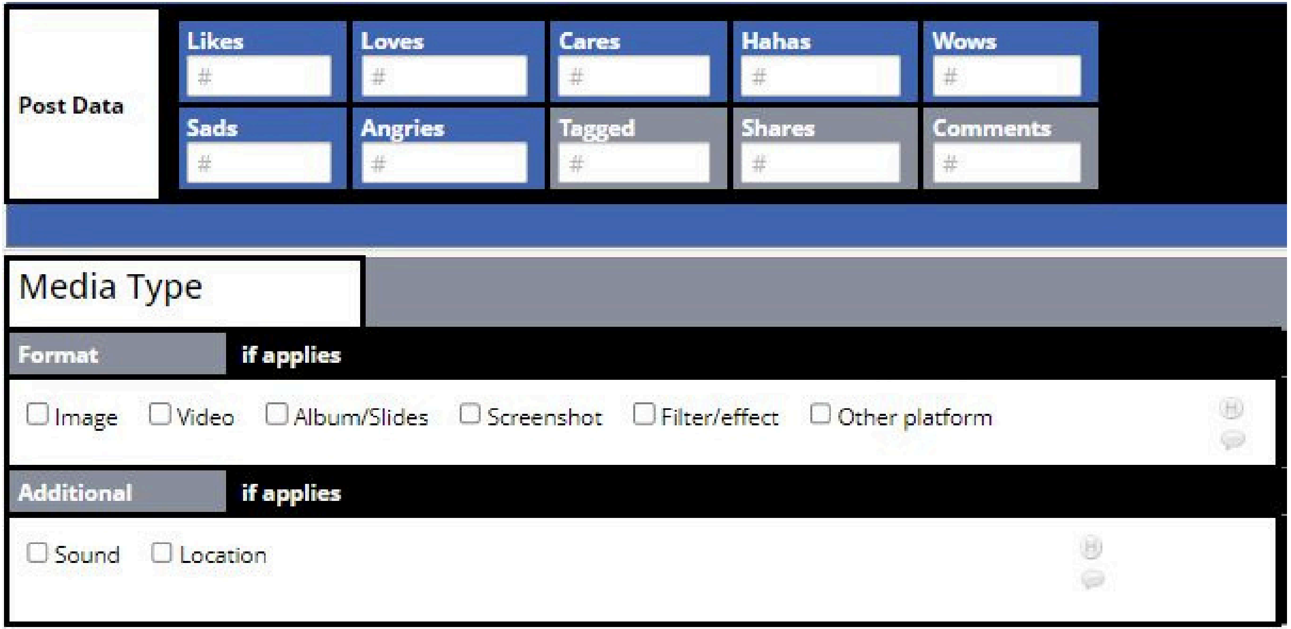

Post Data

Likes	Loves	Cares	Hahas	Wows
#	#	#	#	#
**Sads**	**Angries**	**Tagged**	**Shares**	**Comments**
#	#	#	#	#

Media Type

Format if applies

☐ Image ☐ Video ☐ Album/Slides ☐ Screenshot ☐ Filter/effect ☐ Other platform

Additional if applies

☐ Sound ☐ Location

TikTok Coding

TikTok - Profile

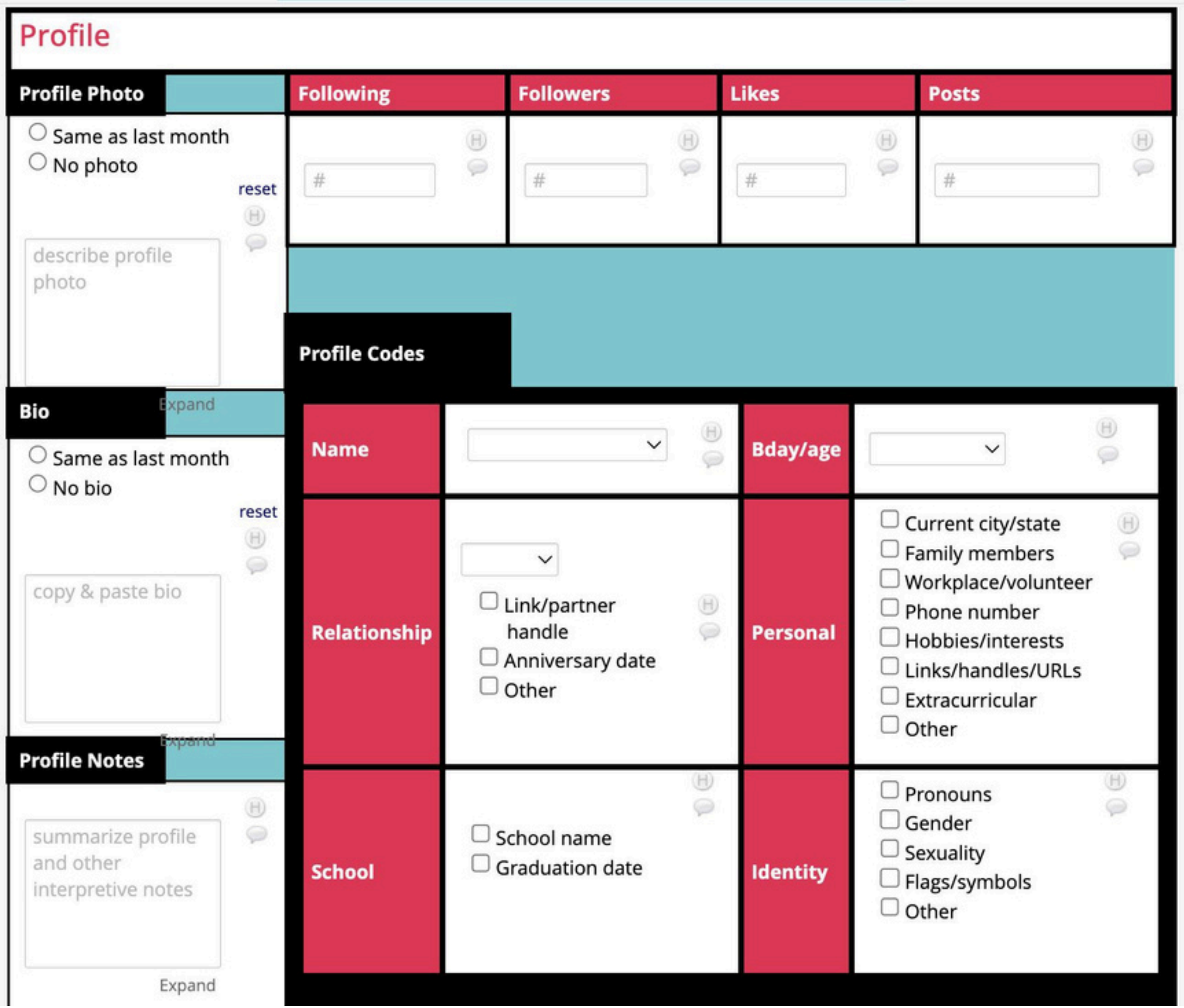
Profile
Profile Photo
Same as last month
No photo
reset
describe profile photo
Expand
Following
#
Followers
#
Likes
#
Posts
#
Profile Codes
Bio
Same as last month
No bio
reset
copy & paste bio
Expand
Profile Notes
summarize profile and other interpretive notes
Expand
Name
Bday/age
Relationship
Link/partner handle
Anniversary date
Other
Personal
Current city/state
Family members
Workplace/volunteer
Phone number
Hobbies/interests
Links/handles/URLs
Extracurricular
Other
School
School name
Graduation date
Identity
Pronouns
Gender
Sexuality
Flags/symbols
Other

TikTok Reposts

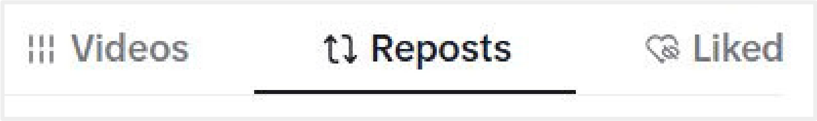

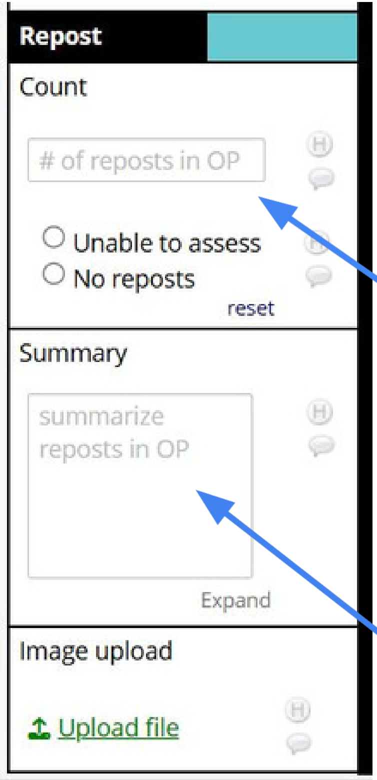

First Month of Coding

Leave “Count” and “Summary” blank. Only complete the “Image upload”. TikTok reposts do NOT show the date they are reposted. Therefore, we need to use a reference post to determine the first (and last) repost made in the OP. Without a reference post, we cannot get an accurate count or summary of TikTok reposts. However, coders DO need to complete the “Image upload” to make this possible in the following months.

All Following Months

Count: Open the profile coding from the previous month. Open the file uploaded in the “Image upload” field. Locate the most recent repost from the screenshot on the ppt’s current repost page and use that as the marker for the BEGINNING of the OP. Every post that appears AFTER that reference post is considered to be made in the OP. Count and enter the number of reposted TikToks in the OP.

- **Unable to assess:** Select if you cannot locate a post from the past OP in the screenshot to use as a marker, and therefore cannot count the number of reposts during this OP. This May occur if posts were deleted, un-reposted, hidden, or if the ppt is a frequent reposter.
- **No reposts:** Select if the Reposts tab is not visible/there are no reposts on the account.

Summary: Describe the common themes in the reposted TikToks posted in the OP

How to Upload a Screenshot

1. Take ascreenshotoftheppt’sTikTokrepostpage (see next slide for example)
    a. Use the computer searchto openthe “Snippingtool”
    b. Click “New”
    c. Drag the window to capture as many of the recent TikTok reposts as possible
    d. Do NOT include ppt’s profile information (i.e., the header with the username,profile picture, and bio) in the screenshot
2. Save the screenshot
    a. Savethe screenshot to the desktop
    b. Rename the screenshot “rec ID_month number_ttreposts” (e.g., 123_m3_ttreposts)
3. Upload the screenshot to REDCap
    a. Inthe “Image upload”field, click “Upload file”
    b. Click “Choose file”
    c. Navigate to the “Desktop” folder and select the screenshot
    d. Click “Upload file”
4. DELETE the screenshot from the computer desktop IMMEDIATELY after uploading to REDCap

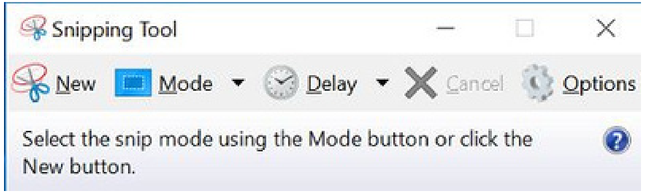

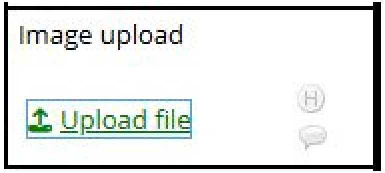

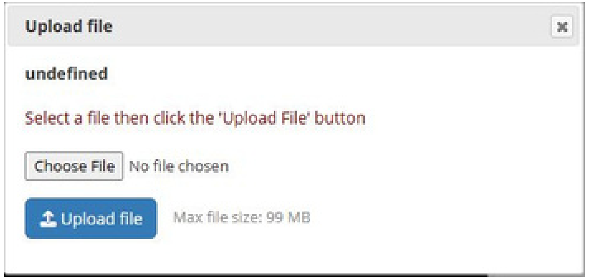

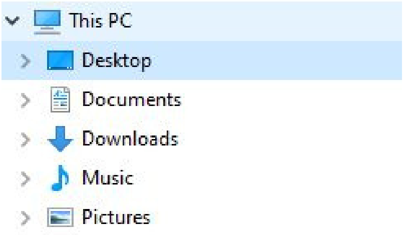

Do NOT include ppt’s profile information (i.e., the header with the username, profile picture, and bio) in the screenshot
DO include as many reposts as possible. To maximize the number of posts shown on the screen, press the three dots on the top right of the Google Chrome browser at zoom out to 80%.

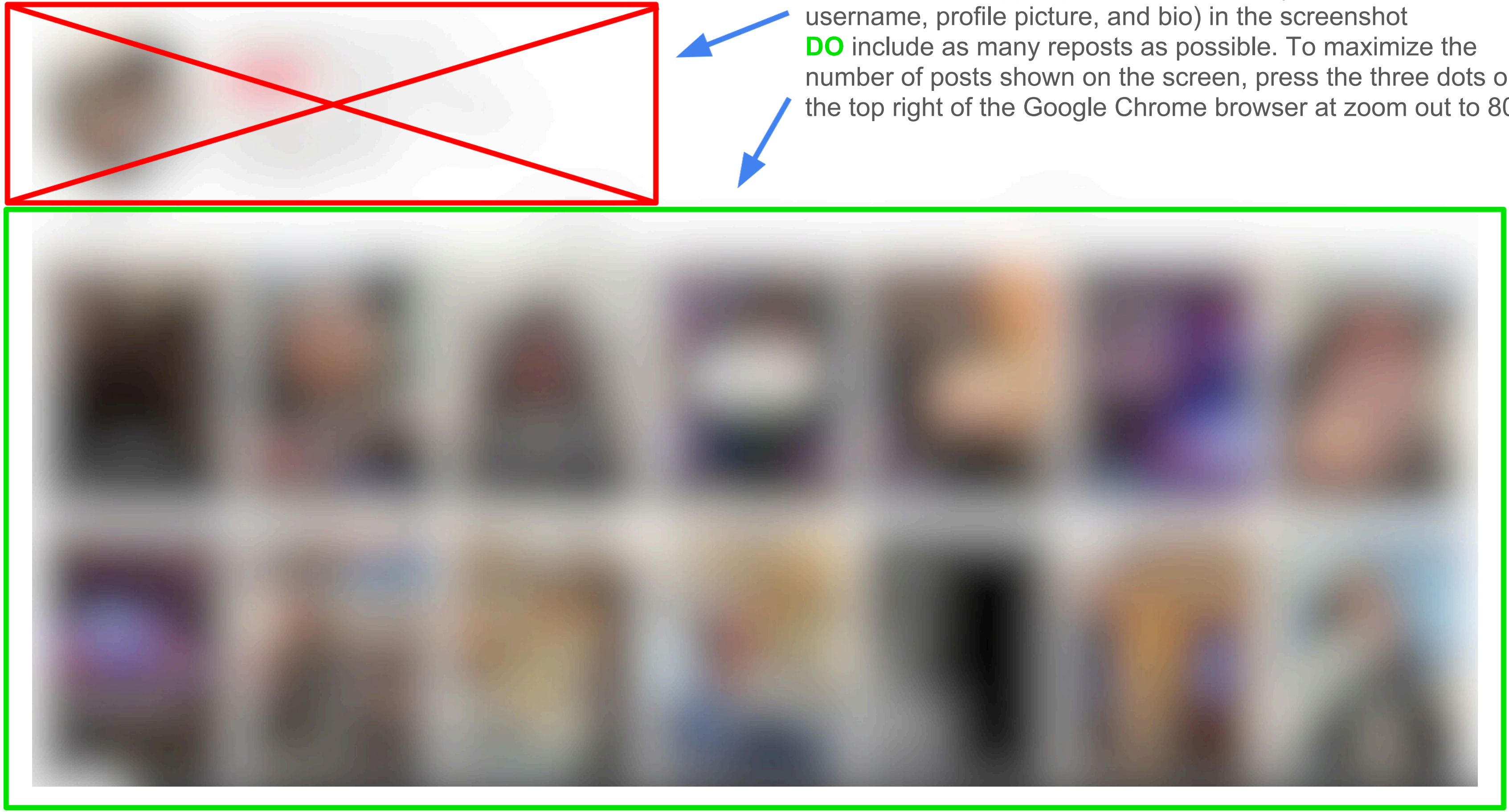

How to View a Screenshot

To view a screenshot of reposts, click the file uploaded. If you get this error, do the following:

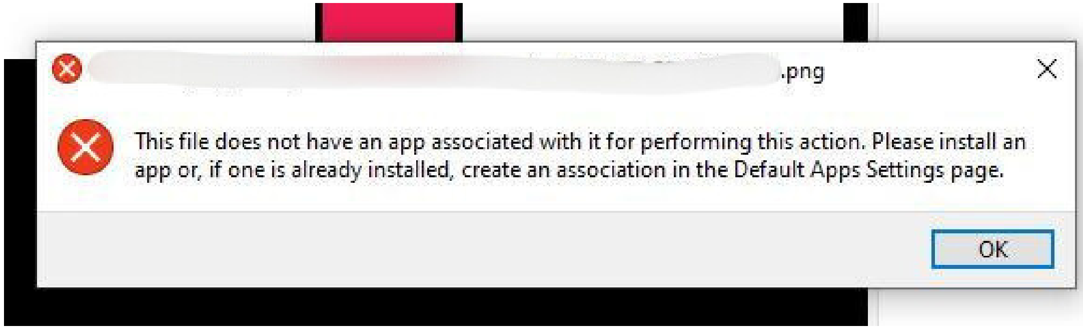

Open File Explorer: Apps > Default Apps > Photos (mountain and moon icon) > Scroll down to .png > Select Photos as the default app

TikTok - Post

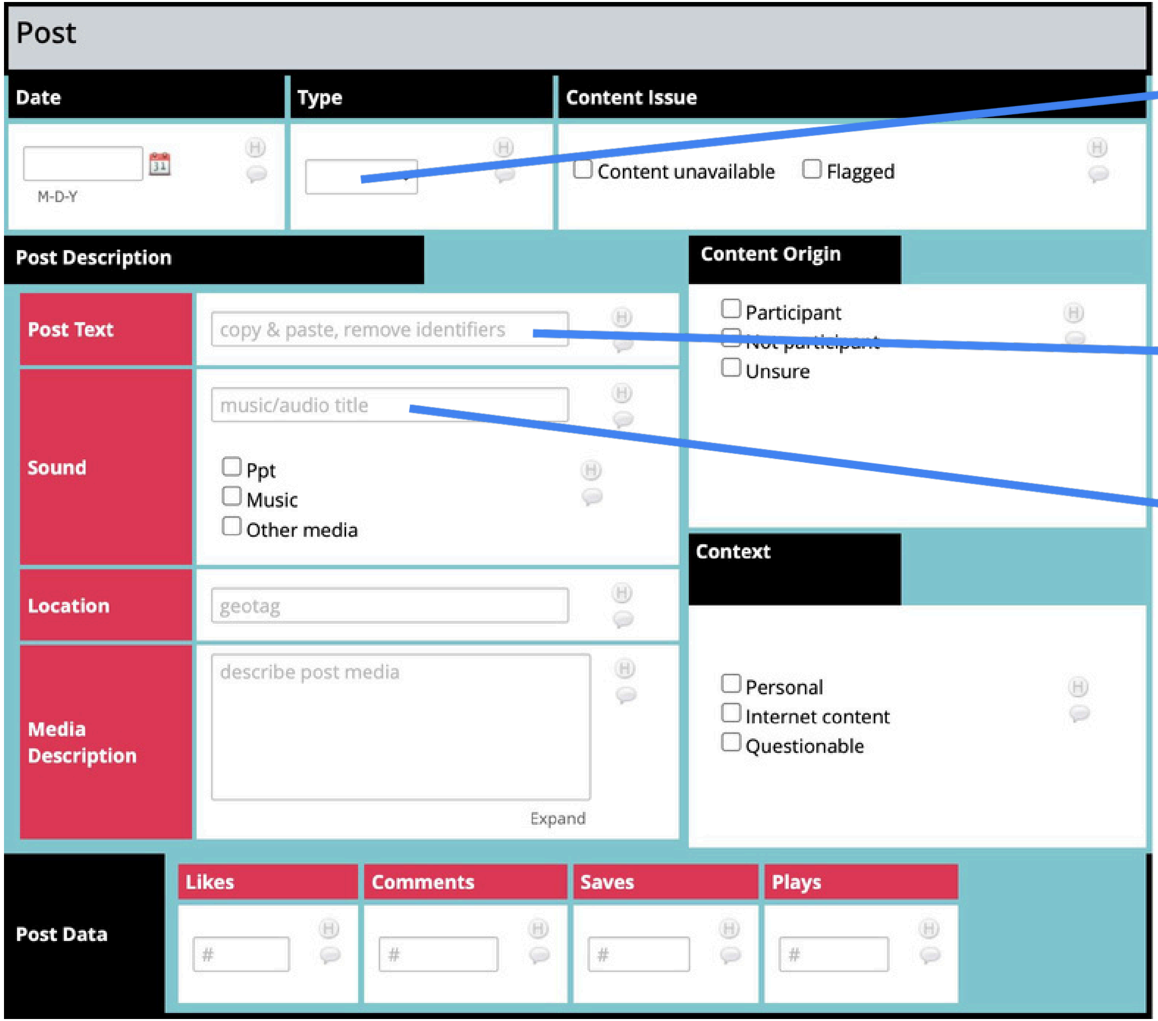

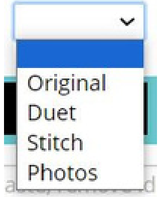

NOTE: Copy/Pasting generally does NOT work on TikTok
Post Text: Post caption. Does NOT include any text written on the screen (write in description).
Sound: Copy and paste sound title as it appears on TikTok. Sound must be integrated into the platform being coded.

- Ppt → “original sound”. Audio
- originating from ppt
- Music → Songs and instrumentals
  Other media → Audio from other sources in media and the internet
- Sound not available → “Sound isn’t available”

If you can’t see the comments when you click on a post, copy the link and paste the URL in a new window. The post will load in a different format that should allow you to see the comments.

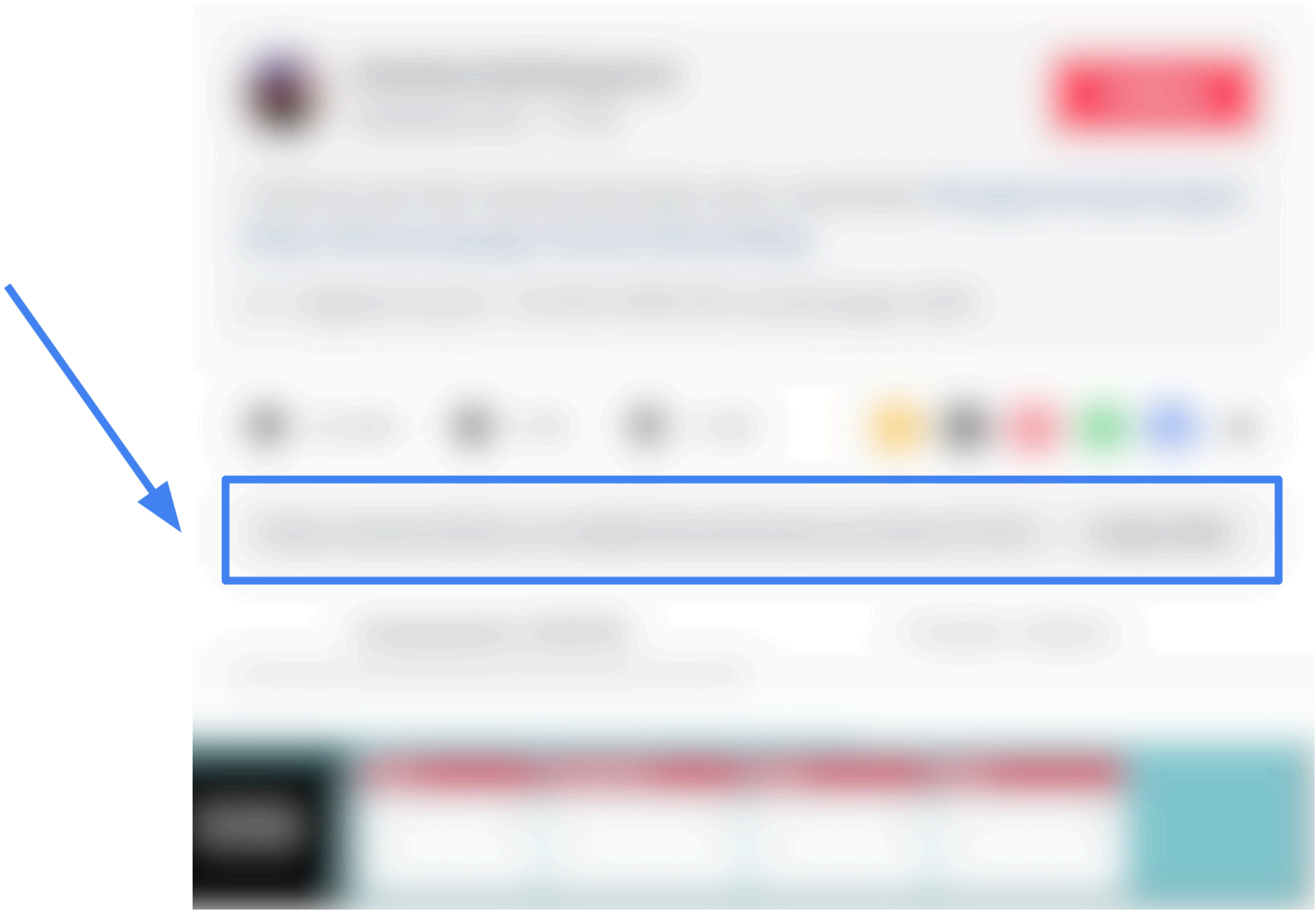

Twitter/X Coding

Twitter - Profile

***Click on “Posts,” “Media” or “Likes” to see the respective count on the top banner of the profile

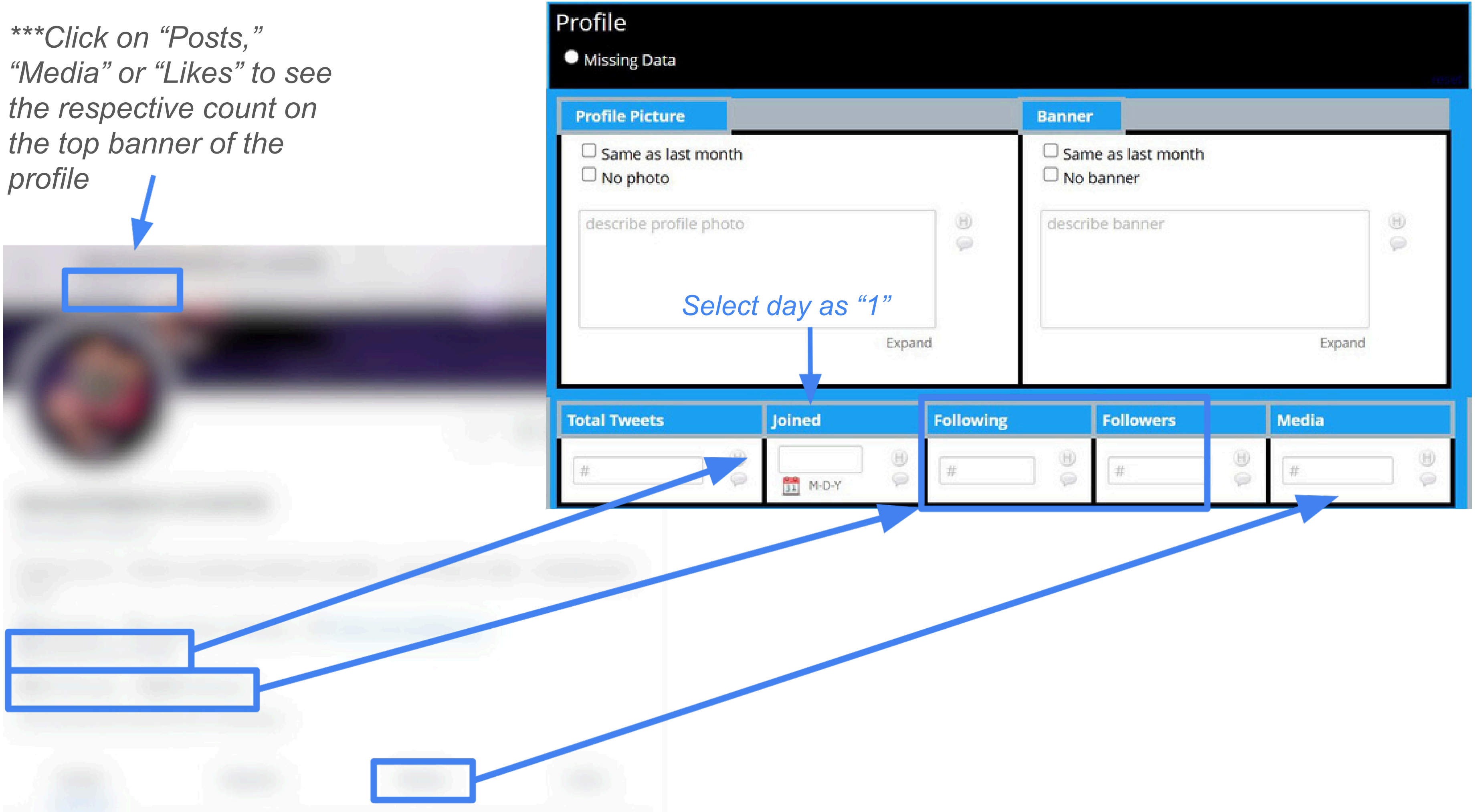

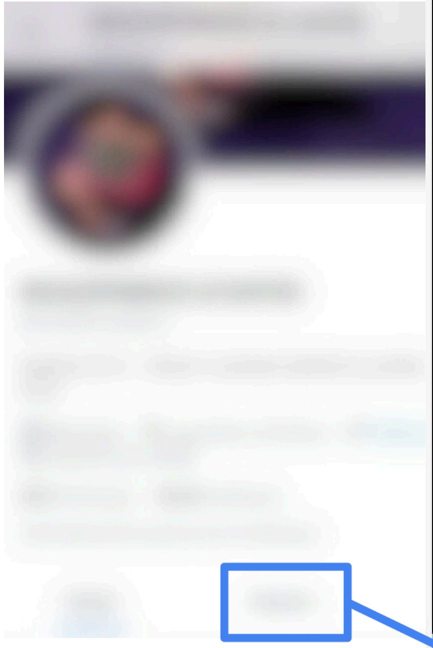

Count the number of replies participant posted in the observation period

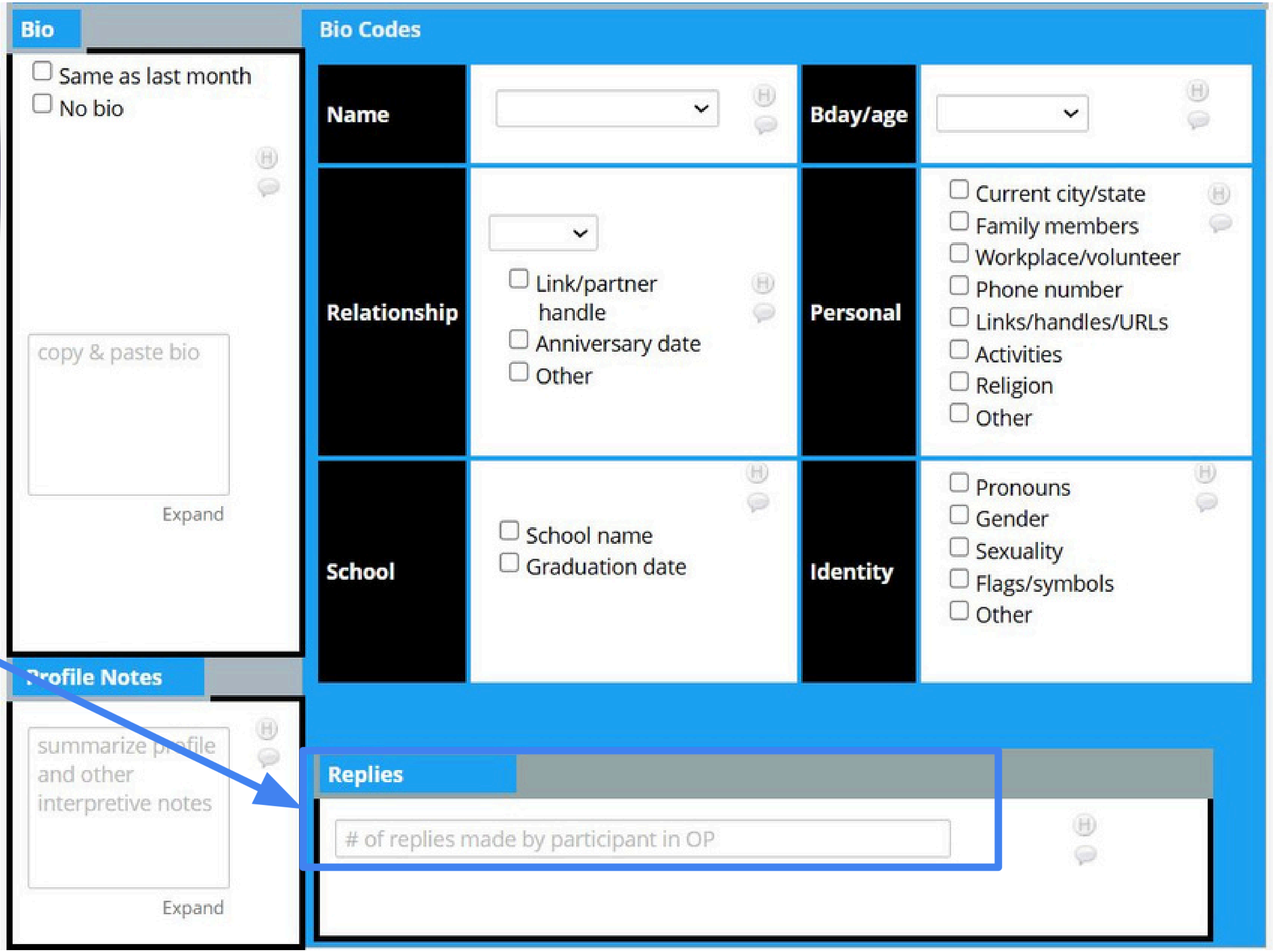

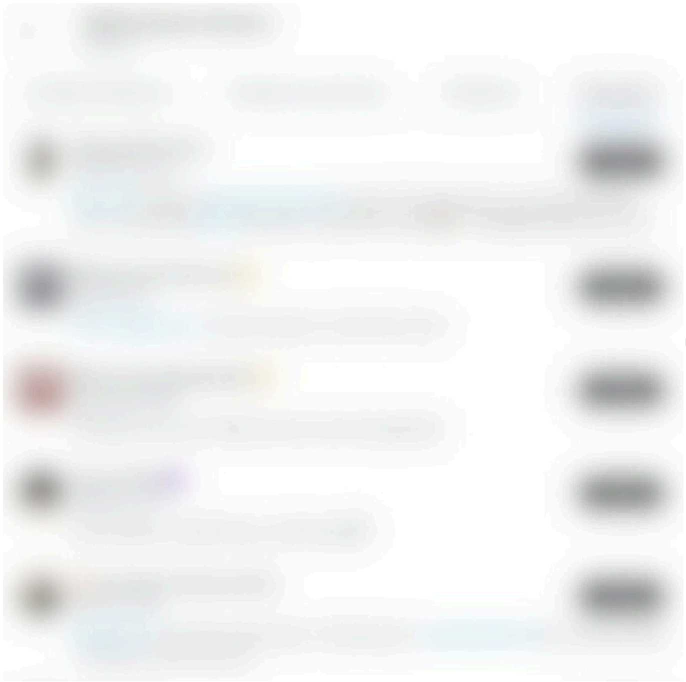

Following lists cannot be copy and pasted from Twitter. Lists will need to be manually transcribed. Only list the handles.

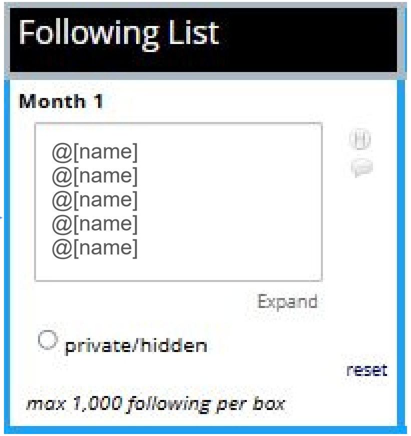

Twitter - Tweet/Retweet/Quote Retweet

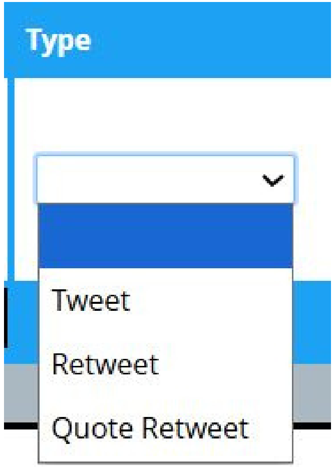

Depending on the type of the post, different "Text" and "Media" fields will appear.

Tweet

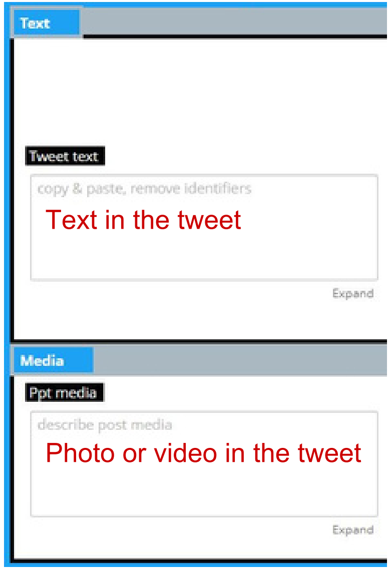

Retweet

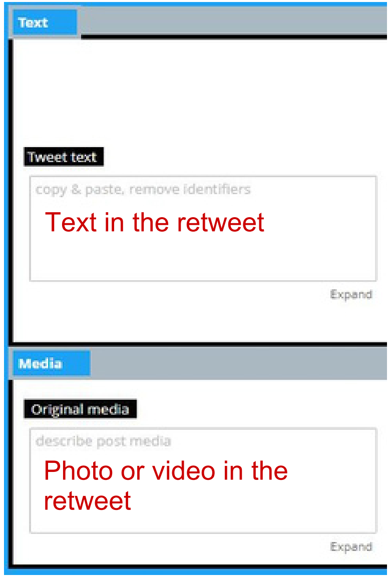

Quote Retweet

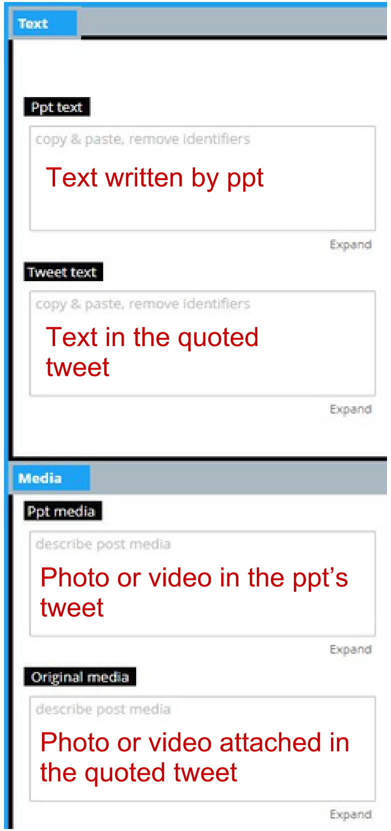

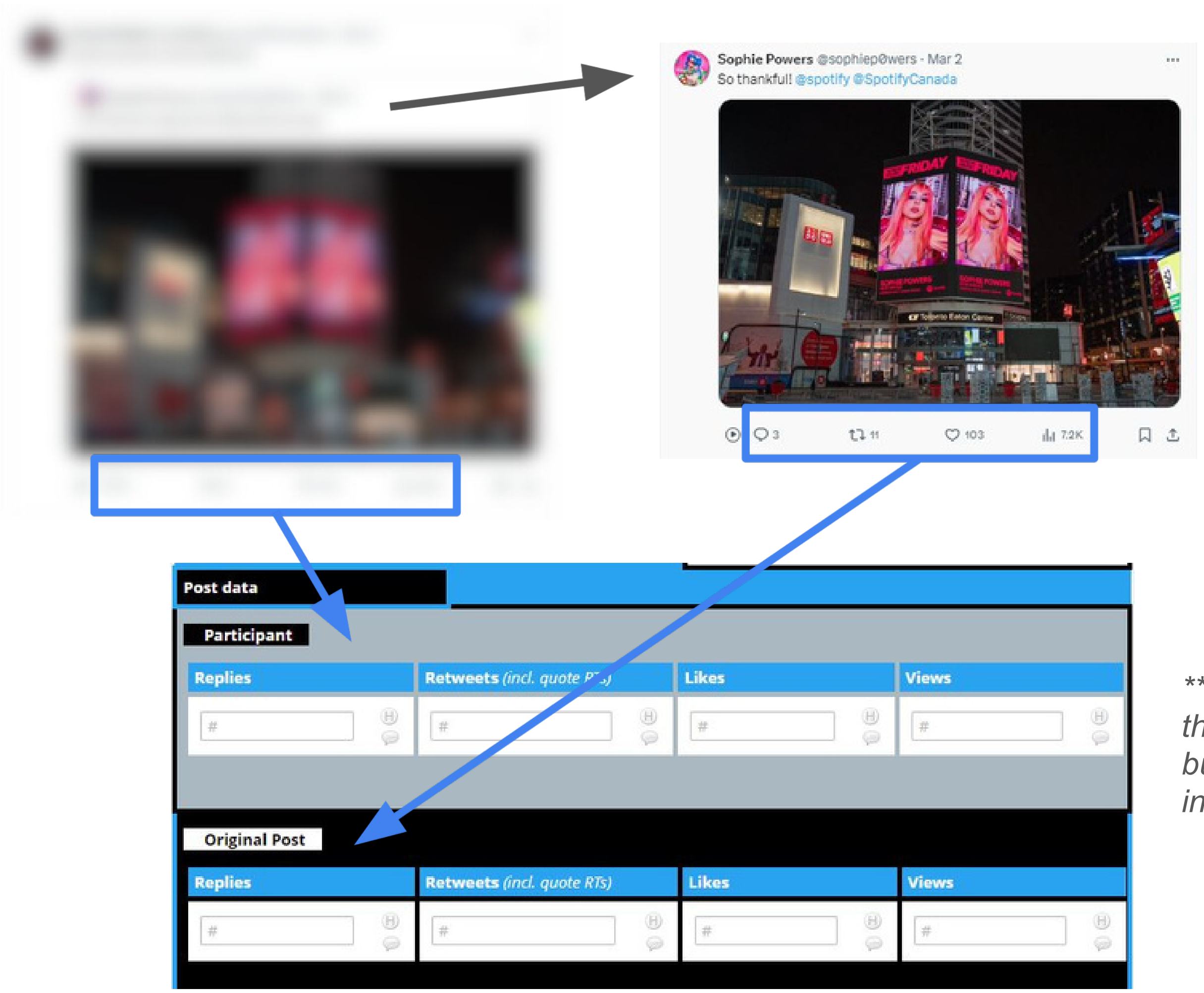

****Views are shown on the ppt's Twitter page, but NOT when you click into the post*

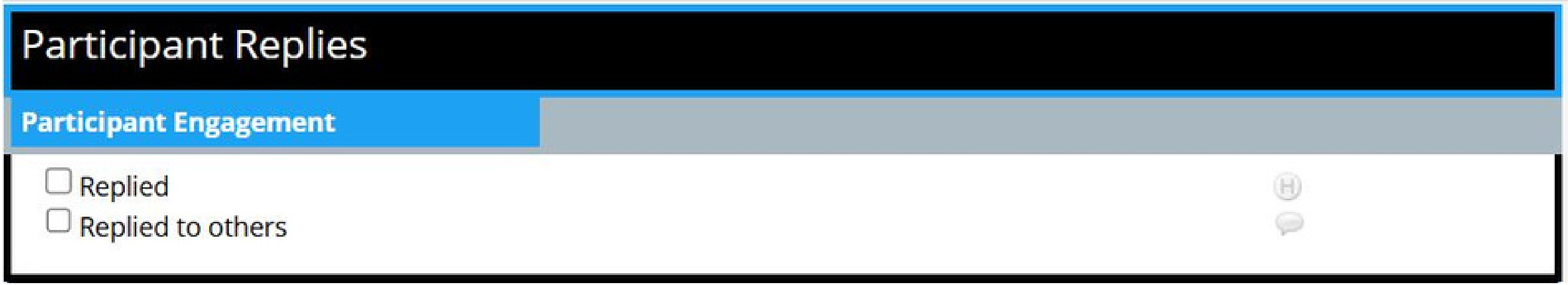

Replied: *Participant replies to themselves on their own post*
Replied to others: *Participant replies to a reply made on another person on their own post*

Considerations for Twitter

For the purposes of this study:

- Referred to as Twitter (not X) to keep language consistent
- Retweets are not necessarily an “endorsement” of a Tweet, and therefore shouldn’t be considered as an “Interest” for the participant (e.g., a participant retweeting a Taylor Swift post does not qualify as a codable “Self-directed” interest in Taylor Swift or her music)
- Tweets and Quote Retweets (where the participant adds their own text) ARE considered to be direct engagement with an interest, and CAN be coded as “Self-directed”
- All portions of a tweet, including the contents of multiple tweets, will be coded in one “Theme” table (e.g., for a quote retweet, the content in a quoted tweet AND the content written by the participant is all codable under the “Theme” codes)

Coding Guidelines

Unit of Analysis

- In qualitative research, the “unit of analysis” refers to the portion of content that will be the basis for coding
- For this project, the “unit of analysis” is the **entiretyoftheindividualsocial media post** being analyzed, including:
  - **Captions**
    Media (photos, videos, GIFs)
  - **Audio**
    Metadata (numbers of likes, views, comments, etc.) *NOTE: Comments themselves can be*
  - *usedforcontext,butarenotindividually codeable(e.g., participant responds to a comment saying“You’remakingmeblush”would NOTbecoded as“Positive MentalHealth”)*
  - **NOT INCLUDED:** Linked content that would require the leaving the platform to view (e.g., YouTube links, VSCO links, etc.)
- The unit of analysis is not the participant themselves, since participants may share content on social media that is not directly related to their own experiences or perspectives

Coding Holistically

- Look through the participant profiles to learn contextual information about the participant to help code posts accurately (e.g., using other posts on a participant's account to determine whether a person in their photos is a peer or family member)
- Coders are often assigned the same participants for repeating months so they can use previously learned information about the participant in subsequent coding
- Use the internet to look up basic information to assist with coding (e.g., Googling the source of a meme), but do NOT use PII
- You can use public data to make your theme determinations (e.g., users that are tagged frequently). Do not **record** data from people who have not consented to be in the study (e.g., other users)

How much detail should be used?

- Different posts will require different amounts of explanatory text
- Use the size of the text box as a rule of thumb
- Describe the subject and setting of the post
- When describing a post, ask yourself: *"Am I using enoughdetailthat another researcher looking at thisdatainthe future could accuratelyunderstand all aspects of this post, without seeing it?"*

What information should be coded?

ProfileDescription/Notes

- Explore the entirety of the account (e.g., Likes, Tagged, About section, etc.)
- Describe content posted by participant and their posting frequency

Post Media

- Describe ALL photos/videos included in a post (individually or summarize)
- Describe both the visual and audio elements of the post
- Always transcribe verbatim in quotation marks:
    - Any text superimposed on a post (i.e., text that cannot be copy/pasted)
    - Dialogue and lyrics

Post Text

- Copy and paste entire captions directly into the REDCap form
- Do NOT use quotations for caption text that is copy/pasted

When to use 0s, blanks, and numbers

- 0s → Numerical fields where the count is 0 (e.g., a post with no comments)
- Blanks → The field is not shown on the platform (e.g., a post with views not shown)
- Numbers → Use full numbers with NO abbreviations (1k → 1000)

Copy/pasting difficulties

- TikTok often has issues with copy/pasting
- If text is unable to be copy/pasted directly, it must be transcribed verbatim by hand
- Emojis can be described in brackets (e.g., [red heart emoji]) or can be found and copy/pasted from Emojipedia

Redacting PII

- Always DE-IDENTIFY any personally identifying information (PII)
- Replace PII with a description of the information in brackets:

  Examples:
  West High School → [Ppt’s high school]
  @mysnapchat → [Ppt’s Snapchat]

- Redact information of individuals personally known by the participant *(friends, family, etc.)*
- Do NOT redact names of public figures *(celebrities,influencers,content creators, etc.)*

Coding Protocol

Opening a Query

Ifyou encounter anissueor have a question regarding a particular field while coding, open a query on REDCap:

1. Click the speech bubble icon on the right side of the field
2. Click “Open query”
3. Assign the query to [staff]
4. Write your question in as much detail as possible in the
5. “Comment” field

   Click “Open query”

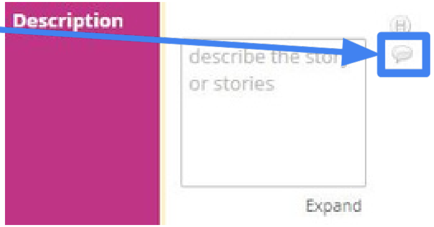

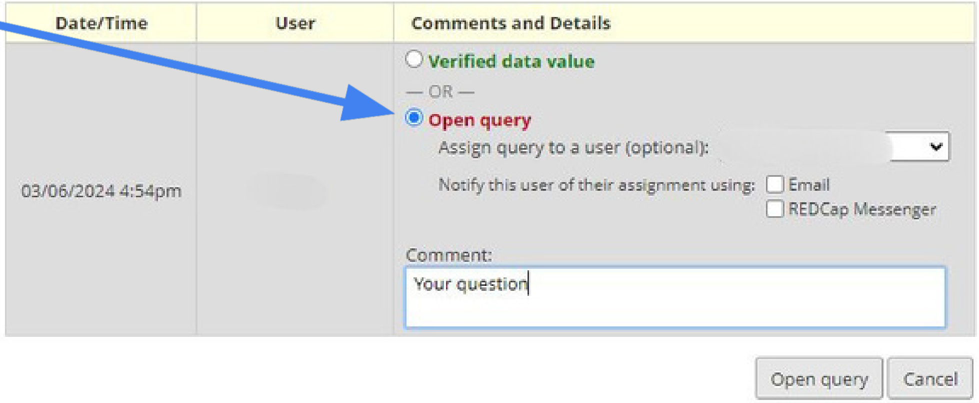

[staff] checks queries **daily** and will reply back with answers via Teams or via the query that they were tagged in.

Questions that need immediate answers can be sent to your supervisor via Teams.

Coding Workflow

1. Assigned coding
   a. Assigned dailyby[staff] according to the coding due on they day of your shift
   b. Under “[Your Name]’s Assigned”
2. Delayed report list
   a. Coding that was not completed on time and marked as “Delayed”
   b. Under “Reports”, click “Delayed”
   c. Before working on this list, contact [staff] and work with other coders working during your shift to make sure you are not working over each other.

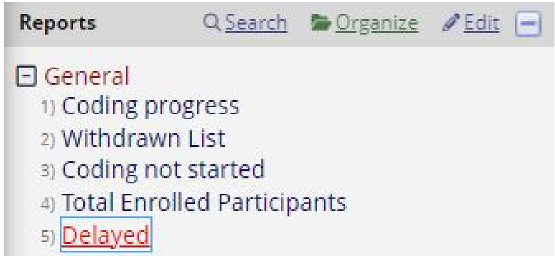

Delayed

Record ID<br>record_id	Event Name<br>redcap_event_name	# months delay<br>prog_delay
364	Info	1
490	Info	1
493	Info	1
583	Info	1

Double-Checking Assignments

When you are working through your coding, be sure to check for:

- Red queries
  - Under the “Coder” column,check forthe speechbubble icon with a red circle.
  - This indicates an open unresolved query, meaning that the coding is assigned to someone else.
  - To check the assignment, click the icon to open the data resolution workflow to see the coder assigned.

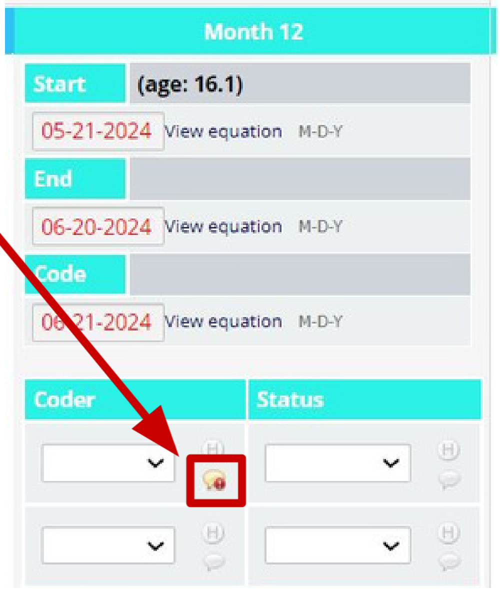

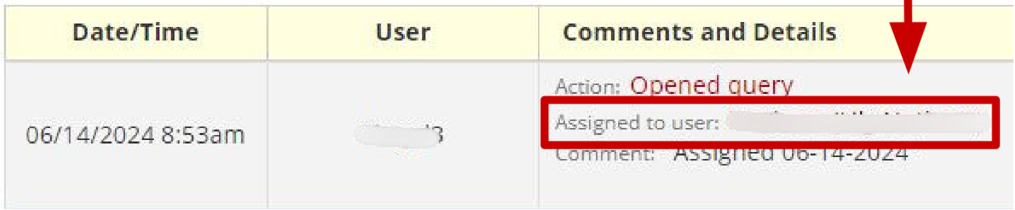

Double-Checking Assignments *(cont.)*

When you are working through your coding, be sure to check for:

- Coding dates
  - Makesure to code all posts made ON OR AFTER the “Start” date, and ON OR BEFORE the “End” date.
  - The “Code” date is NOT included. It only marks the date the participant will appear on the coding calendar, since the observation period has officially passed on that date.
- Month column
  - Always check the Info form to confirm which month has been assigned to you for coding.
  - Do NOT assume the next column with empty bubbles is the month assigned for coding.

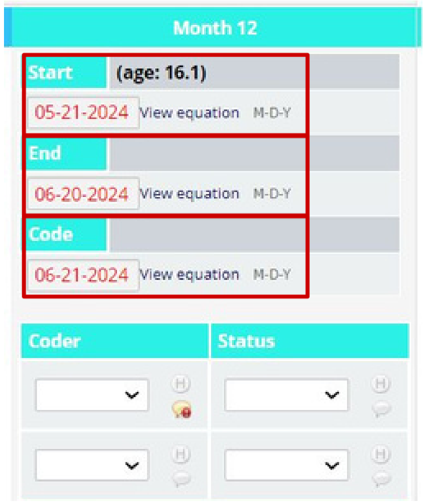

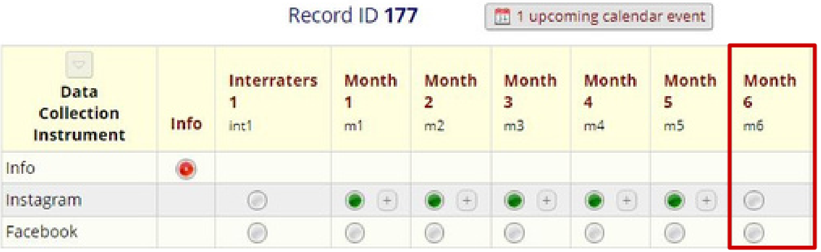

Delayed vs. Back Coding

- Delayedcoding:Codingprevious months that were missed/not completed
- Back coding: Applying new codes to previously coded material

Finding Missing Profiles

Why may you be unable to find an account?

If you can’t find an account, it may be because:

- Most likely:
  - Username changed
  - The account is not followed by the whatever account you are using. A majority of participants’ accounts are followed by [team social media account #1].
  - However, some accounts are only followed by [team social media account #2] or [team backupsocial media account].
- Less likely:
  - Account is private and the participant hasn’t approved our follow request
  - Account was deactivated, deleted, or banned
  - Search restrictions or visibility limits
  - Typographical errors

Finding Missing Accounts

Ifyou are assignedaprofileandcannotlocatethe account, there are several different methods you can use to try to find it:

- **Check the notes on the "Social Media Info" section on the REDCap:** Any special directions or notes about this account will be located here.
- **Check other study accounts:** Check the [name] or [name] accounts to see if they are following the correct participant account.
  - You cancheck and search the followinglistsof these accounts WITHOUT logging into these accounts.Since each account follows each other, you can navigate to the profile, click "Following," and search this list.
  - Try to avoid logging in and out of different study accounts, as this runs the risk of our study accounts being flaggedfor "bot behavior" and being bannedfrom the platform.
- **Using participants name in the search bar:** Locate their name at the top of the Tracking form. If you suspect a participant goes by a nickname, you can use this too.
- **Check the "Bio" section of their other profiles:** Some participants link their other accounts or use the same bio across different platforms.

Verification Methods

If you find a profile you think might be the missing profile, do the following:

- Check to be sure it's NOT:
  - **Anotheraccountthattheparticipanthasalreadysharedwithus:** Check the REDCap "Social Media Info" form to see if the participant has shared additional accounts on the same platform with the study team. Confirm that the account you found is not just a different shared account.
  - **A different account that the participant has NOT shared with us:** The participant may have an account they haven't shared with us, which we are not allowed to view. This could be the case if the account is not followed by any study accounts. For documenting this account, follow the instructions on this slide.
  - **An account owned by a different participant:** Do a REDCap search of the username to makesureitdoesn't comeup as belongingtoanother participant.
- Check the latest coding form for the platform to find matching posts, friend/follower count, or bio. If this information is similar to what you currently see on the account, you have likely found the correct profile.

If you CAN find the account:

- Update the “Social Media Info” form withthe new username and URL redirecting to the account page.
- Add a note to the Notes box with the date, actions taken, and your initials (e.g., “[Date]: Ppt changed username. Located account and updated account information. -[Your Initials]”).
- Code the account as usual.

If you CANNOT find or view the account:

1. Mark as “Missing data” for profiles.
    a. Open a “Profile” form and click “Missing Data”.
    b. In the “Profile Notes” box, write “Account not found” or “Account private”.
2. Open query for [staff] in REDCap:
    a. Locate the participant’s Record ID on the REDCap.
    b. Open the “Social Media Info” form.
    c. On the “URL” field, open a query and assign it to [staff]
    d. In the comment box, write a note that details the actions you took to locate the account (e.g., [Date]: Account missing/private. Did X, Y, and Z, but could not locate. -[Y/N]
3. The R&R team will reach out to the participant to confirm the status of the account.

If you find a participant’s account that we do not follow:

- Open a query under for [staff] on the Social Media Info form.
- List the additional account so that the we can ask the participant about it during their next update call.

Concerning & Uncomfortable Content

Concerning Social Media Content

Because two of our PIs are mandated reporters, we have a legal and ethical responsibility to ensure compliance with mandated reporting requirements.

Posts are to be considered "concerning content" if they reference:

- Suicidality or self-harm
- Sexual or physical abuse
- Threat of harm or violence towards others

If you see concerning content, do the following:

- Follow the Concerning Social Media Content Protocol for instructions on how
- to identify, document, and report concerning content.
  Select the "Risk/Harm Behavior" code.

Viewing Uncomfortable Content

- Participant content is *not* screened by staff before it is assigned to coders
- Coders may encounter content that is upsetting or uncomfortable (e.g., profanity, sexually suggestive content, cultural insensitivity, etc.)
- While we cannot guarantee that coders will not encounter uncomfortable content, coders are under no obligation to code this content.
- If you are assigned a participant that you prefer not to code, tell your supervisor and the participant will be reassigned.

Ethical Conduct

Ethical Conduct

- Platforms & Accounts
  - Coding should bedone on department computers ONLY.
  - All social media data should stay in REDCap.
    - No downloads onto any computer.
    - When referencing participants OUTSIDE of REDCap, use the ppt's ID number.DoNOT useparticipants' account or name.
- Using Research Profiles
  - NEVER interact with participants content (like, message, etc.)
  - Log-ins for the social media accounts should NOT be saved and can only be kept here.

Ethical Conduct (cont.)

- Respect & Confidentiality
  - Berespectful ofppt'scontent when recording data and in discussions with the research team.
  - Social media information should only be shared with the research team, and nobody else.
- Protocol
  - Never falsify data.
  - Report any concerning content (suicidality, abuse, etc.) to your supervisor.
  - Report protocol violations to your supervisor. Accidents happen–the best thing you can do to protect ppts is to let us know.

End of Coding Guide!

If youhaveanyquestions,pleasecontactyoursupervisor.
